# PER2 reprograms intracellular cholesterol synthesis to inhibit oral squamous cell carcinoma and the chronotherapeutic efficacy of simvastatin

**DOI:** 10.1038/s41420-026-03209-5

**Published:** 2026-06-20

**Authors:** Shilin Yin, Fang Yang, Zhiwei Zhang, Mingyuan Liu, Hong Tang, Kai Yang

**Affiliations:** 1 Department of Oral and Maxillofacial Surgery, The First Affiliated Hospital of Chongqing Medical University, Chongqing, China; 2 Department of Stomatology, Chongqing General Hospital, Chongqing University, Chongqing, China

**Keywords:** Oral cancer, Head and neck cancer

## Abstract

Dysregulation of cholesterol homeostasis is closely associated with the development of various cancers, but the underlying mechanisms remain unclear. This study found that the circadian clock gene Period 2 (PER2) is downregulated in oral squamous cell carcinoma (OSCC), promoting intracellular cholesterol synthesis and cell proliferation. Conversely, PER2 overexpression inhibits cell proliferation by suppressing intracellular cholesterol synthesis. Mechanistically, PER2 binds to Myosin Heavy Chain 9 (MYH9) and recruits Tripartite Motif Containing-21 (TRIM21) to promote ubiquitin-dependent degradation of MYH9, thereby inhibiting the cholesterol synthesis rate-limiting enzyme 3-hydroxy-3-methylglutaryl-CoA reductase (HMGCR), reducing intracellular cholesterol synthesis, and suppressing OSCC proliferation. In OSCC xenograft models, overexpression of PER2 or treatment with the HMGCR inhibitor simvastatin significantly inhibited OSCC growth, with the combination showing markedly enhanced anti-OSCC efficacy compared to either approach alone. To explore the circadian regulatory role of PER2, overexpression or silencing experiments in OSCC demonstrated that PER2 modulates the circadian rhythms of HMGCR expression, intracellular cholesterol, and cell proliferation. Further investigations in subcutaneous OSCC mouse models revealed sex-specific circadian rhythms in PER2 and HMGCR expression, intracellular cholesterol, cell proliferation, and tumor growth in OSCC cells. Specifically, the acrophases of these indicators in OSCC of male mice were delayed relative to females, and the mesors of HMGCR expression, intracellular cholesterol, and cell proliferation were significantly higher than in females. The anticancer efficacy of simvastatin against OSCC in mice varied up to 2.5-fold across six different time points within 24 h, with broader time windows for optimal therapeutic efficacy and adverse effects in female mice than in males. This study identified a novel mechanism by which PER2 reprograms intracellular cholesterol, demonstrating that PER2 upregulation combined with simvastatin represents a potential novel strategy to improve the prognosis of OSCC patients, and clarifies that the anti-OSCC effect of simvastatin exhibits time-dependent and sex differences.

## Introduction

Oral squamous cell carcinoma (OSCC) accounts for approximately 90% of oral cancers [1], with more than 380,000 new cases each year, 180,000 deaths annually, and an increasing incidence in recent years [2]. Primary treatment methods for OSCC include surgical resection, radiotherapy, chemotherapy, and immunotherapy [3]. However, the 5-year survival probability for OSCC patients stands at approximately 50%, with no substantial improvement in the past 30 years [4]. Therefore, it is critical to clarify the molecular basis of the pathogenesis of OSCC and explore new treatment strategies.

Cholesterol, as well as serving as an essential structural component of cell membranes, actively participates in cellular processes including cell proliferation, metabolism, and autophagy [5–7]. Cholesterol homeostasis is thus crucial for maintenance of normal cellular physiological activities, and its dysregulation has been implicated in the development of various cancers [6–9], but the underlying mechanisms remain unclear. Current research shows that cholesterol biosynthesis in vivo is regulated by the circadian clock [10–12]. The circadian clock is an endogenous time-keeping system that controls and optimizes physiological processes within the organism, consists of a series of clock genes [13, 14]. These genes form negative feedback loops such as CLOCK/BMAL1-PERs/CRYs, thereby generating circadian rhythms with a period of approximately 24 h [14, 15]. Circadian clock genes regulate many crucial physiological processes, including metabolism. They ensure that these processes are coordinated and orderly in time and space, thereby maintaining organismal homeostasis.

Dysregulation of these genes may contribute to the development of various diseases, including cancer [15–17]. Period 2 (PER2) is a core circadian clock gene [14], and its expression exhibits circadian rhythmicity [18, 19]. Studies have demonstrated that PER2 is downregulated in various cancers, including OSCC, and its expression level is significantly correlated with patients’ survival time [20–22]. Therefore, we hypothesize that PER2 plays a critical regulatory role in intracellular cholesterol metabolism in OSCC. Further in-depth studies may provide novel insights into the pathogenesis and therapeutic strategies for OSCC and other cancers.

This study found that PER2 inhibits OSCC by reducing intracellular cholesterol synthesis. The underlying mechanism involves PER2 binding to myosin heavy chain 9 (MYH9) and recruiting E3 ubiquitin ligase tripartite motif containing-21 (TRIM21) to promote ubiquitin-dependent degradation of MYH9, thereby suppressing expression of 3-hydroxy-3-methylglutaryl-CoA reductase (HMGCR), the key rate-limiting enzyme in cholesterol synthesis, to reduce intracellular cholesterol synthesis and inhibit OSCC cell proliferation. In the OSCC xenograft model, it was demonstrated that the combination of PER2 overexpression and the HMGCR inhibitor simvastatin significantly enhanced the anti-tumor effect compared to monotherapy. Further in vitro and in vivo combination experiments revealed that PER2 regulates the circadian rhythms of HMGCR expression, intracellular cholesterol levels, and cancer cell proliferation, with notable sex differences. Additionally, the anti-cancer efficacy of simvastatin in OSCC treatment through cholesterol inhibition varied by up to 2.5-fold across different time points within a 24-h period. We further identified sex-specific optimal therapeutic time windows that maximized efficacy while minimizing adverse effects. This study not only elucidates a novel mechanism by which PER2 reprograms cholesterol metabolism to suppress OSCC, but also provides a rationale and framework for developing precision cancer treatment strategies based on circadian rhythm-associated sex and time differences.

## Results

### *PER2* expression is downregulated in OSCC and negatively correlated with intracellular cholesterol

**Fig. 1:**
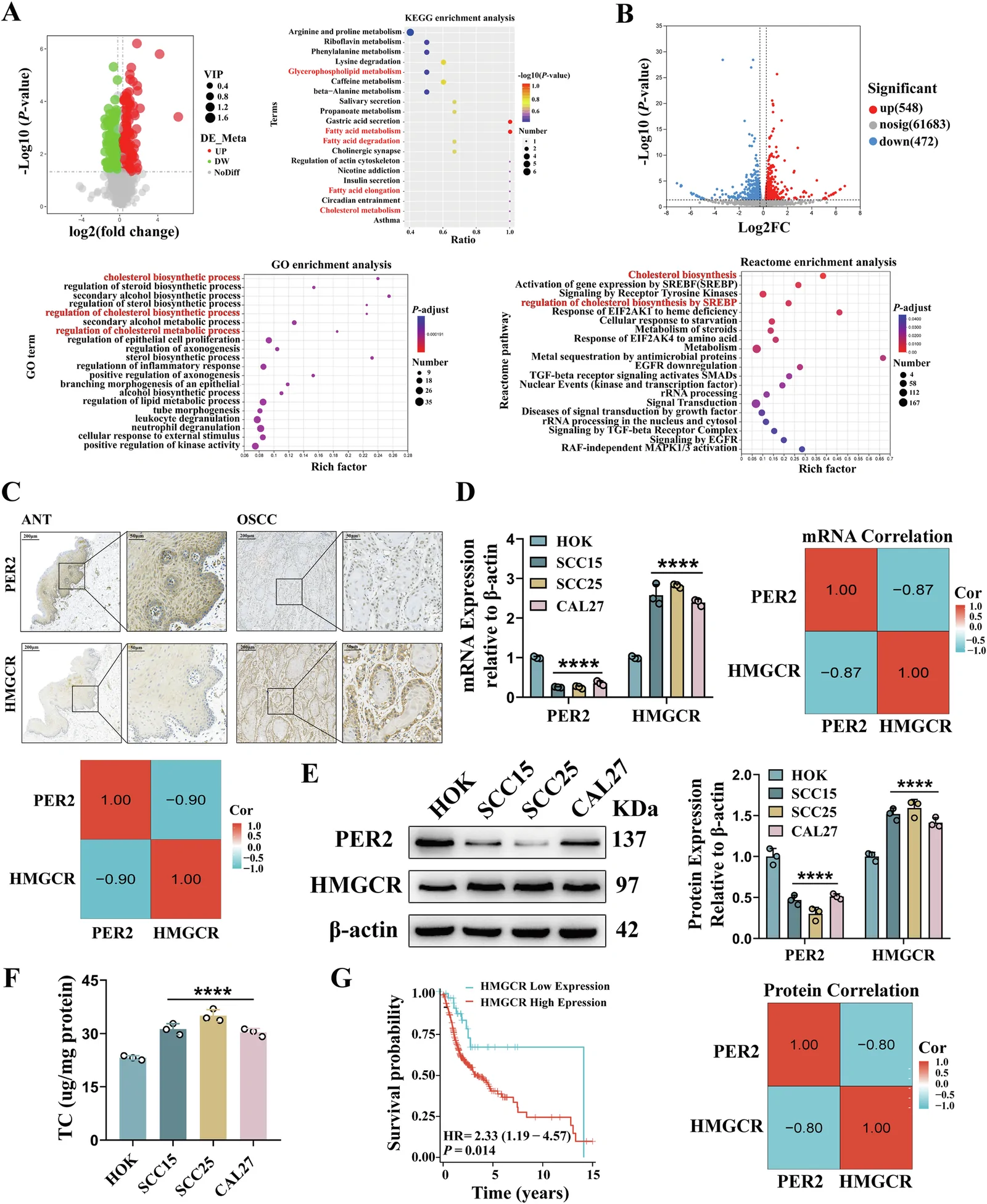
Alterations and correlations of PER2 and HMGCR expression in OSCC tissues and cells. **A** Untargeted metabolomics analysis showing volcano plot of differential metabolites and their KEGG enrichment analysis (*n* = 6 per group). **B** Transcriptome sequencing results showing volcano plot of differentially expressed genes and their GO and Reactome enrichment analysis (*n* = 3 per group). **C** IHC detection of PER2 and HMGCR expression in tumor tissues and paired adjacent normal tissues from OSCC patients (*n* = 104 per group, scale bars = 50 μm), followed by Spearman correlation analysis. **D** RT-qPCR detection of *PER2* and *HMGCR* mRNA expression in normal oral keratinocytes (HOK) and three OSCC cell lines (SCC15, SCC25 and CAL27), followed by Spearman correlation analysis. **E** Western blotting detection of PER2 and HMGCR protein expression in HOK, SCC15, SCC25 and CAL27 cells, followed by Spearman correlation analysis. **F** Cholesterol assay showed significantly increased TC levels in three OSCC cell lines compared to HOK cells. **G** Kaplan–Meier analysis of TCGA dataset demonstrated significantly reduced survival rates in OSCC patients with high HMGCR expression. All data represent three independent experiments. Data are presented as means ± SD (*n* ≥ 3). *P < 0.05; **P < 0.01; ***P < 0.001; ****P < 0.0001. IHC Immunohistochemical, OSCC oral squamous cell carcinoma, TC total cholesterol, ANT adjacent normal tissue.

Prior research indicates that decreased PER2 levels markedly reduces survival time in OSCC patients [20, 23]. We established two stable *PER2* overexpression OSCC cell lines, namely OE-PER2-SCC15 and OE-PER2-SCC25. We also obtained three stable *PER2*-silenced OSCC cell lines with different effector targets, namely sh-PER2-CAL27#1, sh-PER2-CAL27#2, and sh-PER2-CAL27#3 (Fig. S1A–D). Untargeted metabolomics analysis of normal control (NC-SCC25) and OE-PER2-SCC25 cells identified 273 differential metabolites (Table S1). According to KEGG enrichment analysis, five of the top 20 enriched pathways in these cells were associated with cholesterol and lipid metabolism (Fig. 1A and Table S2). Subsequently, we performed second-generation transcriptome sequencing (RNA-seq) of sh-PER2-CAL27#2 cells, which exhibited the highest *PER2* knockdown efficiency, and identified 1020 differentially expressed genes (Table S3). In GO and reactome enrichment analyses of these genes, the top-ranking pathway was “cholesterol biosynthesis process” (Fig. 1B and Tables S4 and S5). These results demonstrate a close association between *PER2* and cholesterol synthesis. The cholesterol-synthesis-related genes identified in the enrichment analysis included the gene encoding HMGCR (Tables S4 and S5) [5, 9]. Thus, we proposed that *PER2* may modulate cholesterol synthesis in OSCC cells via HMGCR.

To test our hypothesis, we performed immunohistochemical (IHC) detection of PER2 and HMGCR expression levels and their correlations in OSCC tumor tissues and paired adjacent normal tissues. Compared to adjacent normal tissues, the expression of PER2 was significantly downregulated in OSCC, while the expression of HMGCR was markedly upregulated. There was a significant negative correlation between the expression levels of the two (Fig. 1C). Through RT-qPCR and western blotting assays on three OSCC cell lines (SCC15, SCC25, and CAL27), it was found that compared with normal oral keratinocytes (HOK), the mRNA and protein levels of PER2 were significantly reduced, while the levels of HMGCR were significantly increased, and there was a significant negative correlation between the expression levels of PER2 and HMGCR (Fig. 1D, E). Cholesterol assays showed significantly increased total cholesterol (TC) in the three OSCC cell lines compared to HOK cells (Fig. 1F). Further Kaplan–Meier analysis of the TCGA dataset revealed that OSCC patients with high HMGCR expression demonstrated markedly lower survival rates (Fig. 1G and Table S6). The analysis reveals that *PER2* may inhibit OSCC progression by suppressing HMGCR and reducing intracellular cholesterol synthesis.

### *PER2* regulates cholesterol synthesis and proliferation in OSCC cells through HMGCR-dependent mechanisms in vitro and in vivo

**Fig. 2:**
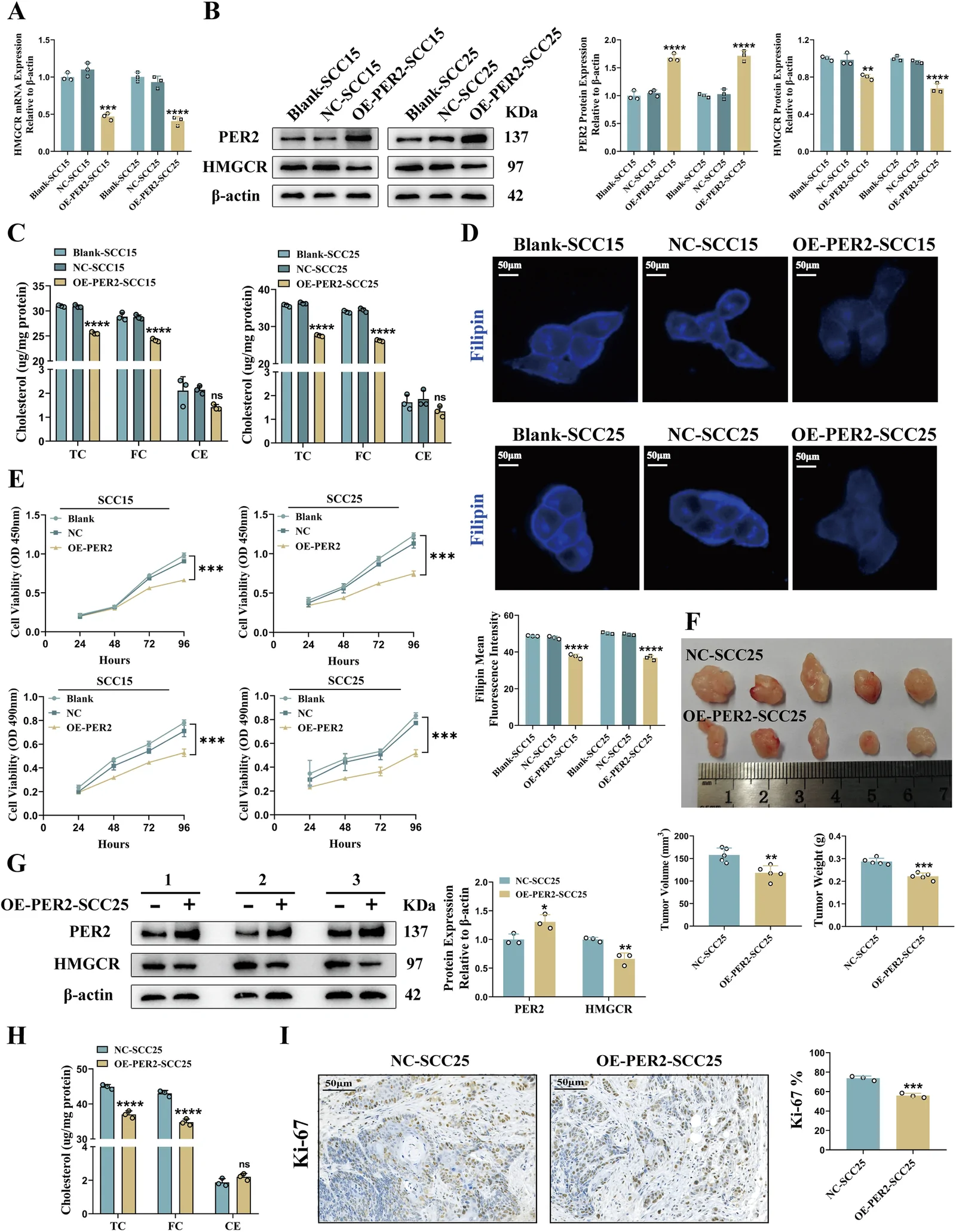
PER2 regulates intracellular cholesterol and proliferation in OSCC through HMGCR-dependent mechanisms. **A** RT-qPCR analysis demonstrated significantly decreased *HMGCR* mRNA expression levels in both OE-PER2-SCC15 and OE-PER2-SCC25 cells. **B** Western blotting confirmed markedly reduced HMGCR protein expression levels in both PER2-overexpressing SCC15 and SCC25 cells. **C** Cholesterol assays revealed significant reductions in TC and FC, but no significant change in CE, in OE-PER2-SCC15 and OE-PER2-SCC25 cells. **D** Filipin staining showed significantly decreased FC levels in PER2-overexpressing OSCC cells. **E** Both CCK-8 and MTT assays indicated significantly inhibited proliferation in OE-PER2-SCC15 and OE-PER2-SCC25 cells compared to controls. **F** NC-SCC25 and OE-PER2-SCC25 cells were subcutaneously injected into nude mice to establish xenograft tumors, with subsequent measurement of tumor volume and weight upon harvest (*n* = 5 animals per group). **G** Western blotting analysis demonstrated significantly increased PER2 and decreased HMGCR protein expression in OE-PER2-SCC25 xenografts compared to NC-SCC25 controls. **H** Cholesterol assays revealed significant reductions in TC and FC in OE-PER2-SCC25 tumors, with no significant changes in CE. **I** IHC detection of Ki-67% changes in NC-SCC25 and OE-PER2-SCC25 tumor tissues. All data represent three independent experiments. Data are presented as means ± SD (*n* ≥ 3). *P < 0.05; **P < 0.01; ***P < 0.001; ****P < 0.0001. FC free cholesterol, CE cholesteryl esters.

Further explore the impact of *PER2* expression alterations on cholesterol synthesis and cell proliferation in OSCC cells. Intracellular TC comprises free cholesterol (FC) and cholesteryl ester (CE) [24]. Multiple experimental approaches consistently showed that compared with control groups, both HMGCR mRNA and protein expression levels, along with TC and FC contents, were significantly decreased in OE-PER2-SCC15 and OE-PER2-SCC25 cells, whereas CE showed no significant difference (Fig. 2A–C). Filipin staining confirmed that intracellular FC were significantly reduced in both PER2-overexpressing OSCC cell lines (Fig. 2D), and CCK-8 and MTT assays demonstrated that cell proliferation was significantly inhibited compared with the control group (Fig. 2E). However, in sh-PER2-CAL27#1, sh-PER2-CAL27#2, and sh-PER2-CAL27#3 cells, the opposite effects were observed, with CE still displaying no significant differences (Fig. S2A–E). Transfection of sh-PER2#2 lentivirus in OE-PER2-SCC25 cells significantly rescued the effects of *PER2* overexpression (Fig. S2F–I). To further verify that inhibition of cholesterol synthesis and cell proliferation by PER2 in OSCC cells is HMGCR dependent, we treated sh-PER2-CAL27#2 cells with HMGCR inhibitor simvastatin and found that cell proliferation and intracellular TC and FC levels were significantly restored (Fig. S2J–M).

Subsequently, we performed in vivo validation using a nude mouse subcutaneous xenograft tumor model. Significant reductions in both tumor volume and weight were observed in the OE-PER2-SCC25 group relative to NC-SCC25 controls (Fig. 2F). Moreover, tumors in the OE-PER2-SCC25 group showed markedly increased PER2 expression and significant decreases in HMGCR expression, TC, FC, and percentage of Ki-67-positive (Ki-67%) cells; again, there was no significant change in CE (Fig. 2G–I). Taken together, these results demonstrate that *PER2* suppresses FC synthesis and cell proliferation in OSCC cells by inhibiting HMGCR, while exhibiting no regulatory effect on CE.

### PER2 reduces cholesterol and proliferation in OSCC cells by binding to MYH9 and suppressing HMGCR

**Fig. 3:**
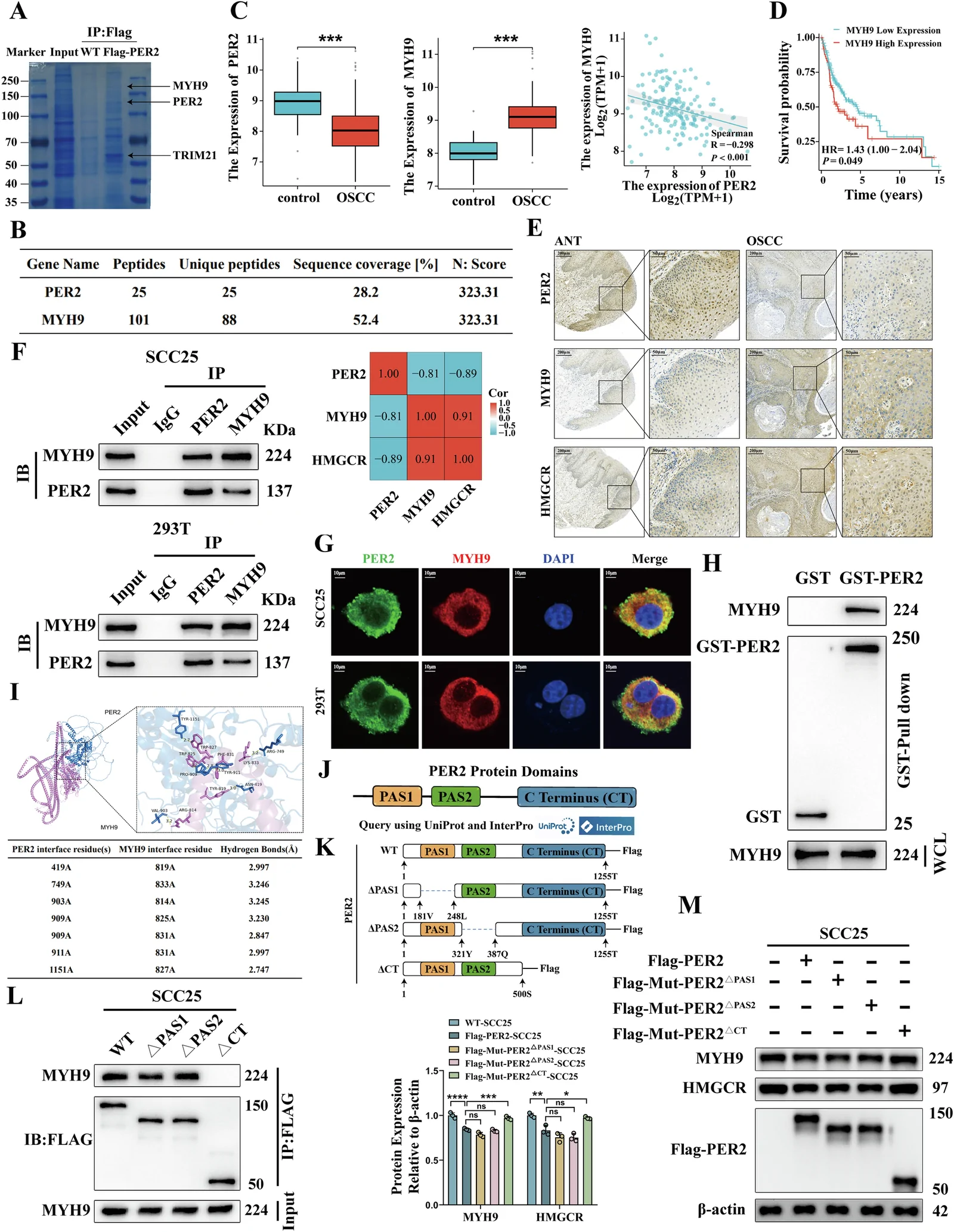
PER2 interaction with MYH9 suppresses HMGCR, reducing cholesterol synthesis and proliferation in OSCC cells. **A** Coomassie blue staining of IP-MS samples revealed prominent bands containing MYH9, PER2, and TRIM21, which were subsequently confirmed by mass spectrometry. **B** The mass spectrometry results for MYH9 in the IP-MS analysis. **C** Bioinformatics analysis of the GSE30784 dataset revealed significantly decreased PER2 and increased MYH9 expression in OSCC, respectively, along with a significant negative correlation between their expression levels. **D** Kaplan–Meier analysis of TCGA dataset demonstrated significantly reduced survival rates in OSCC patients with high MYH9 expression. **E** IHC detection of PER2, MYH9, and HMGCR expression in tumor tissues and paired adjacent normal tissues from OSCC patients (*n* = 104 per group, scale bars = 50 μm), followed by Spearman correlation analysis. **F** Co-IP assays confirmed PER2–MYH9 complex formation in both SCC25 and 293 T cells. **G**. Immunofluorescence co-localization analysis revealed cytoplasmic co-localization of PER2 and MYH9 proteins in both SCC25 and 293 T cells. **H** GST pull-down assays demonstrated direct binding between purified PER2 and MYH9 proteins in vitro, confirming formation of the PER2–MYH9 complex; **I**. Molecular docking results suggest that the binding sites between PER2 and MYH9 localizes to the C-terminal domain of PER2, **J** Schematic diagram shows PER2 protein interaction domains (PAS1, PAS2 and CT domains) based on UniProt and InterPro annotations. **K** Three Flag-tagged PER2 deletion mutant plasmids respectively lacking the PAS1, PAS2, and C-terminal domains were constructed (Flag-Mut-PER2^ΔPAS1^, Flag-Mut-PER2^ΔPAS2^, and Flag-Mut-PER2^ΔCT^); **L**. Co-IP assays demonstrated that no PER2–MYH9 complex was detected in Flag-Mut-PER2^ΔCT^-SCC25 cells. **M** Western blotting analysis showed that compared to Flag-PER2-SCC25 cells, MYH9 and HMGCR expression levels showed no significant changes in Flag-Mut-PER2^ΔPAS1^-SCC25 and Flag-Mut-PER2^ΔPAS2^-SCC25 cells, whereas their expression was significantly increased in Flag-Mut-PER2^ΔCT^-SCC25 cells. All data represent three independent experiments. Data are presented as means ± SD (*n* ≥ 3). *P < 0.05; **P < 0.01; ***P < 0.001; ****P < 0.0001.

We further investigated the mechanism by which PER2 regulates cholesterol synthesis in OSCC cells. Considering that PER2 is a member of the PAS domain family and its functional regulation mainly relies on protein–protein interactions [25], we first conducted proteomic analysis. IP-MS identified MYH9 as an abundant protein with high binding affinity for PER2 (Fig. 3A, B and Table S7). Subsequent bioinformatics analysis of the GSE30784 dataset showed that in OSCC, the expression levels of PER2 and MYH9 were significantly decreased and increased, respectively, with a significant negative correlation between the two (Fig. 3C and Table S8). Kaplan–Meier analysis of the TCGA dataset revealed that survival rates were significantly reduced in OSCC patients with high MYH9 expression (Fig. 3D and Table S9). Therefore, we hypothesized that PER2 could inhibit OSCC progression by binding to MYH9 and thereby reducing cholesterol synthesis in OSCC cells. Further IHC analysis of paired OSCC clinical samples showed that in OSCC, PER2 expression was significantly decreased, whereas MYH9 and HMGCR expression were significantly increased (Fig. 3E). PER2 expression showed significant negative correlations with both MYH9 and HMGCR expression, whereas MYH9 and HMGCR expression levels were significantly positively correlated (Fig. 3E). Western blotting demonstrated that MYH9 expression was markedly decreased in OE-PER2-SCC25 cells, conversely it was markedly increased in sh-PER2-CAL27#2 cells (Fig. S3A). However, treatment of sh-PER2-CAL27#2 cells with MYH9 inhibitor blebbistatin (HY-13813, MCE, USA) significantly rescued HMGCR expression, TC, FC, and cell proliferation (Fig. S3B–E).

The mRNA and protein expression of HMGCR were markedly increased in OE-MYH9-CAL27 cells (Fig. S3F, G). Moreover, treatment with the HMGCR inhibitor simvastatin significantly rescued TC, FC, and cell proliferation in OE-MYH9-CAL27 cells (Fig. S3H–K). Collectively, these results indicate that PER2 binds to and suppresses MYH9, thereby inhibiting HMGCR and ultimately reducing cholesterol synthesis and cell proliferation in OSCC cells.

We used Co-IP to investigate the interaction between PER2 and MYH9; in both SCC25 and 293 T cells, the two proteins bound to form a PER2–MYH9 complex (Fig. 3F). Immunofluorescence co-localization analysis demonstrated that in both cell types, PER2 and MYH9 proteins were co-localized in the cytoplasm (Fig. 3G), and GST pull-down assays confirmed that PER2 directly bound to MYH9 and formed a complex in vitro (Fig. 3H). Protein–protein docking prediction analysis showed a strong binding interaction (docking score: −247.12), with PER2 binding to MYH9 through its C-terminal domain (Fig. 3I). Comprehensive analysis of PER2 using the UniProt and InterPro databases identified three primary protein-interaction domains: PAS1, PAS2, and the C-terminal domain (Fig. 3J). We thus constructed three Flag-tagged PER2 deletion mutants lacking these three domains (Flag-Mut-PER2^ΔPAS1^-SCC25, Flag-Mut-PER2^ΔPAS2^-SCC25, and Flag-Mut-PER2^ΔCT^-SCC25) (Fig. 3K). Co-IP assays demonstrated the presence of PER2–MYH9 complexes in Flag-Mut-PER2^ΔΔPAS1^-SCC25 and Flag-Mut-PER2^ΔPAS2^-SCC25 cells, but the formation of this complex was not observed in Flag-Mut-PER2^ΔCT^-SCC25 cells (Fig. 3L). Western blotting analysis showed that compared with Flag-PER2-SCC25 cells, the expression levels of MYH9 and HMGCR did not change significantly in Flag-Mut-PER2^Δ PAS1^-SCC25 and Flag-Mut-PER2^ΔPAS2^-SCC25 cells, whereas they were significantly upregulated in Flag-Mut-PER2^ΔCT^-SCC25 cells (Fig. 3M). These findings demonstrate that PER2 binds to MYH9 through its C-terminal domain to form a PER2–MYH9 complex, thereby inhibiting the expression of both MYH9 and HMGCR.

### PER2 recruits TRIM21 to promote ubiquitin-dependent degradation of MYH9

**Fig. 4:**
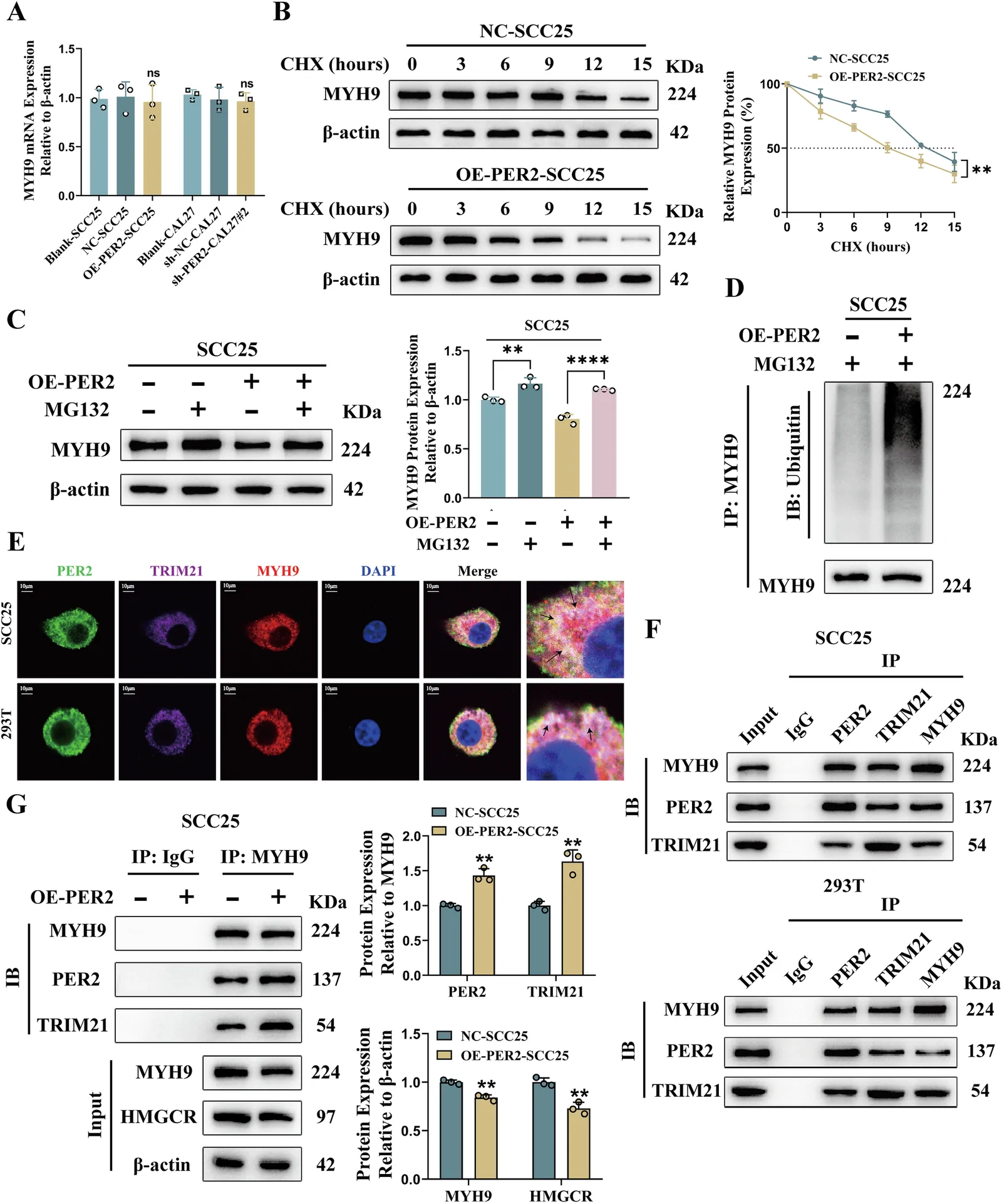
PER2 recruits TRIM21 to promote ubiquitin-dependent degradation of MYH9 protein. **A** RT-qPCR analysis showed no significant changes in *MYH9* mRNA levels upon either PER2-overexpressing or PER2-silenced OSCC cells. **B** CHX chase assay measured MYH9 protein half-life in NC-SCC25 and OE-PER2-SCC25 cells. **C** Western blotting detected MYH9 protein expression in OE-PER2-SCC25 cells treated with the proteasome inhibitor MG132. **D** The ubiquitination assay results showed that the ubiquitination level of MYH9 was significantly enhanced in OE-PER2-SCC25 cells. **E** Immunofluorescence co-localization analysis revealed cytoplasmic co-localization of PER2, TRIM21 and MYH9 proteins in both SCC25 and 293 T cells. **F** Co-IP results showed the presence of PER2–TRIM21–MYH9 protein complexes in both SCC25 and 293 T cells. **G** Co-IP coupled with Western blotting showing increased PER2–TRIM21 complex formation in OE-PER2-SCC25 cells compared to NC-SCC25 controls. All data represent three independent experiments. Data are presented as means ± SD (*n* ≥ 3). *P < 0.05; **P < 0.01; ***P < 0.001; ****P < 0.0001.

To further explore the mechanism by which PER2 inhibits MYH9, RT-qPCR assays in OE-PER2-SCC25 and sh-PER2-CAL27#2 cells showed no significant alterations in *MYH9* mRNA expression levels (Fig. 4A), suggesting that the regulation of MYH9 by PER2 does not occur at the transcriptional level. By contrast, cycloheximide (CHX) chase assays showed that the half-life of MYH9 protein in OE-PER2-SCC25 cells was significantly shortened compared to NC-SCC25 cells (Fig. 4B), with markedly reduced MYH9 protein levels, effects that were significantly reversed by addition of proteasome inhibitor MG132 (Fig. 4C). Moreover, ubiquitination of MYH9 was significantly enhanced in OE-PER2-SCC25 cells (Fig. 4D), indicating that PER2 promotes ubiquitin-dependent degradation of MYH9 through the ubiquitin-proteasome pathway.

As PER2 is not a member of the ubiquitin-proteasome family, we speculated that it could exert its effects via regulation of ubiquitin ligases or deubiquitinating enzymes. Given that our IP-MS data revealed that the E3 ubiquitin ligase TRIM21 was highly enriched in the Flag-IP samples (Fig. 3A and Table S7), we further hypothesized that the mechanism involved recruitment of TRIM21 by PER2. Indeed, immunofluorescence analysis revealed cytoplasmic co-localization of PER2, TRIM21, and MYH9 in SCC25 and 293 T cells (Fig. 4E), and Co-IP showed the presence of PER2–TRIM21–MYH9 protein complexes in both cell types (Fig. 4F). Compared with NC-SCC25 cells, the abundance of PER2–TRIM21 complex was significantly higher in OE-PER2-SCC25 cells (Fig. 4G), whereas RT-qPCR and western blotting analyses indicated no significant changes in total intracellular mRNA and protein expression levels of TRIM21 (Fig. S4A, B). Subsequent transfection of OE-PER2-SCC25 cells with a short interfering RNA targeting *TRIM21* significantly rescued both MYH9 expression and ubiquitination levels (Fig. S4C, D), indicating that PER2 promotes ubiquitin-dependent degradation of MYH9 protein by recruiting TRIM21 as hypothesized. To validate these findings, we performed rescue experiments. MYH9 and HMGCR expression levels, MYH9 ubiquitination status, intracellular TC and FC, and cell proliferation levels were significantly rescued in Flag-PER2-SCC25 cells containing PER2 with a mutated C-terminal domain compared with unmutated control cells (Fig. S4E–I). Collectively, these results demonstrate that PER2 depends on binding to MYH9 and recruitment of TRIM21 to promote ubiquitin-dependent degradation of MYH9, thereby suppressing HMGCR expression and consequently reducing intracellular cholesterol synthesis and proliferation in OSCC cells.

### Simvastatin combined with PER2 overexpression enhances the therapeutic efficacy in treating OSCC

**Fig. 5:**
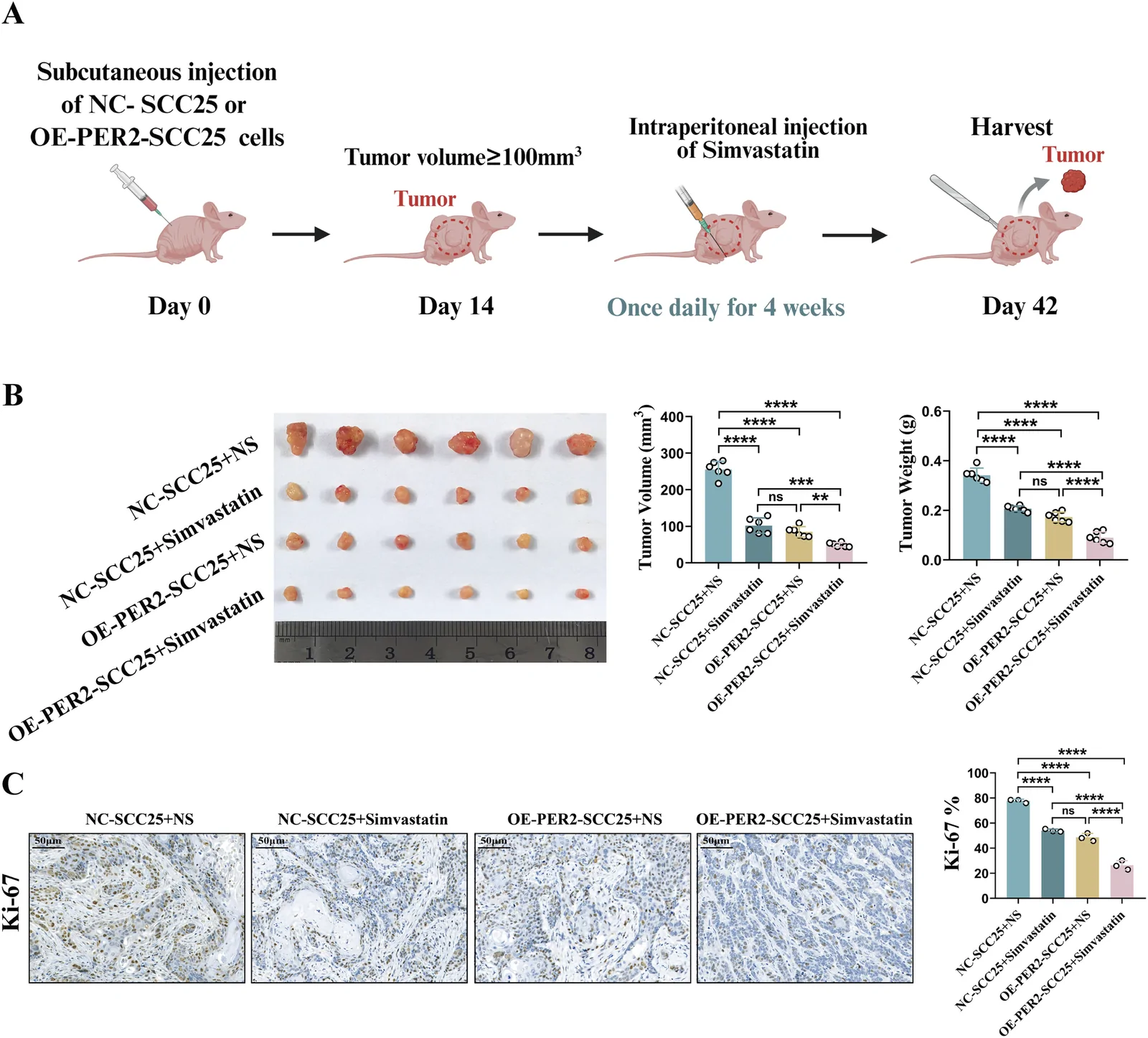
Simvastatin combined with PER2 overexpression enhances the therapeutic efficacy in treating OSCC. **A** Schematic diagram of Male BALB/c nude mice were subcutaneously injected with NC-SCC25 or OE-PER2-SCC25 cells to establish OSCC xenograft models, followed by treatment with simvastatin. **B** Tumors were harvested at day 42 followed by measurement of volume and weight (*n* = 6 animals per group). **C** IHC detection of changes in Ki-67% in tumor tissues from the NC-SCC25 + NS, NC-SCC25+Simvastatin, OE-PER2-SCC25 + NS and OE-PER2-SCC25+Simvastatin groups. All data represent three independent experiments. Data are presented as means ± SD (*n* ≥ 3). *P < 0.05; **P < 0.01; ***P < 0.001; ****P < 0.0001. NS Normal Saline.

Simvastatin is an HMGCR inhibitor and an FDA-approved cholesterol-lowering medication [9]. We established a subcutaneous OSCC xenograft model in nude mice to evaluate the therapeutic efficacy of simvastatin alone or in combination with PER2 overexpression in treating OSCC (Fig. 5A). The results showed that compared with the NC-SCC25 + NS group, both the NC-SCC25+Simvastatin and OE-PER2-SCC25 + NS groups exhibited significantly reduced tumor volume, weight, and tumor cell Ki-67% (Fig. 5B, C). However, there were no significant differences in these parameters between the NC-SCC25+Simvastatin and OE-PER2-SCC25 + NS groups (Fig. 5B, C). In contrast, the tumor volume, weight, and tumor cell Ki-67% in the OE-PER2-SCC25+Simvastatin group were significantly lower than those in the NC-SCC25+Simvastatin or OE-PER2-SCC25 + NS group, respectively (Fig. 5B, C). These findings indicate that either simvastatin or PER2 overexpression can significantly inhibit OSCC growth, but the combined anti-cancer effect is significantly stronger than either treatment alone.

### PER2 regulates the circadian rhythms of HMGCR expression, cholesterol, and cell proliferation in OSCC cells

**Fig. 6:**
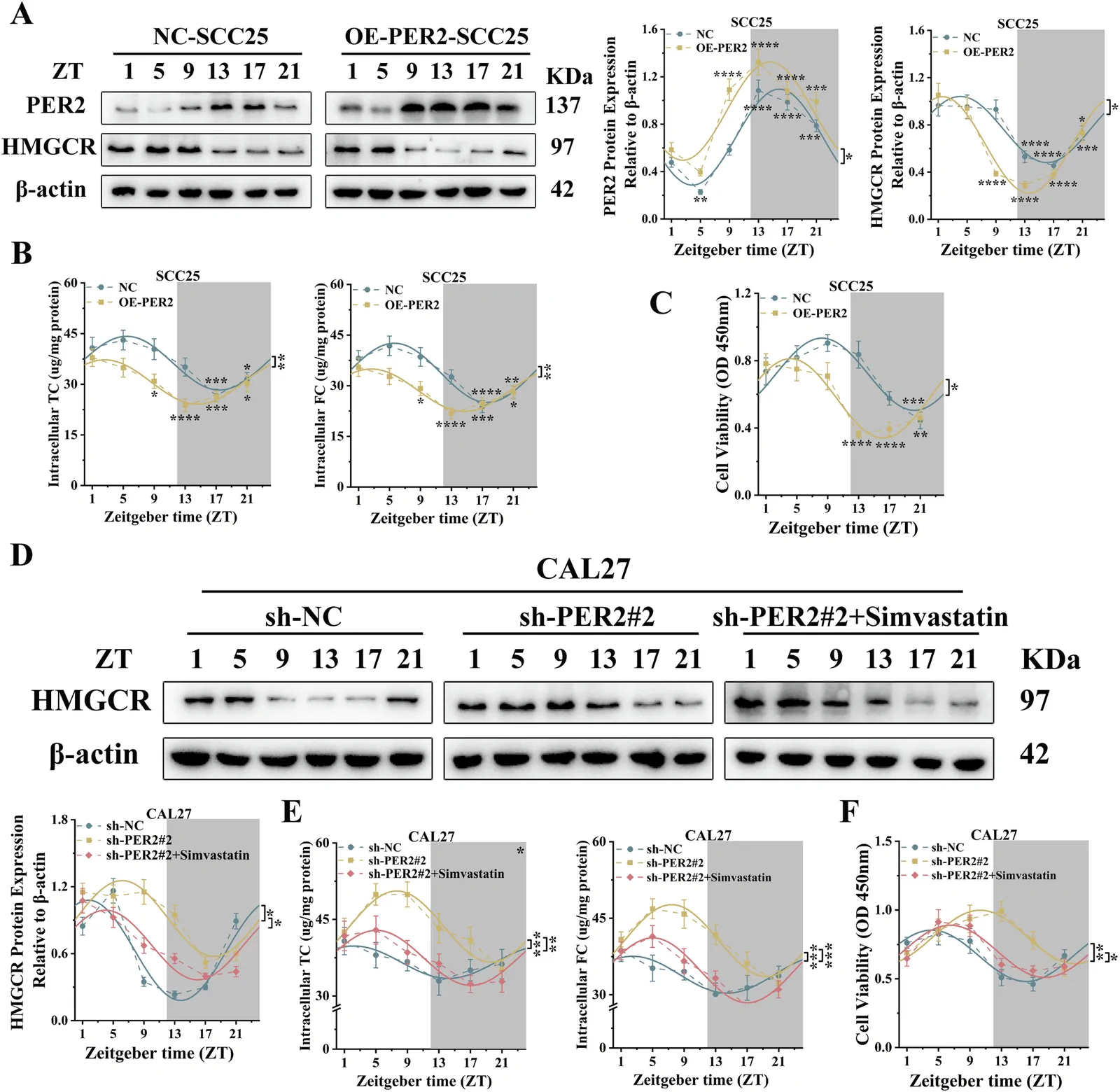
PER2 regulates the circadian rhythms of HMGCR, cholesterol and proliferation in OSCC cells in vitro*.* Circadian rhythms of PER2 and HMGCR protein expression (**A**), intracellular TC and FC (**B**), and cell viability (**C**) in NC-SCC25 and OE-PER2-SCC25 cells, measured at six different time points within 24 h. Circadian rhythms of HMGCR protein expression (**D**), intracellular TC and FC (**E**), and cell viability (**F**) in sh-NC-CAL27, sh-PER2-CAL27#2, and sh-PER2-CAL27#2+Simvastatin cells, measured at six different time points within 24 h. All data were subjected to cosine curve fitting and rhythm analysis (one-way ANOVA: *P* < 0.05; cosinor analysis: *P* < 0.05). In all figures, solid lines represent cosine-fitted curves while dashed lines connect actual mean values at six different time points within 24 h. All data represent three independent experiments. Data are shown as the mean ± SD (*n* ≥ 3). *P < 0.05; **P < 0.01; ***P < 0.001; ****P < 0.0001.

As previously demonstrated that PER2 expression is significantly downregulated in OSCC, showing a significant negative correlation with HMGCR expression and intracellular cholesterol. Additionally, PER2 regulates intracellular cholesterol and cell proliferation in OSCC cells in a HMGCR-dependent manner. Given that the expression of the circadian clock gene *PER2* exhibits circadian rhythmicity [18]. Mesor, amplitude and acrophase are three indicators reflecting the characteristics of 24-h circadian rhythm [26]. We further investigate whether *PER2* regulates the circadian rhythms of HMGCR expression, intracellular cholesterol and cell proliferation in OSCC cells. The results showed that PER2 and HMGCR expression, intracellular TC and FC, and cell proliferation exhibited circadian rhythms in both NC-PER2-SCC25 and OE-PER2-SCC25 cells (Fig. 6A–C and Table S10). However, the circadian pattern of PER2 expression showed an inverse relationship with the rhythms of HMGCR expression, cholesterol levels, and cell proliferation (Fig. 6A–C and Table S10), suggesting that PER2 regulates the circadian rhythms of HMGCR expression, cholesterol, and cell proliferation. Further analysis showed that compared with NC-PER2-SCC25 cells, OE-PER2-SCC25 cells exhibited a significantly increased mesor of PER2 expression rhythm, while the mesors of HMGCR expression, intracellular TC and FC, and cell proliferation were significantly decreased (Fig. 6A–C and Table S10). Additionally, the acrophases of the circadian rhythms of PER2 and HMGCR expression, intracellular TC and FC and cell proliferation were all consistently advanced, with no significant differences in amplitude (Fig. 6A–C and Table S10). In contrast, sh-PER2-CAL27#2 cells showed opposite changes in the mesors and acrophases of the circadian rhythms of HMGCR expression, intracellular TC and FC, and cell proliferation (Fig. 6D–F and Table S10). Following the addition of the HMGCR inhibitor simvastatin to sh-PER2-CAL27#2 cells, the mesors and delayed acrophases of HMGCR expression, intracellular TC and FC, and cell proliferation were significantly rescued (Fig. 6D–F and Table S10). Collectively, these results indicate that in OSCC cells, PER2 regulates the circadian rhythms of HMGCR expression, intracellular cholesterol, and cell proliferation through two dimensions: mesor and acrophase.

### Circadian characteristics and sex differences in PER2 and HMGCR expression, intracellular cholesterol, cell proliferation and tumor growth in vivo

**Fig. 7:**
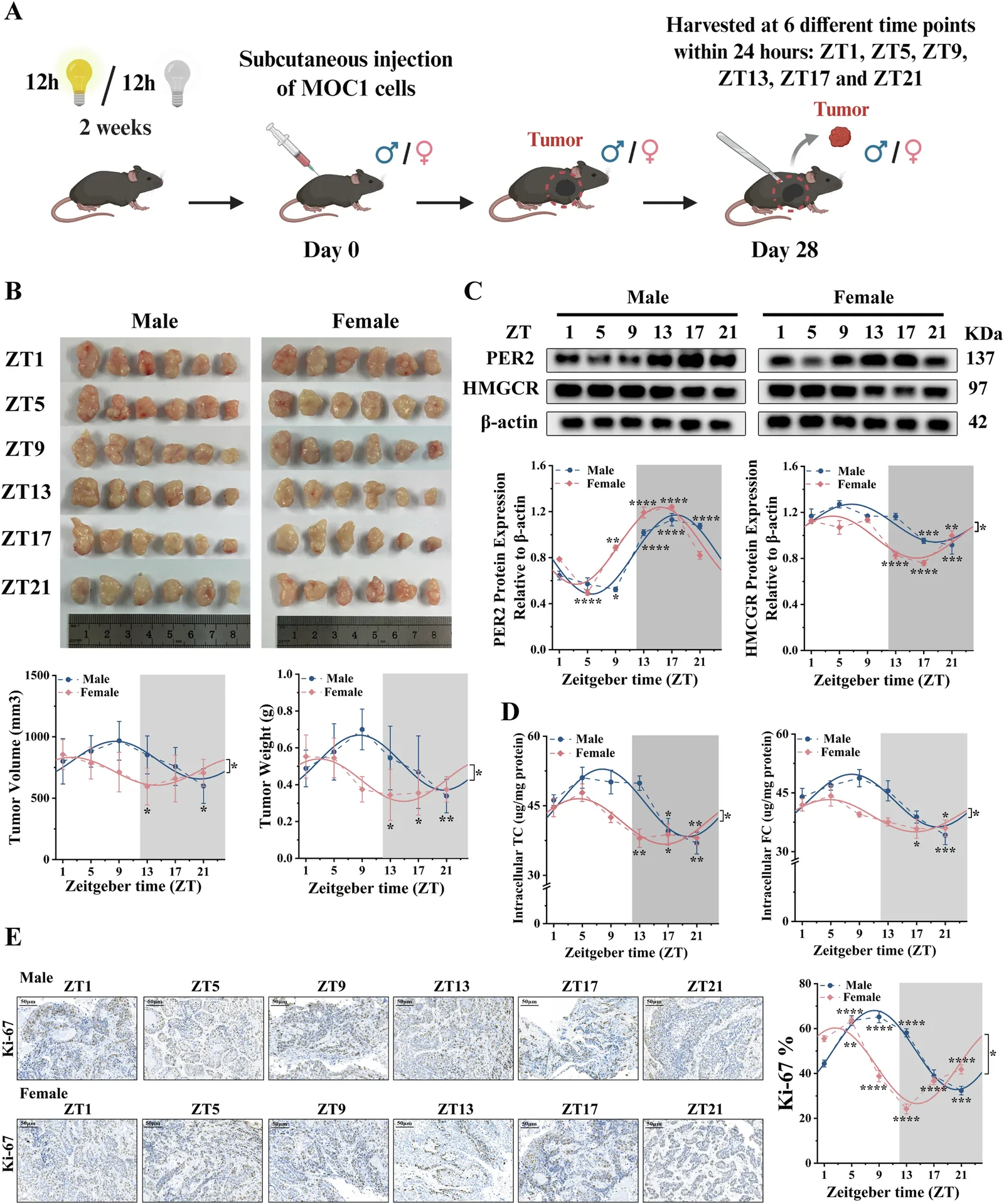
Circadian characteristics and sex differences in PER2 and HMGCR expression, intracellular cholesterol, cell proliferation and tumor growth in vivo*.* **A** Schematic diagram of establishing OSCC models by subcutaneous injection of MOC1 OSCC cells into male and female C57BL/6 J mice, with tumors harvested at six different time points (ZT1, ZT5, ZT9, ZT13, ZT17, ZT21) within 24 h on day 28. **B** Following tumor harvest at six different time points within 24 h on day 28, tumor volumes and weights were measured at each time point and subjected to cosine curve fitting and analysis (one-way ANOVA: *P* < 0.05; cosinor analysis: *P* < 0.05) (*n* = 6 animals per group). Circadian rhythms of PER2 and HMGCR protein expression (**C**), intracellular TC and FC (**D**), and Ki-67% changes (**E**) in OSCC tissues of male and female mice, measured at six different time points within 24 h, followed by cosine curve fitting and analysis (one-way ANOVA: *P* < 0.05; cosinor analysis: *P* < 0.05). In all figures, solid lines represent cosine-fitted curves while dashed lines connect actual mean values at six different time points within 24 h. All data represent three independent experiments. Data are presented as means ± SD (*n* ≥ 3). *P < 0.05; **P < 0.01; ***P < 0.001; ****P < 0.0001.

We established subcutaneous OSCC xenograft models in male and female C57BL/6 J mice (Fig. 7A, B) to further explore the circadian characteristics and sex differences in PER2 and HMGCR expression, intracellular cholesterol, cell proliferation, and tumor growth in OSCC in vivo through three dimensions: mesor, amplitude, and acrophase. The results showed that PER2 and HMGCR expression, intracellular TC and FC, cell proliferation, and tumor growth all exhibited circadian rhythms in OSCC of male and female mice (Fig. 7B–E and Table S11), consistent with the trends observed in vitro cell studies. The circadian pattern of PER2 expression in OSCC of male and female mice was opposite to those of HMGCR expression, intracellular TC and FC, cell proliferation, and tumor growth (Fig. 7B–E and Table S11), suggesting that PER2 regulates the circadian rhythms of HMGCR expression, cholesterol metabolism, cell proliferation, and tumor growth.

Further analysis of sex differences revealed that the acrophase of PER2 expression rhythm in OSCC of male mice was delayed by 2.18 h compared to females (ZT17.58 vs. ZT15.40), with no significant differences in mesor or amplitude. The mesor of HMGCR expression rhythm in OSCC of male mice was significantly higher than in females, and its acrophase was delayed by 2.70 h (ZT6.71 vs. ZT4.01), while the amplitude showed no significant sex difference (Fig. 7C and Table S11). In OSCC of male mice, the mesors of intracellular TC and FC circadian rhythms were significantly higher than in females, with acrophases delayed by 3.28 h (ZT7.79 vs. ZT4.51) and 3.16 h (ZT7.92 vs. ZT4.76), respectively, while no significant difference in amplitude between the two sexes (Fig. 7D and Table S11). The mesor of Ki-67% circadian rhythm in OSCC tissues of male mice was significantly higher than in females, and its acrophase was delayed by 5.71 h (ZT8.31 vs. ZT2.60), with no significant difference in amplitude (Fig. 7E and Table S11). Similarly, the mesors of tumor volume and weight in male mice were significantly higher than in females, with acrophases delayed by 6.18 h (ZT8.49 vs. ZT2.31) and 5.70 h (ZT8.60 vs. ZT2.90), respectively, though amplitude did not differ significantly between sexes (Fig. 7B and Table S12). We also demonstrated at the transcriptional level that both PER2 and HMGCR mRNA expression in OSCC of male and female mice exhibited circadian rhythms, with circadian characteristics consistent with those observed at the protein levels (Fig. S5A and Table S13). These results indicate that PER2 and HMGCR expression, intracellular TC and FC, cell proliferation, and tumor growth in OSCC of male and female mice all exhibit sex-specific circadian rhythms, primarily manifested in the two dimensions of mesor and acrophase.

### Time-dependence and sex differences in the therapeutic efficacy of simvastatin against OSCC

**Fig. 8:**
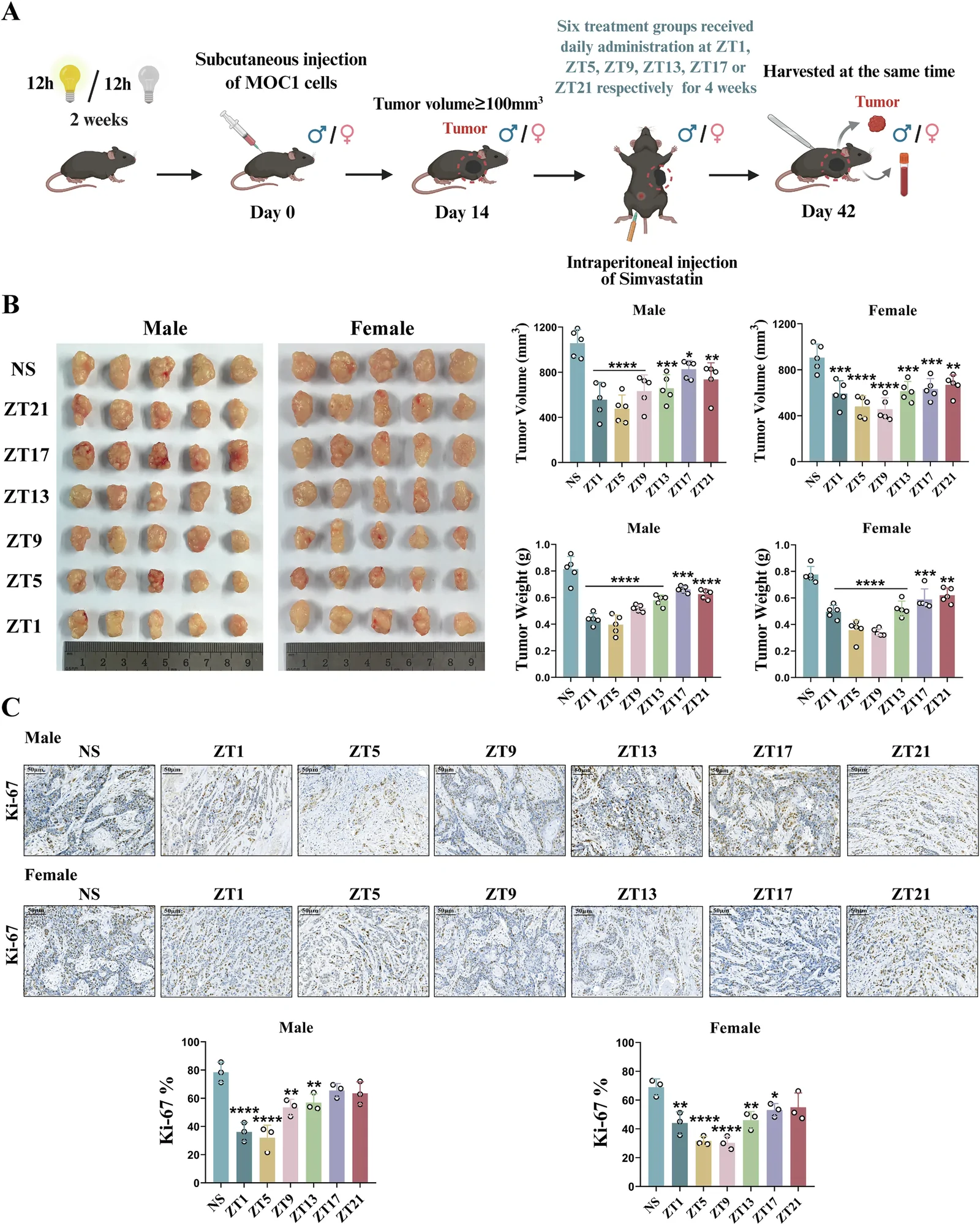
Time-dependence and sex differences in therapeutic efficacy of simvastatin against OSCC. **A** Schematic diagram of OSCC model establishment in male and female C57BL/6 J mice through subcutaneous injection of MOC1 cells, followed by simvastatin treatment at six different circadian time points (ZT1, ZT5, ZT9, ZT13, ZT17, ZT21) within 24 h, respectively. **B** Tumors were harvested at the same time point on day 42, followed by measurement of tumor volume and weight in each group (*n* = 5 animals per group). **C** IHC analysis of Ki-67% changes in tumor tissues from male and female mice across different treatment time groups. All data represent three independent experiments. Data are presented as means ± SD (*n* ≥ 3). Significant differences versus the NS group are indicated as *P < 0.05; **P < 0.01; ***P < 0.001; ****P < 0.0001.

To evaluate chronotherapeutic and sex differences in simvastatin efficacy, we established subcutaneous OSCC models in male and female C57BL/6 J mice (Fig. 8A). Simvastatin administered at six different time points within 24 h showed significant therapeutic efficacy against OSCC compared with the control group. However, the antitumor effects varied significantly depending on the timing of administration (Fig. 8B). Based on tumor weights, the optimal antitumor efficacy was achieved in male mice at ZT5 (51.35% tumor suppression rate), with the lowest efficacy at ZT17 (18.67% suppression rate). Notably, treatment at ZT5 showed 2.75-fold greater tumor suppression compared with ZT17. In female mice, optimal antitumor efficacy was achieved at ZT5 and ZT9 (tumor suppression rates of 53.87% and 55.93%, respectively), representing 2.68-fold and 2.78-fold increases compared with ZT21 (20.10% suppression, lowest efficacy) (Fig. 8B and Table S14). Tumor volume assessment yielded similar results (Fig. 8B and Table S14). IHC analysis also revealed that simvastatin treatment significantly decreased Ki-67% in tumor cells when administered at ZT5 in male mice and at ZT5 and ZT9 in female mice, compared to other time points (Fig. 8C and Table S14). These results demonstrate that while there were no significant differences between male and female mice in either the maximum or the minimum tumor suppression rates achieved with simvastatin, the optimal therapeutic time window for simvastatin treatment of OSCC in female mice was broader than that in male mice, and the time corresponding to the relative minimum therapeutic efficacy also exhibited sex differences.

We next measured serum levels of alanine aminotransferase (ALT), aspartate aminotransferase (AST), and creatine kinase (CK) in mice to evaluate hepatotoxicity and myotoxicity, the primary adverse effects of simvastatin [27, 28]. We observed no adverse effects in either male or female mice when the treatment was administered at ZT1, ZT5, or ZT21. However, hepatotoxicity (elevated aspartate aminotransferase levels) was observed in male mice treated at ZT9 and ZT13 and in female mice at ZT9, ZT13, and ZT17; there was also significantly greater hepatotoxicity in females compared to males (Fig. S6 and Table S14). Female mice treated with simvastatin at ZT13 developed myotoxicity (elevated creatine kinase levels), whereas no myotoxicity was observed in male mice at any administration time point (Fig. S6 and Table S14). These results demonstrate that the adverse effects of simvastatin in treating OSCC exhibit administration time-dependence and sex differences, with the time window for the occurrence of adverse reactions being broader in female mice than in male mice.

## Discussion

Extensive evidence demonstrates that cancer cells require a sustained cholesterol supply to maintain their aberrant proliferation. Elevated cholesterol levels have pivotal roles in both tumorigenesis and cancer progression, and dysregulated cholesterol homeostasis is considered to be a hallmark of cancer [5, 29]. Therapeutic strategies targeting dysregulated cholesterol metabolism reprogramming are considered a potential critical breakthrough in cancer therapy [6, 7, 30], yet exploring the underlying regulatory mechanisms remains a current challenge. With recent studies showing that hyperactivation of the PI3K/AKT, p53, and Wnt pathways in cancer cells promote cholesterol biosynthesis and uptake through regulation of sterol regulatory element-binding protein activity [5–7, 30]. In this study, we found that expression of the circadian clock gene PER2 was decreased in OSCC, which enhanced intracellular cholesterol synthesis and promoted OSCC cell proliferation. Furthermore, we integrated untargeted metabolomics, proteomics, and transcriptome sequencing with in vivo and in vitro experiments. These analyses revealed a previously unrecognized regulatory mechanism by which HMGCR, the key rate-limiting enzyme in cholesterol synthesis, is regulated by the core clock gene PER2. PER2 suppresses HMGCR expression and intracellular cholesterol synthesis by binding to MYH9 and recruiting the E3 ubiquitin ligase TRIM21 to promote ubiquitin-dependent degradation of MYH9, thereby inhibiting OSCC cell proliferation. Regarding the potential mechanism by which PER2/TRIM21 promotes MYH9 degradation, MYH9 is generally associated with the actin cytoskeleton. Its C-terminal tail/coiled-coil domain is involved in molecular assembly, cytoskeletal localization, and protein–protein interactions. Previous studies have demonstrated that TMEM120B and KIF23 bind to MYH9 via this region and modulate its ubiquitination level and protein stability [31, 32]. Therefore, we speculate that PER2/TRIM21-mediated MYH9 ubiquitination may alter the binding status of MYH9 to cytoskeleton-related proteins, thereby promoting its dissociation from the cytoskeletal network and subsequent proteasomal degradation. Furthermore, with respect to how MYH9 regulates HMGCR expression and cholesterol synthesis, accumulating evidence indicates that MYH9 participates in intracellular vesicle trafficking and transcytosis of low-density lipoprotein (LDL) [33, 34], and modulates the activity of lipid metabolism-related transcription factors such as SREBP [35]. SREBP2 acts as a critical transcriptional regulator of HMGCR, and its activation relies on ER-to-Golgi trafficking and nuclear translocation [36]. Therefore, we speculate that MYH9 may regulate the activation process of SREBP2, thereby affecting HMGCR expression and cholesterol synthesis. Consistent with the in vitro results, the OSCC mouse model also confirmed that PER2 overexpression significantly reduced intracellular cholesterol and inhibited OSCC growth. Simvastatin, a cholesterol-lowering drug approved by the FDA that inhibits HMGCR [9], is widely used to treat hypercholesterolemia and coronary artery disease [37]. In recent years, simvastatin has been found to exert favorable anti-cancer effects in various malignant tumors [37–39]. This study demonstrates that simvastatin exerts potent anti-cancer effects against OSCC by inhibiting HMGCR. Meanwhile, in an OSCC mouse model, it was confirmed that the combination of PER2 overexpression and the HMGCR inhibitor simvastatin significantly enhanced the anti-OSCC efficacy compared with either monotherapy. This study not only uncovers a novel mechanism of cholesterol metabolism regulation in cancer cells, but also provides new potential targets and combination therapeutic strategies for OSCC treatment.

Circadian rhythms are a fundamental characteristic of living organisms [40, 41]. The circadian rhythm (or biological clock) means that cellular physiological activities in organisms do not remain at constant levels throughout the day but instead exhibit rhythmic changes with 24-h periodicity [14, 40]. Circadian rhythms comprise three dimensions: mesor, amplitude, and acrophase. The mesor represents the average value of the 24-h oscillation, whereas the amplitude indicates the degree of oscillation above the mesor and is a key indicator of gene expression stability. The acrophase corresponds to the time point at which the rhythmic oscillation reaches its peak, reflecting the timing of maximal physiological activity within the 24-h cycle [18, 26]. Disruption in any dimension of circadian rhythms can lead to dysregulation of cellular physiological homeostasis and contribute to disease pathogenesis [13, 26]. It has been demonstrated that the circadian clock gene PER2 exhibits rhythmic oscillation in OSCC cells [18, 19]. Therefore, we further investigated the regulatory role of PER2 in the circadian rhythms of intracellular cholesterol and cell proliferation. Through silencing or overexpressing PER2 in OSCC cells, we found that PER2 regulates the circadian rhythms of HMGCR expression, intracellular cholesterol and cell proliferation, primarily in two dimensions: modulating the mesor and the acrophase.

We further explored the circadian rhythm characteristics of PER2 and HMGCR expression, intracellular TC and FC, and cell proliferation in OSCC in vivo. Given the substantial differences in genetics, hormone secretion, and behavioral activities between individuals of different sexes, circadian rhythms often exhibit sex differences [42]. In light of this, this study established OSCC models in both male and female mice respectively, and found that PER2 and HMGCR expression, intracellular TC and FC, and cancer cell proliferation all exhibited circadian rhythms in OSCC from both sexes. However, compared to female mice, the acrophases of PER2 and HMGCR expression, intracellular TC and FC, and cancer cell proliferation in OSCC of male mice were delayed by 2.18 h, 2.70 h, 3.28 h, 3.16 h, and 5.71 h, respectively, revealing progressively increasing delay in the acrophases along the PER2–HMGCR–cholesterol–cancer cell proliferation pathway. In addition to the delayed acrophases, the mesors of HMGCR expression, intracellular TC and FC, and cancer cell proliferation in OSCC of male mice were also significantly higher than those in females. This study is the first to clarify the sex differences in the circadian rhythms of PER2 and HMGCR expression, intracellular TC and FC, and cancer cell proliferation in OSCC between male and female mice.

Current evidence has demonstrated that circadian rhythms can exhibit differences of severalfold between peak and trough values [26, 43]. Since many metabolic enzymes or drug targets exhibit circadian rhythms, this temporal variation poses new requirements and challenges for cancer development and precision therapy. Current studies indicate that, compared to traditional fixed-time administration, administering drugs at specific restricted times (also known as chronotherapy) can enhance the efficacy of many drugs by more than two-fold, while improving drug tolerance by two- to ten-fold [44, 45]. Given that this study demonstrated that HMGCR exhibits circadian rhythms in OSCC, we established OSCC models in both male and female mice respectively. The mice were treated with simvastatin (an HMGCR inhibitor) at six different time points to explore the optimal therapeutic timing and sex differences. We found that simvastatin administration at each of the 6 different time points within 24 h achieved significant anti-tumor efficacy in both male and female mice. However, the antitumor efficacy of simvastatin varied substantially across these time points, with differences of up to 2.75-fold in male mice and 2.68-fold in female mice. Analysis of the timing for optimal efficacy revealed that the strongest antitumor effect in male mice occurred with treatment at ZT5, whereas female mice experienced equally optimal therapeutic effects at ZT5 and ZT9, indicating that female mice have a broader optimal therapeutic time window compared to males. Notably, the optimal therapeutic time window of simvastatin is not completely consistent with the acrophase of HMGCR protein expression, suggesting that its time-dependent efficacy is not simply determined by target protein abundance. Mice are nocturnal animals, with ZT5 falling within the resting phase, whereas ZT9 is close to the onset of activity and feeding. Cholesterol synthesis is regulated by the feeding-fasting cycle, and feeding-related signals can promote cholesterol synthesis via the mTORC1–USP20–HMGCR pathway [46, 47]. Therefore, the greater efficacy observed at ZT5 or ZT9 may reflect the combined effect of HMGCR rhythm and the cholesterol synthesis state before and after feeding. On the other hand, HMGCR protein abundance does not fully equate to its actual activity, and the efficacy of simvastatin may also be affected by drug transport, metabolism, and in vivo effective exposure duration [48–50]. The differences in the optimal administration windows between male and female mice may be attributed to sex-related differences in cholesterol metabolic responses and circadian rhythms of drug metabolism [51, 52]. Additionally, we observed sex differences in the timing of the relatively lowest therapeutic efficacy: the anti-tumor effect was relatively weakest when male mice were treated at ZT17, while the relatively lowest anti-tumor efficacy in female mice was observed at ZT21. Analysis of adverse effects revealed significantly higher hepatotoxicity in females compared with males, as well as a broader time window for occurrence of hepatotoxicity. In addition, female mice developed myotoxicity when dosed at ZT13, whereas no myotoxicity was observed in males at any time point. These results provide the first evidence of time-dependence and sex differences in both the therapeutic efficacy and adverse effects of simvastatin in OSCC. The limitations of this study include a need for further clinical trials to validate the findings, as well as deeper investigation of the reasons for the broader time windows for both optimal efficacy and adverse effects in female mice compared to males.

In summary, our study first revealed that PER2 binds to MYH9 and recruits TRIM21 to promote ubiquitin-dependent degradation of MYH9, thereby suppressing HMGCR expression to reduce intracellular cholesterol synthesis and inhibit OSCC cell proliferation. This study further revealed that the circadian clock gene PER2 regulates the circadian rhythms of HMGCR expression, intracellular cholesterol, and cancer cell proliferation in OSCC, with sex differences observed. The study further demonstrated that HMGCR inhibition with simvastatin at different circadian time points could yield over 2.5-fold differences in OSCC treatment efficacy, while also identifying sex differences in the optimal dosing time window and the timing of adverse effects. This study not only elucidates a novel mechanism by which the circadian clock gene PER2 reprograms cholesterol metabolism to inhibit OSCC, but also provides a theoretical basis and novel insights for precision cancer therapeutic strategies based on sex differences and time-dependent differences in circadian rhythms.

## Materials and methods

### Clinical samples

Between January 2022 and January 2023, 104 human OSCC tissues and paired adjacent normal tissues were collected from inpatients in the Department of Oral and Maxillofacial Surgery at the First Affiliated Hospital of Chongqing Medical University (Table S15). All specimens were collected during daytime hours between 10:00 and 15:00. All enrolled patients were pathologically confirmed as OSCC and had not received any treatment before surgery. All participants signed written informed consent, and the study was approved by the Ethics Committee of the First Affiliated Hospital of Chongqing Medical University (Approval No., 2021 - 588).

### Cell culture

HOK (ZQY012) and SCC15 (ZQ0758) cells were purchased from Shanghai Zhong Qiao Xin Zhou Biotechnology Co., Ltd. (China). The human OSCC cell lines CAL27 (iCell-h270) and SCC25 (iCell-h361) were obtained from Shanghai Cellverse Co., Ltd. (China). The mouse OSCC cell line MOC1 (CTCC-001-0916) was acquired from Zhejiang Meisen Cell Technology Co., Ltd. (China).

All cell lines were identified by short tandem repeat analysis and confirmed that there was no mycoplasma contamination. Cells were cultured in complete medium containing 89% DMEM (C11995500BT, Gibco, USA), 10% fetal bovine serum (ST30-3302, PAN, Germany), and 1% penicillin-streptomycin (C0222, Beyotime, China) with incubation at 37 °C and 5% CO₂.

### *PER2* overexpression and silencing

*PER2* overexpression and silencing lentiviruses were engineered by Shanghai GeneChem Co., Ltd. The primer sequences used to amplify the *PER2* gene for overexpression are provided in Table S16 and the short hairpin RNA sequences targeting different *PER2* sites (sh-PER2#1, sh-PER2#2, and sh-PER2#3) in Table S17. Hanheng Biotechnology Co., Ltd. (Shanghai, China) designed and produced three *PER2* deletion mutant plasmids: Flag-Mut-PER2^ΔPAS1^, Flag-Mut-PER2^ΔPAS2^, and Flag-Mut-PER2^ΔCT^. The primer sequences are provided in Table S18.

SCC15 and SCC25 cells were transduced with PER2-overexpressing lentivirus, while CAL27 cells were transduced with PER2-silencing lentivirus. Following puromycin selection, we established stable PER2-overexpressing cell lines (OE-PER2-SCC15 and OE-PER2-SCC25) and stable PER2-silenced CAL27 cell lines (sh-PER2-CAL27#1, sh-PER2-CAL27#2, and sh-PER2-CAL27#3). The negative controls were NC-SCC15 and NC-SCC25 for the overexpression groups and sh-NC-CAL27 for the silencing groups. The blank controls were denoted Blank-SCC15, Blank-SCC25, and Blank-CAL27. The three PER2 deletion mutant plasmids were transfected into SCC25 cells to obtain Mut-PER2^ΔPAS1^-SCC25, Mut-PER2^ΔPAS2^-SCC25, and Mut-PER2^ΔCT^-SCC25.

### Second-generation RNA-seq

Total RNA was collected from sh-PER2-CAL27#2 and sh-NC-CAL27 cells (*n* = 3). The quality of RNA was assessed using a NanoDrop 2000 and agarose gel electrophoresis. After library preparation with the Illumina TruSeq^TM^ kit, sequencing was performed on the Illumina platform. DESeq2 was employed to analyze differentially expressed genes between groups, with screening criteria of *P* < 0.05 and fold change >1.2, followed by GO and reactome enrichment analyses.

### Untargeted metabolomics analysis

Metabolites were extracted from NC-SCC25 and OE-PER2-SCC25 cells (*n* = 6). Raw mass spectrometry data were processed using Compound Discoverer 3.3 for metabolites identification and quantification. Differential metabolites were screened using the criteria of VIP > 1.0, |FC | > 1.2, and *P* < 0.05, followed by KEGG enrichment analysis.

### IHC detection

A IHC Detection Kit (PV-9000, ZSGB-BIO, China) was applied following the manufacturer’s protocol. In summary, 4-μm-thick paraffin sections were incubated with appropriate amounts of primary antibodies against PER2, HMGCR, and MYH9 at 4 °C overnight (See Table S19 for details). The next day, sections were sequentially incubated with secondary antibodies, developed with DAB, and counterstained with hematoxylin. Finally, samples were mounted and examined under a light microscope. The results were evaluated using the double grading and semiquantitative grading method.

### RT-qPCR assay

Following the manufacturer’s protocol, total RNA was isolated using RNAiso Plus (9109, TaKaRa, Japan) and subsequently reverse transcribed into cDNA with a PrimeScript FAST RT reagent Kit gDNA Eraser (RR092A, TaKaRa, Japan). Primer sequences for target genes *PER2*, *HMGCR*, *MYH9*, and *β-actin* are provided in Table S20. RT-qPCR was conducted following the protocol provided with TB Green® Premix Ex Taq (Tli RNaseH Plus) (RR820A, TaKaRa, Japan), with each sample analyzed in triplicate. The 2^−ΔΔCT^ method was employed to quantify the relative mRNA expression of the target genes, using *β-actin* as the reference gene.

### Western blotting

Cell lysis buffer was prepared by mixing RIPA buffer (P0013B, Beyotime, China), phosphatase inhibitor cocktail A (50×) (P1081, Beyotime, China), and PMSF (ST506, Beyotime, China) at a ratio of 100:2:1. Lysed samples were sonicated and centrifuged, and supernatants were collected. Protein quantification was performed with a BCA Assay Kit (P0010, Beyotime, China). Samples (20–30 μg) underwent 8–12% SDS-PAGE (P0012AC, Beyotime, China) separation and wet transfer to PVDF (IPVH00010, Millex, USA) membranes. After blocking with 5% skim milk for 1.5 h, membranes were incubated overnight at 4 °C with primary antibodies against PER2, HMGCR, MYH9, TRIM21, Flag, ubiquitin, and β-actin (details are provided in Table S19), followed by incubated for 1 h at room temperature with the corresponding HRP-conjugated secondary antibodies. An ultrasensitive chemiluminescent substrate (BMU102, Abbkine, China) was prepared, and signals were detected using an ECL-Advance Western Blotting Imaging System (Bio-Rad, USA), using β-actin as the loading control. Protein band intensities were quantified using ImageJ 5.0.

### Cholesterol assay

Working solutions of total cholesterol (TC) and free cholesterol (FC) were prepared under light-protected conditions according to the instructions provided with the Amplex® Red Cholesterol Assay Kit (A12216, Thermo Fisher, USA), and serial dilutions of cholesterol standards were prepared to generate a standard curve. Diluted samples and standards (50 μL/well) were loaded in triplicate into a 96-well black microplate. Following this, 50 μL of the corresponding cholesterol detection working solution was dispensed into each well. The plate was then mixed and incubated in the dark at 37 °C for 30 min. Finally, a microplate reader was used to measure the fluorescence intensity (excitation/emission: 560 nm/590 nm). After subtraction of blank control fluorescence values, cholesterol concentrations in samples were calculated based on the standard curve. CE content was determined by subtracting FC from TC values.

### Filipin staining

A cell suspension (2 × 10⁵ cells/mL) was seeded at 1 mL/well onto coverslips in six-well plates. Upon reaching 80% confluence, cells were fixed with 4% PFA for 30 min at room temperature, then treated with 1 mL of 1.5 mg/mL glycine solution per well to neutralize residual PFA. Cells were then stained with 1 mL of 0.1 mg/mL Filipin working solution (HY-N6716, MCE, USA) and incubated in darkness at room temperature for 2 h. The samples were mounted with antifade reagent, labeled, and imaged with an inverted fluorescence microscope at 340–380 nm excitation.

### CCK8 assay

Cells were seeded into a 96-well plate at a density of 1 × 10⁴ cells/mL, with 100 μL per well, and three replicates were set for each group. At 24, 48, 72, and 96 h, the medium was replaced with 100 μL fresh culture medium containing 10% CCK-8 reagent (AC11L054, Life-iLab, China). The plates were then incubated at 37 °C in the dark for 2 h before measuring absorbance at 450 nm using a microplate reader. Cell growth curves were plotted with time on the *x*-axis and the mean absorbance of each group on the *y*-axis.

### MTT assay

An MTT assay was conducted using the BL132B kit (Biosharp, China). Cells were seeded into a 96-well plate at a density of 1 × 10⁴ cells/mL, with 100 μL per well, and three replicates were set for each group. At 24, 48, 72, and 96 h, 50 μL of 1 × MTT solution was dispensed into each well, followed by 4 h incubation at 37 °C in the dark. The supernatant was then removed, and formazan crystals were dissolved in 150 μL DMSO. Absorbance was measured at 490 nm, and cell growth curves were plotted as above.

### Cell synchronization and circadian rhythm detection

Cells in good growth condition were seeded in culture dishes containing complete medium. When cell density reached approximately 60%, the complete medium was replaced with serum-free medium containing dexamethasone at a final concentration of 1 μmol/L. After incubation in a cell culture incubator for 2 h, the medium was replaced again with complete medium. This time point was designated as Zeitgeber time 0 (ZT0). Cells continued to be cultured, and six time points (ZT1, ZT5, ZT9, ZT13, ZT17, and ZT21) were set at 4-hour intervals over a 24-h period. At each time point, cells were collected to analyze HMGCR expression, intracellular cholesterol, and cell proliferation for circadian rhythm analysis.

### Co-IP

Co-IP samples were prepared according to the kit’s instructions (Protein A + G Magnetic Beads) (P2179, Beyotime, China) by lysing cells with a buffer containing NP-40, phosphatase inhibitor cocktail A (50×), and protease inhibitor cocktail (100×) at a ratio of 100:2:1. A small lysate aliquot was saved as input control. The remaining lysate was incubated with antibodies against PER2, TRIM21, or MYH9 overnight at 4 °C. Protein A/G magnetic beads were added, and the samples were rotated at 4 °C for 4 h. After magnetic separation and TBS washing, the immunoprecipitates were eluted in 1 × SDS loading buffer and denatured at 95 °C for 5 min.

### IP-MS analysis

SCC25 cells were transfected with Flag-tagged pcDNA-PER2 plasmids, with pcDNA3.1 empty vector as a negative control. Cells were collected 48 h post-transfection, and protein samples were prepared following Co-IP protocols to obtain PER2-interacting proteins. The resulting samples were resolved by SDS-PAGE, and target protein bands were identified via Coomassie Brilliant Blue staining. Bands of interest were excised and hydrated with 800 μL ultrapure water. Finally, protein identification was performed using a TripleTOF 6600 mass spectrometer (AB SCIEX, USA).

### GST pull-down

Using an *Escherichia coli* expression system, we separately expressed both GST control and GST-PER2 fusion proteins. Experiments were conducted with a Pierce GST Protein Interaction Pull-Down Kit (21516, Thermo Fisher, USA) according to the manufacturer’s protocols. Bacterial lysates and glutathione agarose beads were incubated at 4 °C for 1 h to isolate GST-PER2 fusion proteins. Subsequently, the GST-PER2 fusion proteins and the target binding proteins were incubated in vitro. After eluting the bound complex with glutathione elution buffer, western blotting experiments were performed.

### Protein–protein docking prediction

Protein models were downloaded from the AlphaFold Protein Structure Database (https://alphafold.ebi.ac.uk/) for PER2 (UniProt ID: O15055), MYH9 (UniProt ID: P35579), and TRIM21 (UniProt ID: P19474) and preprocessed in PyMOL 2.4. Protein–protein docking was performed using the HDOCK server (http://hdock.phys.hust.edu.cn/) to identify the top ten best docking poses. The highest-scoring conformation was selected as the optimal docking model. All results were visualized using PyMOL 2.4.

### Immunofluorescence staining and confocal microscopy analysis

Inoculate 2 × 10⁵ cells onto coverslips in six-well plates. Upon reaching 70% confluence, the cells were fixed with 4% PFA, then permeabilization with 0.2% Triton X-100, and blocked with ready-to-use goat serum. Next, the cells were incubated with primary antibodies against MYH9 or TRIM21 at 4 °C overnight. The following day, cells were incubated with the corresponding fluorescent secondary antibodies (Alexa Fluor 555/647) for 1 h protected from light. After PBS washing, cells were blocked again and incubated with CoraLite®488-conjugated PER2 primary antibody overnight at 4 °C in the dark (details in Table S21). Images were acquired using confocal microscopy (Andor Microscopes, UK).

### Ubiquitination assay

When cells reached approximately 70% confluence, they were incubated with 20 μM MG132 (HY-13259, MCE, USA) for 4 h at 37 °C before harvesting. Protein samples were extracted following standard Co-IP procedures. Proteins pulled down using MYH9 as bait were analyzed by western blotting to assess MYH9 expression levels. After chemiluminescent detection, the PVDF membrane was treated with western antibody stripping buffer (P0025B, Beyotime, China), blocked with 5% skim milk, and then incubated with ubiquitin primary antibody at 4 °C overnight, followed by HRP-conjugated secondary antibody at room temperature for 1 h before ECL imaging.

### CHX chase assay

NC-SCC25 and OE-PER2-SCC25 cells (2 × 10⁵ cells/well) were plated in six-well plates. Upon reaching roughly 70% confluency, the culture medium was replaced with fresh medium containing 40 μg/mL CHX (C112766, Aladdin, China). Cells were harvested at 0 h, 3 h, 6 h, 9 h, 12 h and 15 h post-CHX treatment, and total protein was extracted for western blotting analysis.

### Enzyme-linked immunosorbent assay

The mouse HMGCR standard (MU30714, Bioswamp, China) was serially diluted two-fold to generate concentrations of 1200, 600, 300, 150, 75, and 0 pg/mL. Each concentration was assayed in duplicate with 50 μL per well. For sample detection, 40 μL of test sample and 10 μL of biotinylated antibody were added per well, followed by 50 μL of enzyme conjugate reagent. After 30 min incubation at 37 °C, plates were washed five times, and 50 μL each of chromogen substrates A and B were added for 10-min color development at 37 °C in the dark. The reaction was terminated with 50 μL stop solution, and OD450 values were measured within 15 min after termination. A standard curve was generated by plotting standard concentrations (*x*-axis) against OD values (*y*-axis) with four-parameter logistic regression and employed to determine sample concentrations.

### In vivo tumorgenicity, sex differences in circadian rhythms, and chronotherapy

A total of 34 male BALB/c-nu nude mice (8–20 g, aged 5–6 weeks), 71 male C57BL/6 J mice (26–29 g, aged 12 weeks), and 71 female C57BL/6 J mice (20–22 g, aged 12 weeks) were purchased from Chengdu Yaokang Biotechnology Co., Ltd. All animals were housed under specific-pathogen-free conditions at the Laboratory Animal Center of Chongqing Medical University. Experimental protocols involving animals were approved by the Institutional Animal Care and Use Committee of Chongqing Medical University (approval number: IACUC-CQMU-2024-0028). The investigators were not blinded to group allocation during animal experiments and outcome assessment.

To investigate the inhibitory effect of *PER2* on cholesterol synthesis in OSCC, ten male nude mice were randomly allocated to either the NC-SCC25 group or OE-PER2-SCC25 group (*n* = 5 per group). Each mouse received a 200 μL subcutaneous injection of either NC-SCC25 or OE-PER2-SCC25 cell suspension (5 × 10⁶ cells/mL) in the left dorsal flank. After 28 days, the mice were euthanized by cervical dislocation, and tumors were excised for further analysis.

To evaluate the therapeutic efficacy of PER2 overexpression combined with simvastatin in the treatment of OSCC, 24 male nude mice were randomly assigned to four groups (*n* = 6 per group). Each mouse received a 200 μL subcutaneous injection of NC-SCC25 or OE-PER2-SCC25 cell suspension (5 × 10⁶ cells/mL) in the left dorsal flank. Treatment was initiated when tumor volumes reached approximately 100 mm^3^. The NC-SCC25 + NS group acted as controls. The NC-SCC25+simvastatin and OE-PER2-SCC25+simvastatin group received daily intraperitoneal injections of simvastatin (10 mg/kg) (HY-17502, MCE, USA) for 4 weeks; All treatments were administered within 10:00 ± 10 min each day. On day 42, mice were euthanized by cervical dislocation, and the tumors were excised.

To evaluate circadian characteristics and sex differences in *PER2* and *HMGCR* expression, intracellular cholesterol, and cell proliferation, 72 C57BL/6 J mice (36 males and 36 females) were randomly divided into six male groups and six female groups (*n* = 6 per group). All mice were acclimatized for 2 weeks under a 12-h light/12-h dark cycle for circadian synchronization. Zeitgeber time was defined with light onset at 08:00 (ZT0) and light offset at 20:00 (ZT12), where ZT0–ZT12 represented daytime and ZT12–ZT24 nighttime. Each mouse received a 200 μL subcutaneous injection of mouse OSCC MOC1 cell suspension (5 × 10⁷ cells/mL) in the left dorsal flank. On day 28, male and female C57BL/6 J mice (6 per group) were euthanized by cervical dislocation at six different time points within 24 h (ZT1, ZT5, ZT9, ZT13, ZT17, and ZT21). The tumors were then harvested for subsequent analysis.

To evaluate the time-dependent effects and sex differences of simvastatin treatment in OSCC, 35 male and 35 female C57BL/6 J mice were randomly assigned to seven male groups and seven female groups (*n* = 5 per group). The light/dark cycle, housing conditions, and ZT time definitions were as described above. After 2 weeks of acclimatization for circadian synchronization, A 200 μL subcutaneous injection of MOC1 cell suspension (5 × 10⁷ cells/mL) was administered to the left dorsal flank of each mouse. Treatment began when tumor volumes reached approximately 100 mm³. The MOC1 + NS group served as the control. Each of the other six groups received intraperitoneal injection of simvastatin (10 mg/kg) at one of six different time points (ZT1, ZT5, ZT9, ZT13, ZT17, and ZT21) once daily for 4 weeks. On day 42, at the same time point, terminal blood collection was performed via orbital puncture under anesthesia, followed by euthanasia through cervical dislocation. Tumors were harvested, and serum was separated for analysis.

All tumors were weighed using an electronic balance. For volume assessment, the maximum length (*a*) and minimum width (*b*) were measured with vernier calipers, and volumes were calculated as *V* = 0.5 × *a* × *b*².

### Bioinformatics analysis

Gene expression and clinical data for OSCC were downloaded from TCGA (https://portal.gdc.cancer.gov). Patients were stratified into high- and low-risk groups according to *MYH9* and *HMGCR* gene expression levels, with optimal cutoff values determined using the maxstat.test, followed by Kaplan–Meier survival analysis. The GSE30784 dataset was obtained from the GEO (https://www.ncbi.nlm.nih.gov/geo/). The Wilcoxon test was used to analyze differentially expressed genes, and correlations between *PER2* and *MYH9* expression were assessed using Spearman’s method.

### Statistical analysis

Sample size was determined based on previous studies and preliminary experiments. No statistical method was used to estimate sample size. All statistical analyses were executed using GraphPad Prism 8.0 (California, USA) software, experimental data from three biological replicates were expressed as mean ± SD. All quantitative data first underwent normality testing (Shapiro-Wilk test) and homogeneity of variance testing (Brown-Forsythe test). For data meeting the assumptions of parametric tests, differences between the two groups were assessed using unpaired Student’s *t*-tests, one-way analysis of variance (One-way ANOVA) was used for one-way multi-group comparisons, and two-way analysis of variance (Two-way ANOVA) was used for two-way multi-group comparisons. For any ANOVA results showing significant differences, post-hoc pairwise comparisons were corrected using Tukey’s HSD test. Cosine fitting analysis was conducted using Origin 2024 with fitting equation *Y* = *M* + *A*·cos [2π/*p* × (*t* + Φ)], where *M* represents the mesor, *A* the amplitude, *p* the period, Φ the acrophase, and *t* the time point. A gene was considered to exhibit circadian rhythmicity if it simultaneously satisfied both ANOVA (*P* < 0.05) and cosine analyses (*P* < 0.05). Differences in mesor and amplitude between cosine curves were compared using CircaCompare [53]. All experiments were independently performed at least three times, *P* < 0.05 indicated statistical significance.

## Supplementary information


Supplementary Figures 1-6
Table S1
Table S2
Table S3
Table S4
Table S5
Table S6
Table S7
Table S8
Table S9
Supplementary Tables10-14
Table S15
Supplementary Tables16-21
Supplementary Information


## Data Availability

This study includes RNA-seq data that have been deposited in the NCBI Sequence Read Archive (SRA) under accession number PRJNA1308766. The proteomics datasets have been deposited to the iProX repository under accession number IPX0009277000. The untargeted metabolomics data generated in this study are available from the corresponding author upon reasonable request. All other data supporting the findings of this study are included in this article and its supplementary information files.
