# Supplementary material for: PER2 reprograms intracellular cholesterol synthesis to inhibit oral squamous cell carcinoma and the chronotherapeutic efficacy of simvastatin
Source: Cell Death Discov. 2026 Jun 20;12:351. doi: 10.1038/s41420-026-03209-5 (PMC13529778; doi:10.1038/s41420-026-03209-5)

Supplementary Material

Original western blot images for

PER2 reprograms intracellular cholesterol synthesis to inhibit oral squamous cell carcinoma and the chronotherapeutic efficacy of simvastatin

Shilin Yin[1], Fang Yang[1,2], Zhiwei Zhang[1], Mingyuan Liu[1], Hong Tang[1], Kai Yang[1*]

Fig.1 E

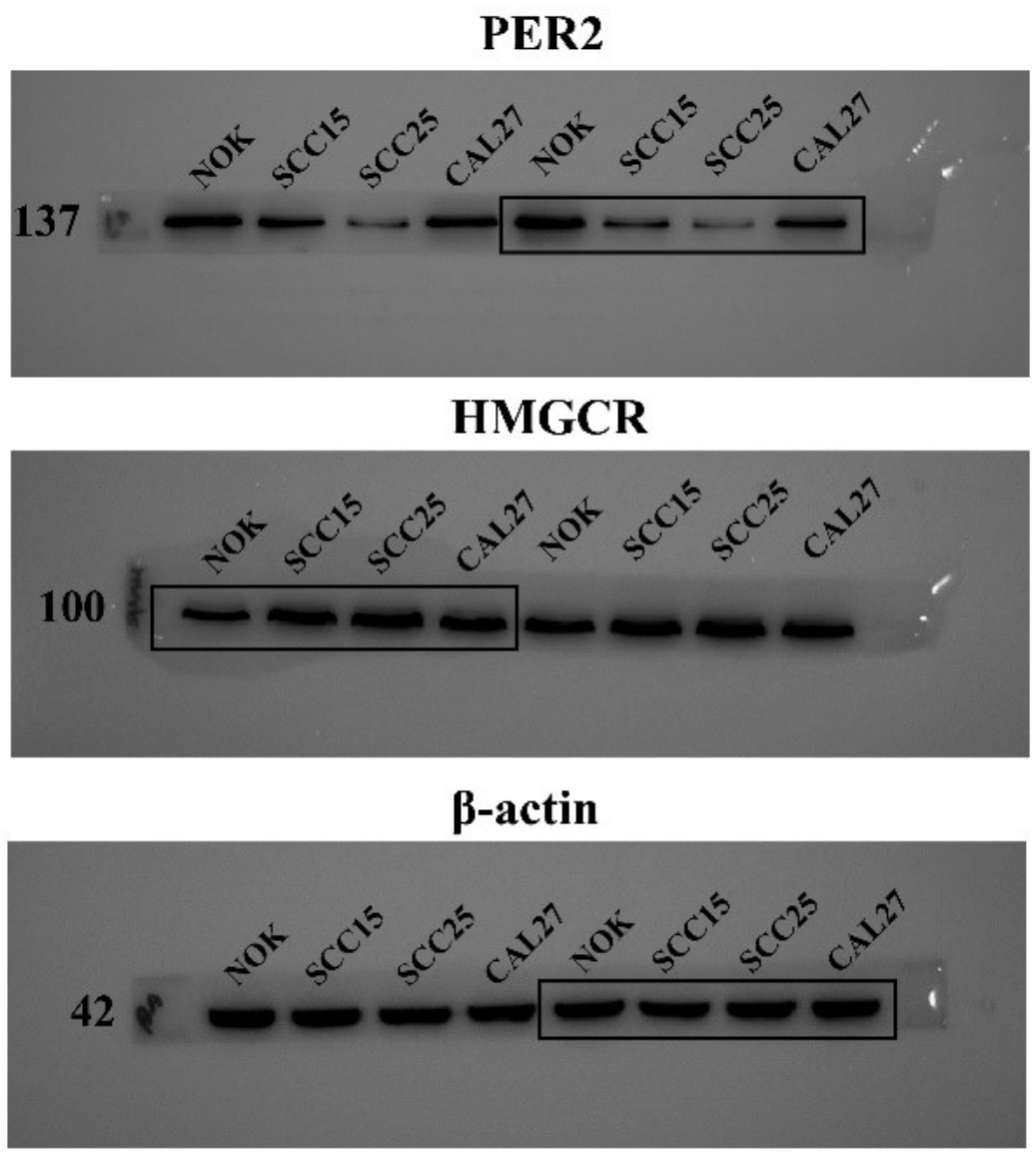

Fig.2 G

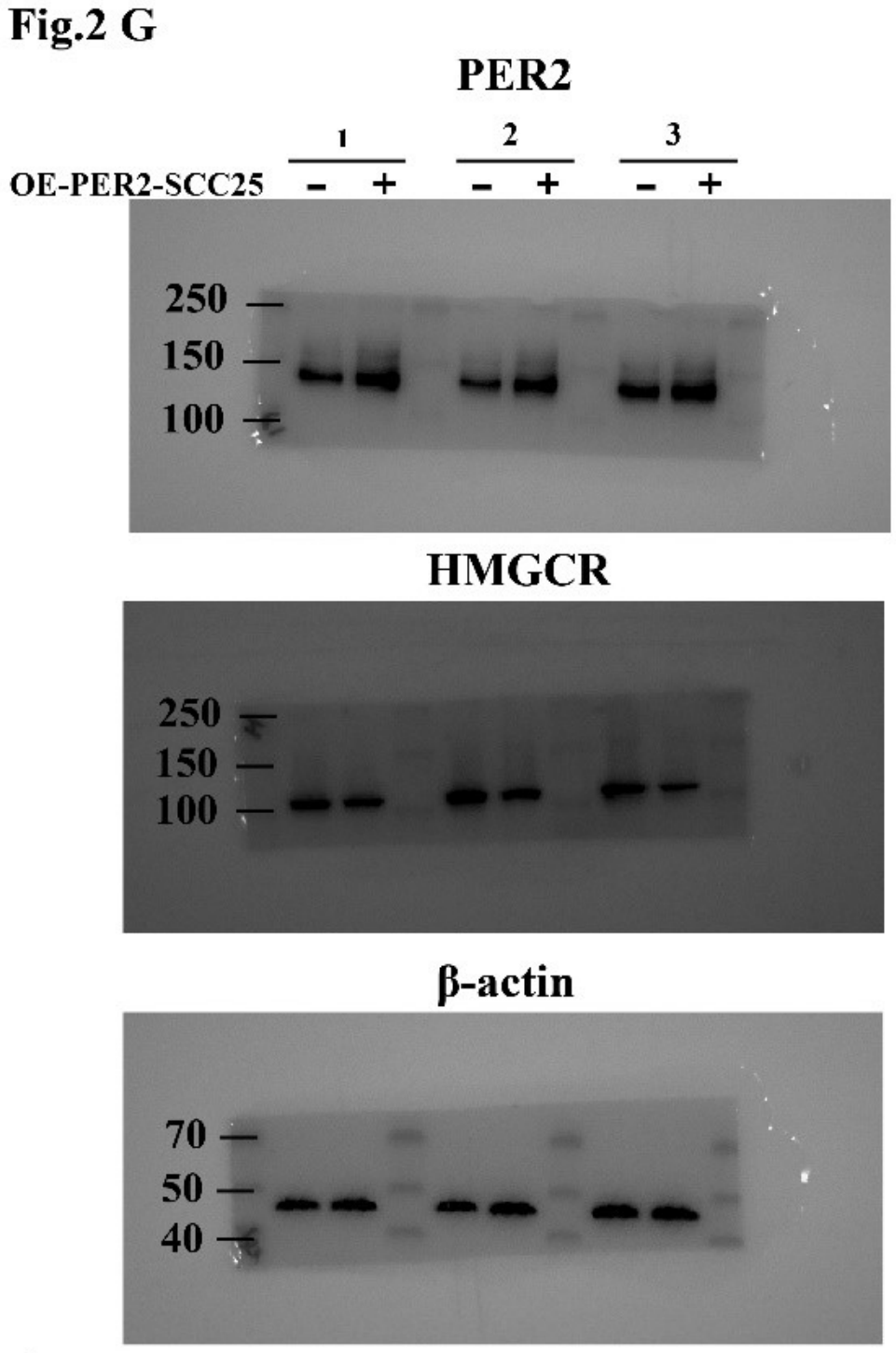

Fig.2 B

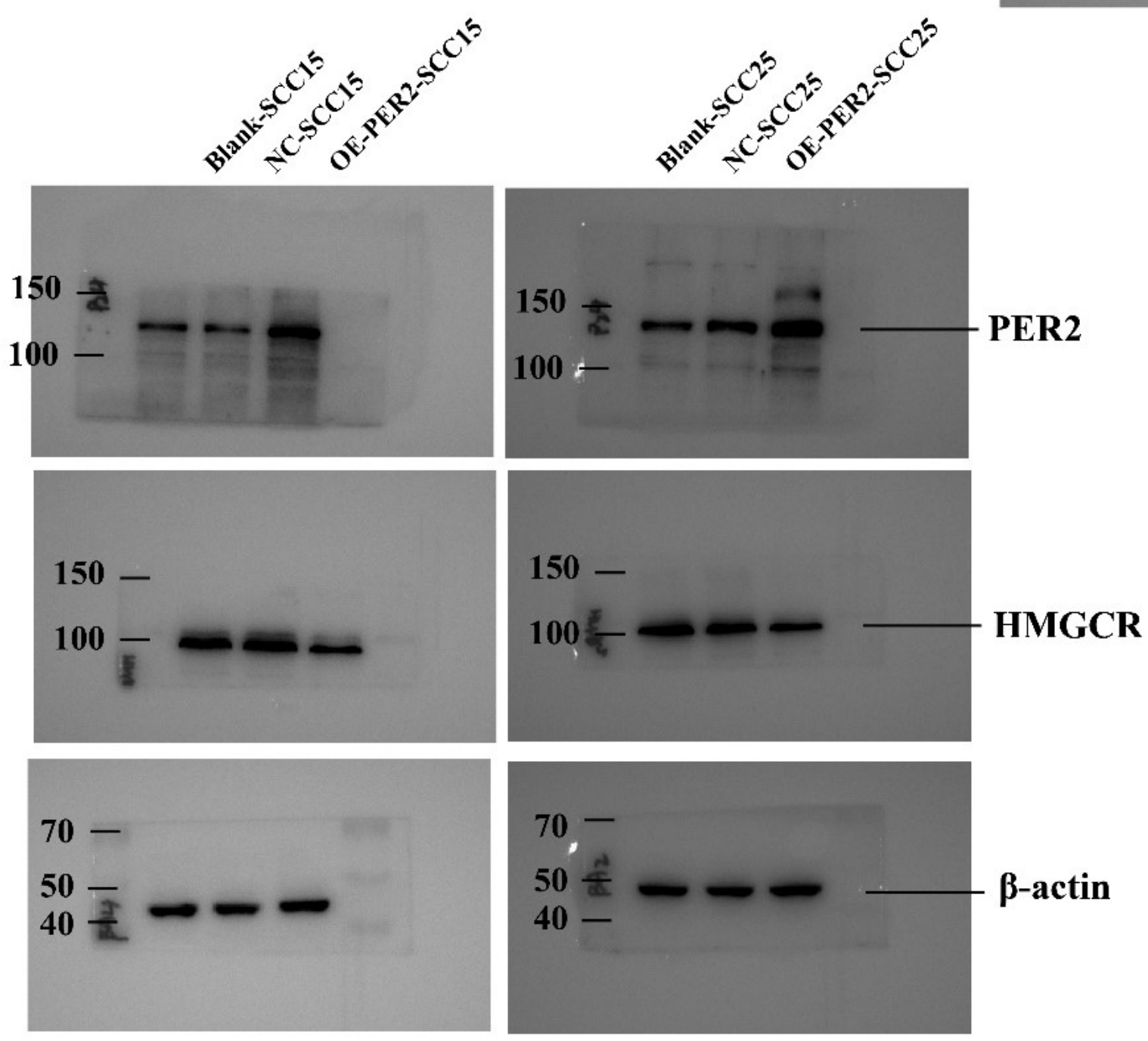

Fig.3 F

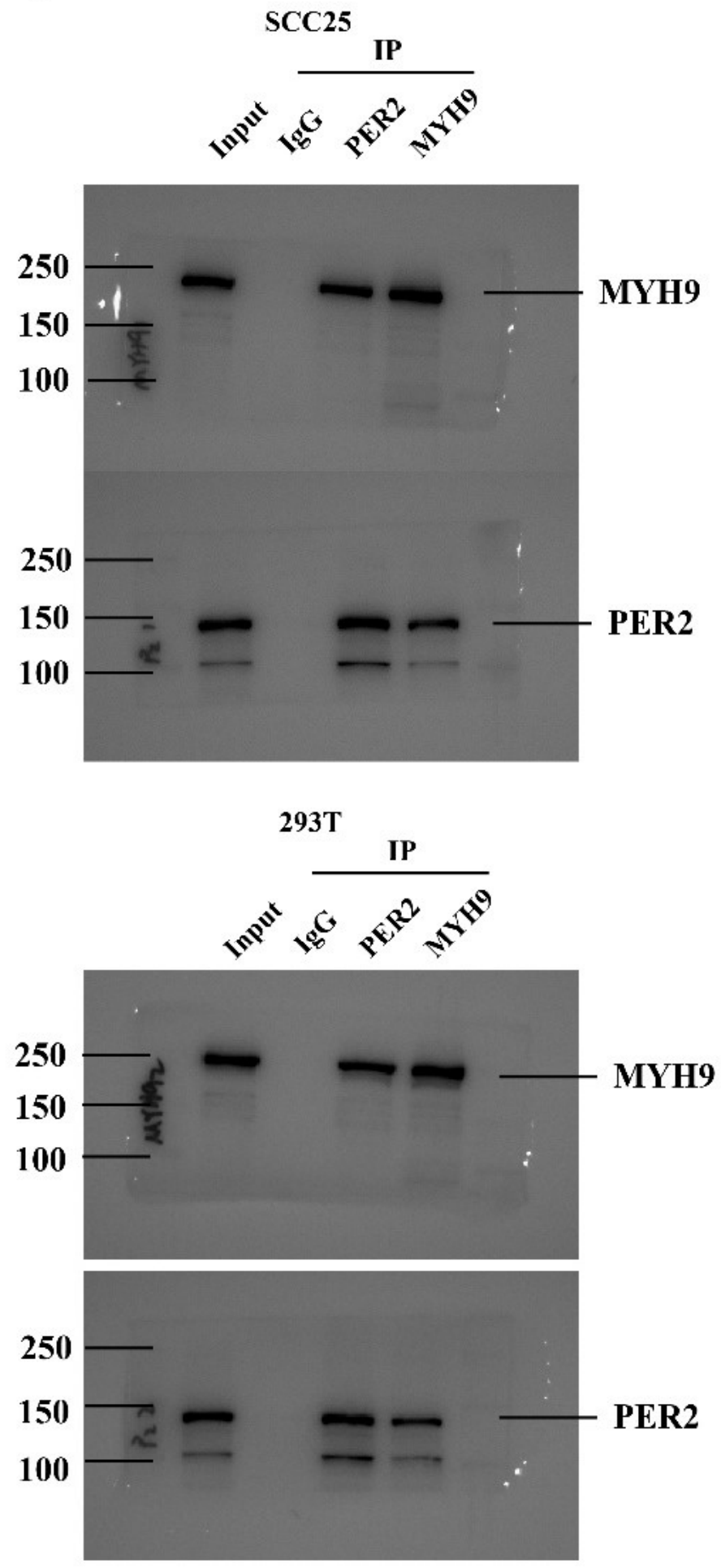

Fig.3 H

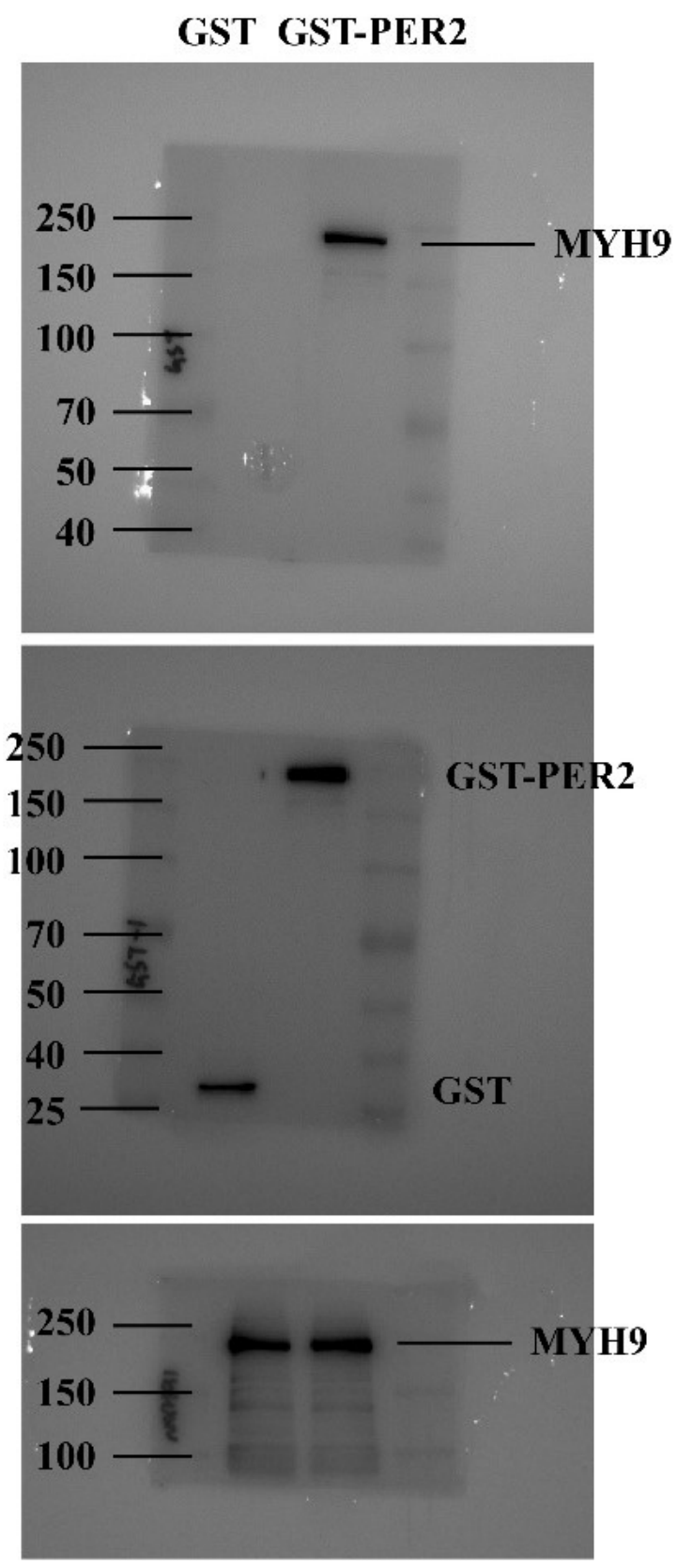

Fig.3L

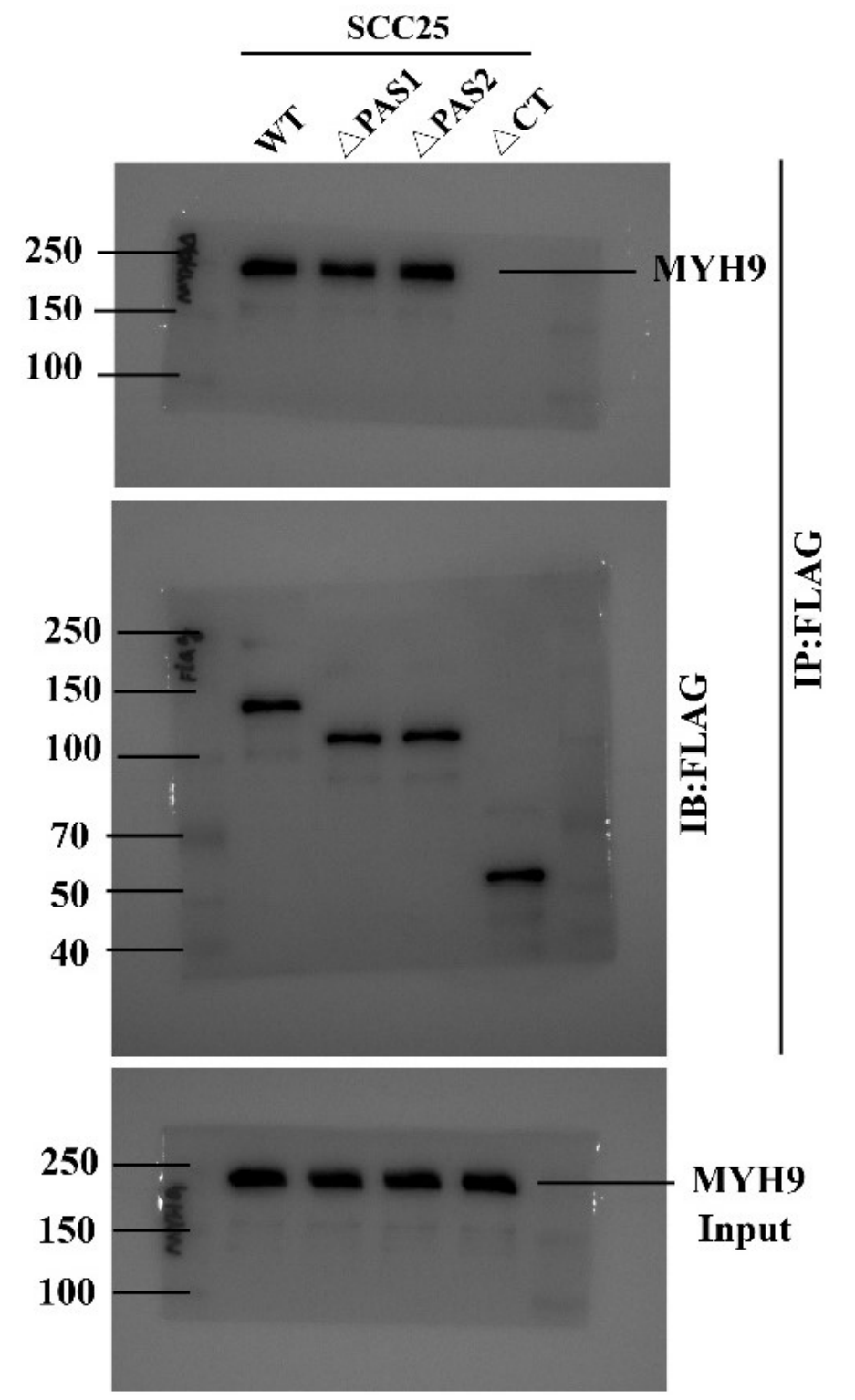

Fig.3M

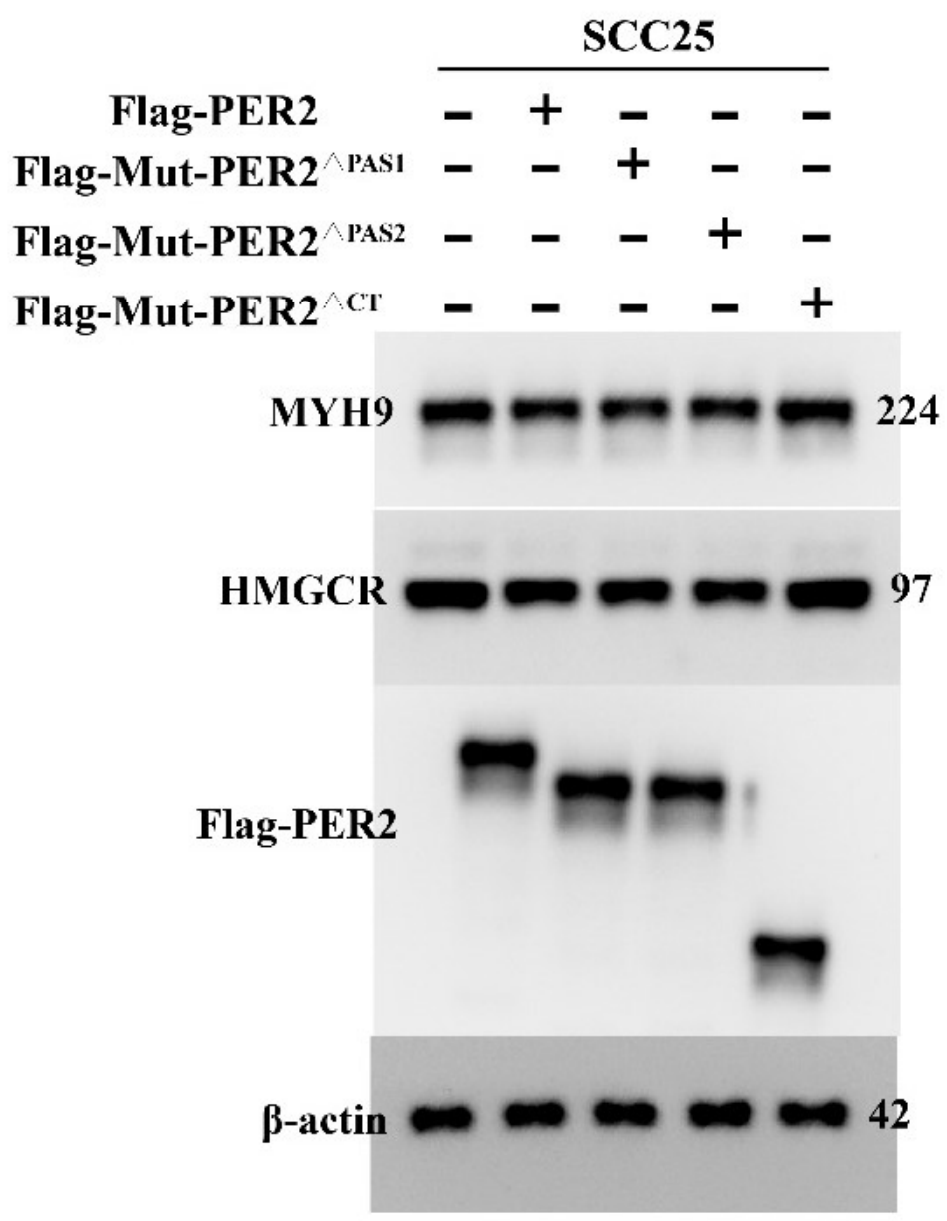

Fig.4 B

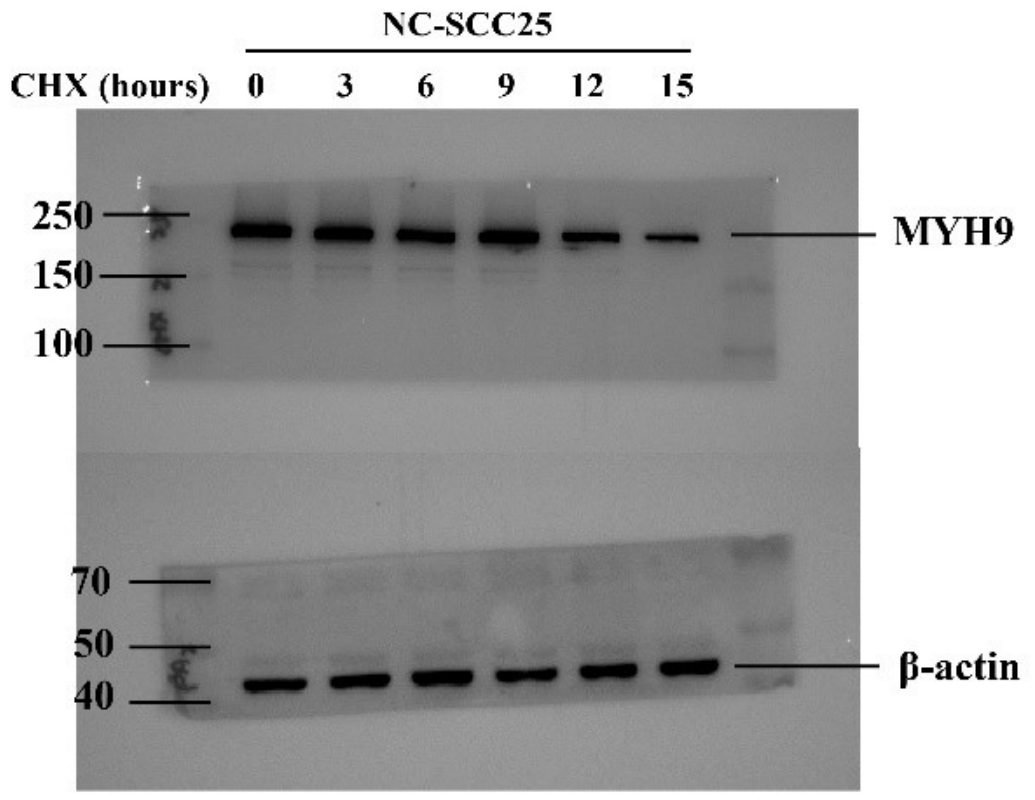

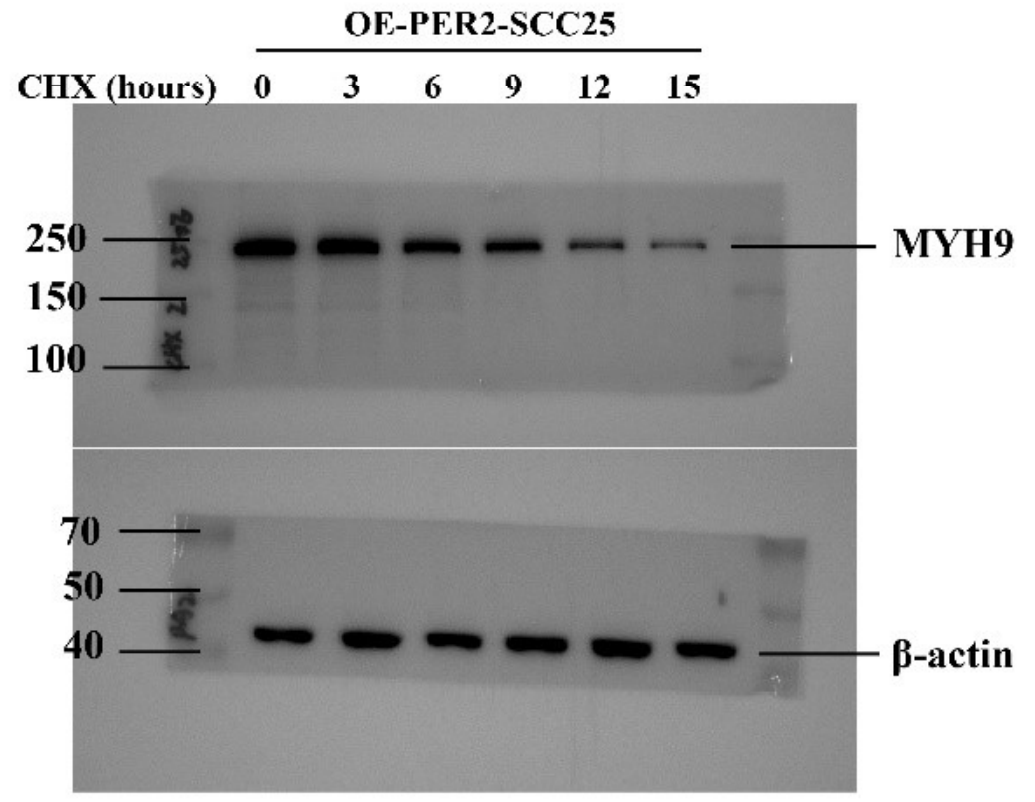

Fig.4 C

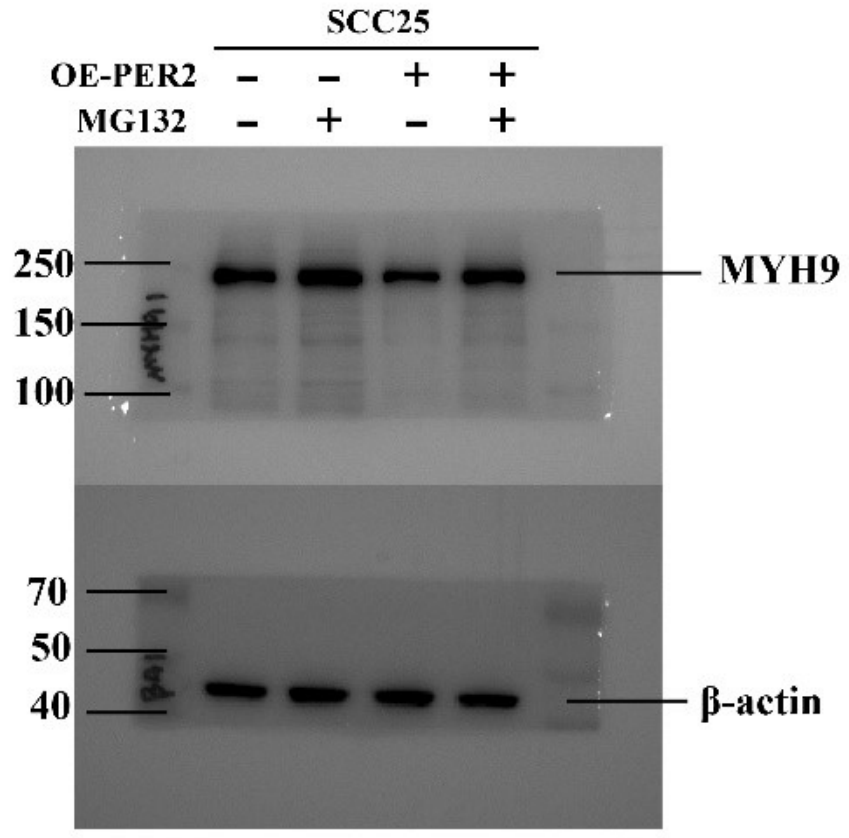

Fig.4 D

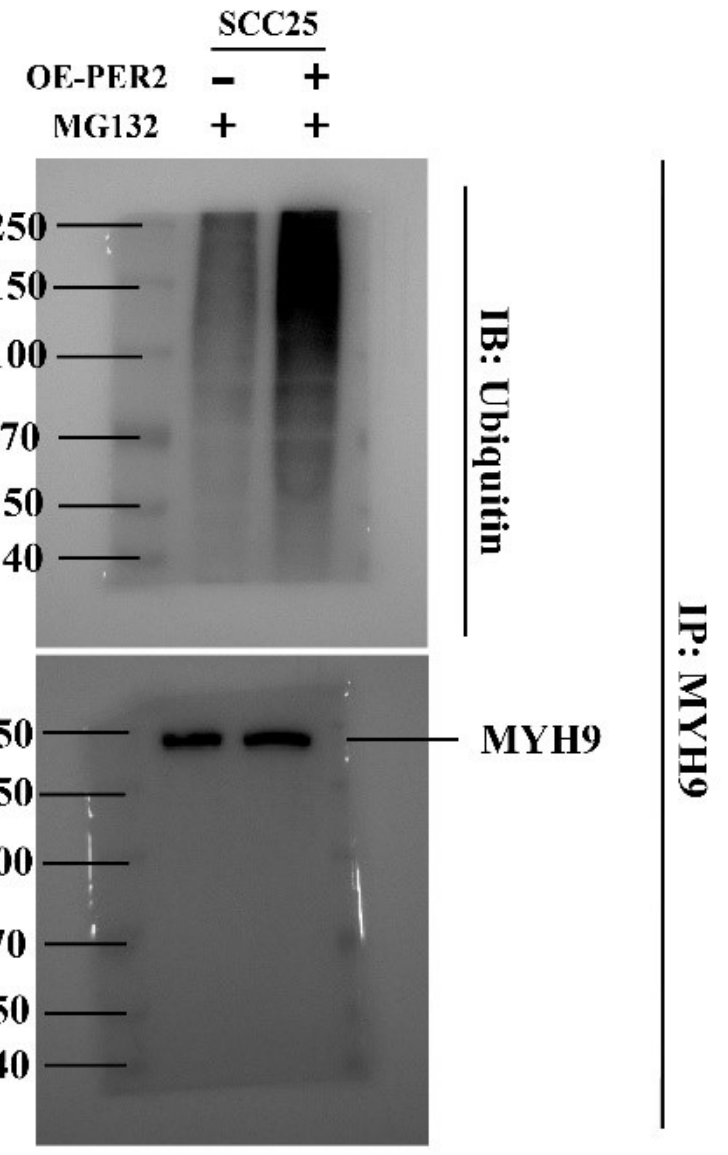

Fig.4 F

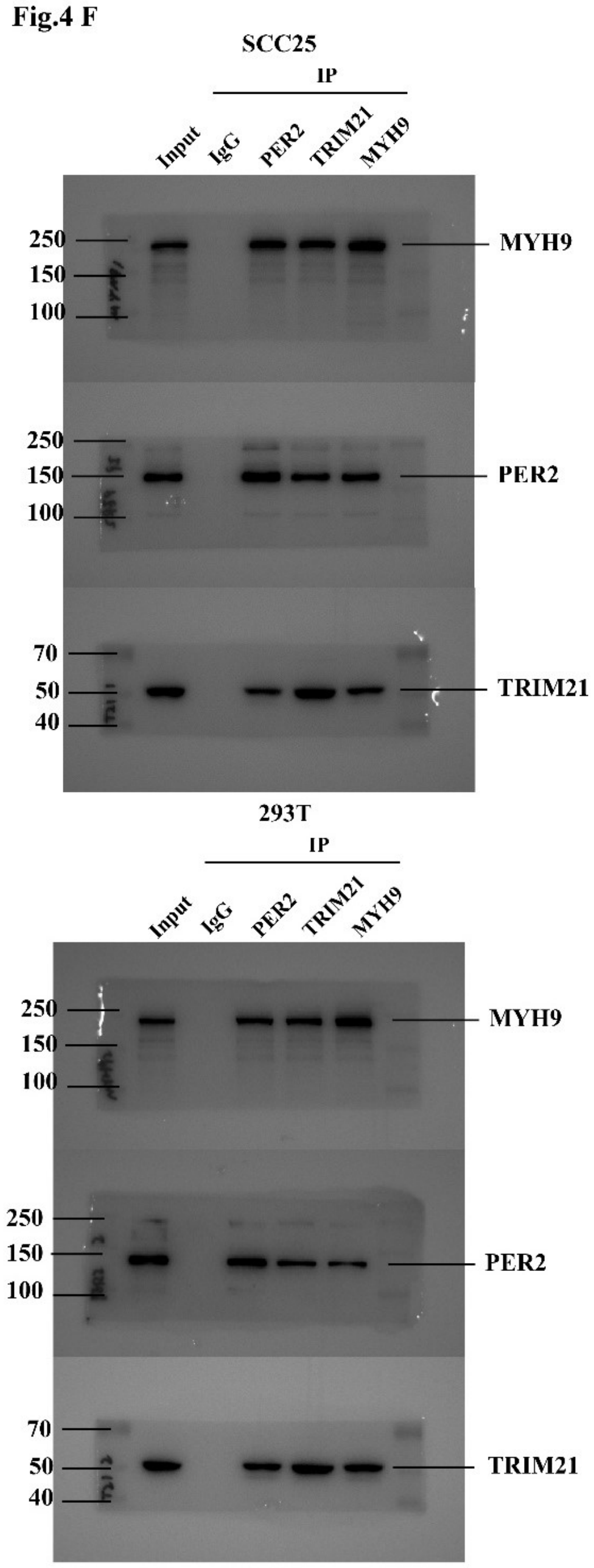
SCC25
IP
Input
IgG
PER2
TRIM21
MYH9
250
150
100
MYH9
250
150
100
PER2
70
50
40
TRIM21
293T
IP
Input
IgG
PER2
TRIM21
MYH9
250
150
100
MYH9
250
150
100
PER2
70
50
40
TRIM21

Fig.4 G

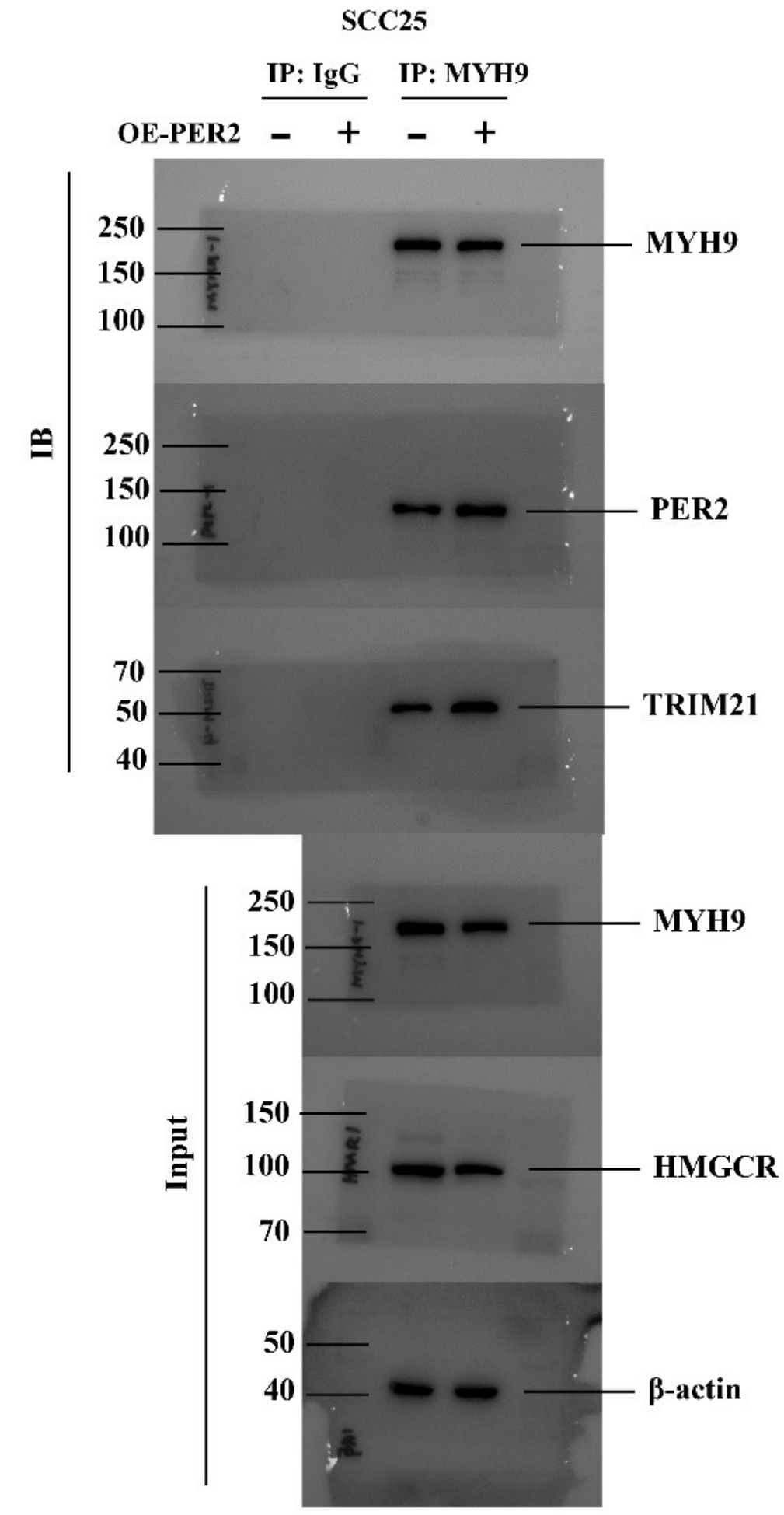
SCC25
IP: IgG
IP: MYH9
OE-PER2
–
+
–
+
IB
250
150
100
MYH9
250
150
100
PER2
70
50
40
TRIM21
Input
250
150
100
MYH9
150
100
70
HMGCR
50
40
β-actin

Fig.6A

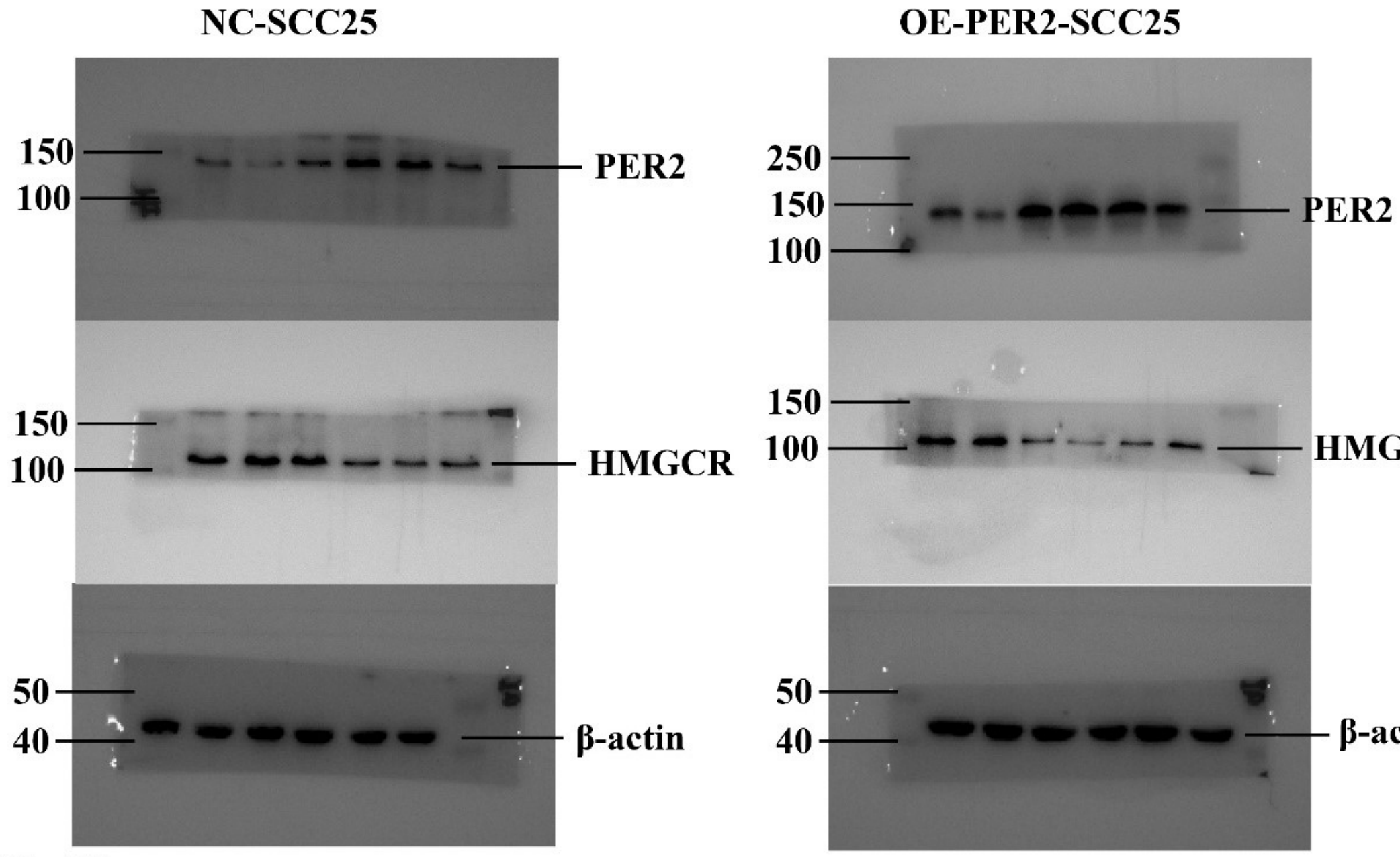
NC-SCC25
150
100
PER2
150
100
HMGCR
50
40
β-actin
OE-PER2-SCC25
250
150
100
PER2
150
100
HMGCR
50
40
β-actin

Fig.6D

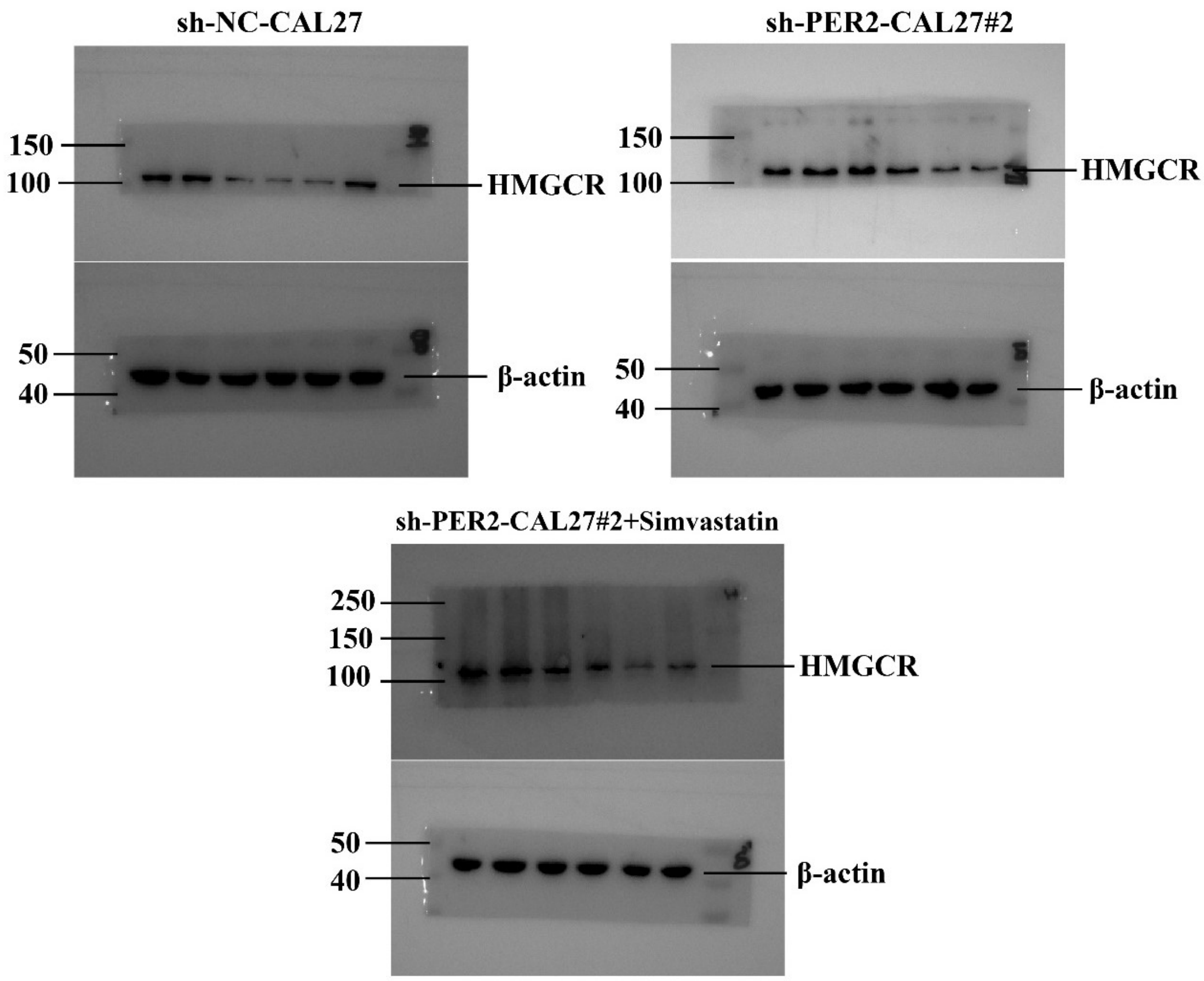
sh-NC-CAL27
150
100
HMGCR
50
40
β-actin
sh-PER2-CAL27#2
150
100
HMGCR
50
40
β-actin
sh-PER2-CAL27#2+Simvastatin
250
150
100
HMGCR
50
40
β-actin

Fig.7C

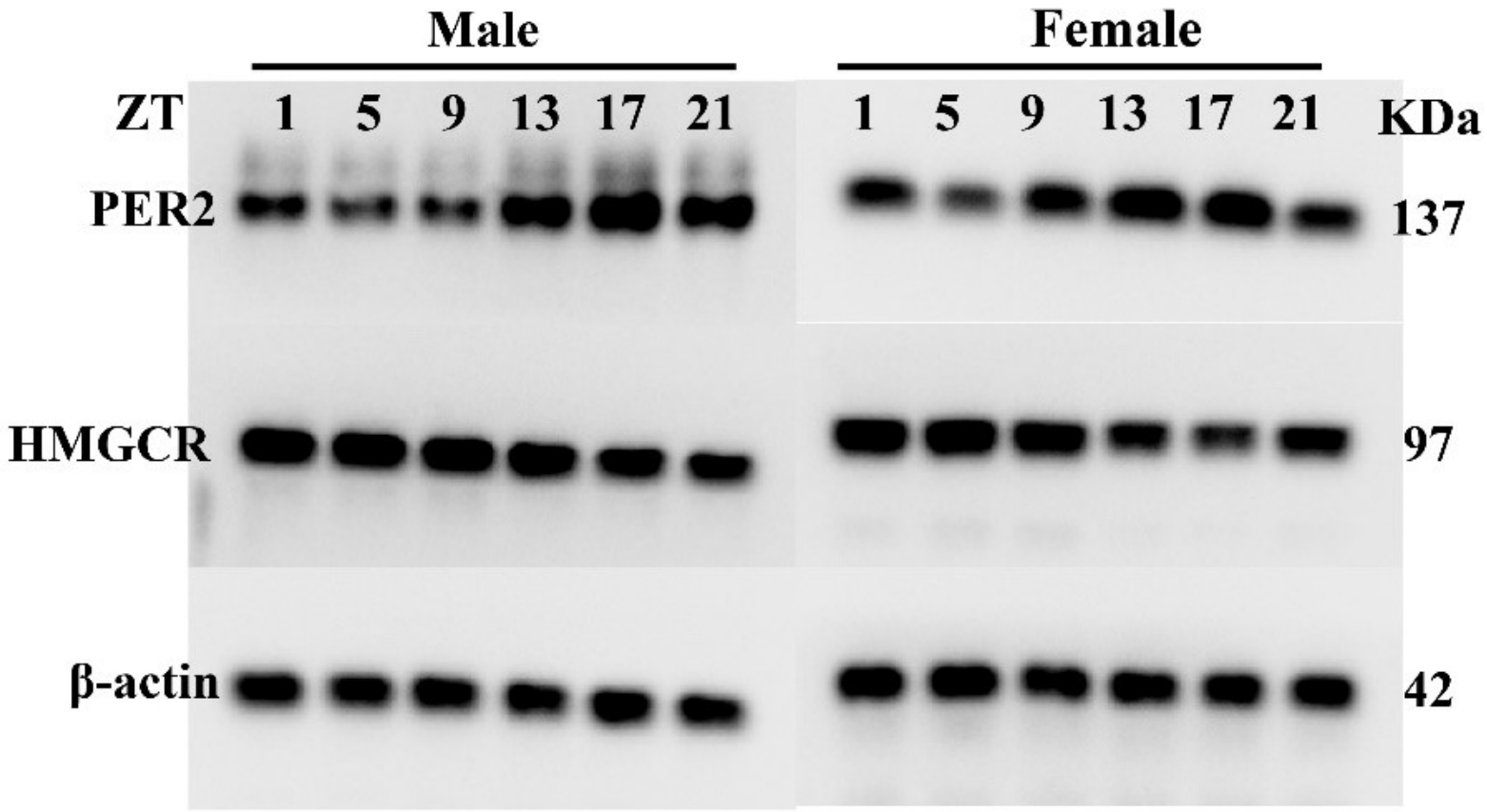

Supplementary Fig.1B

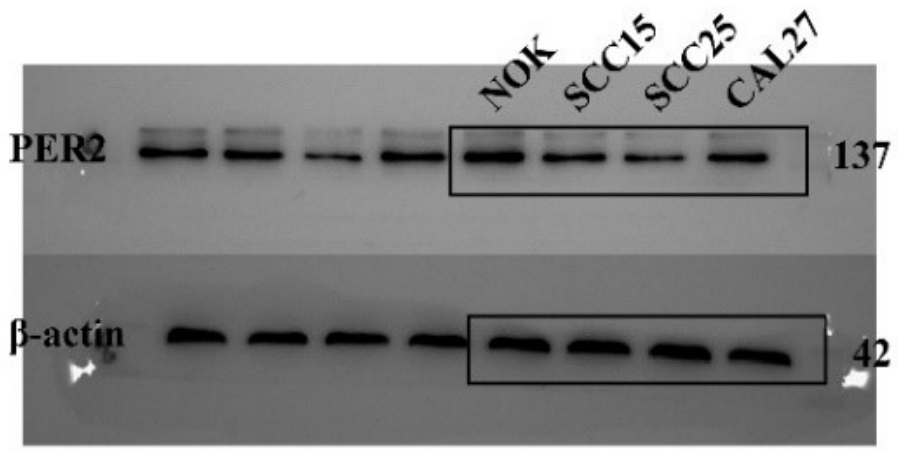

Supplementary Fig.2B

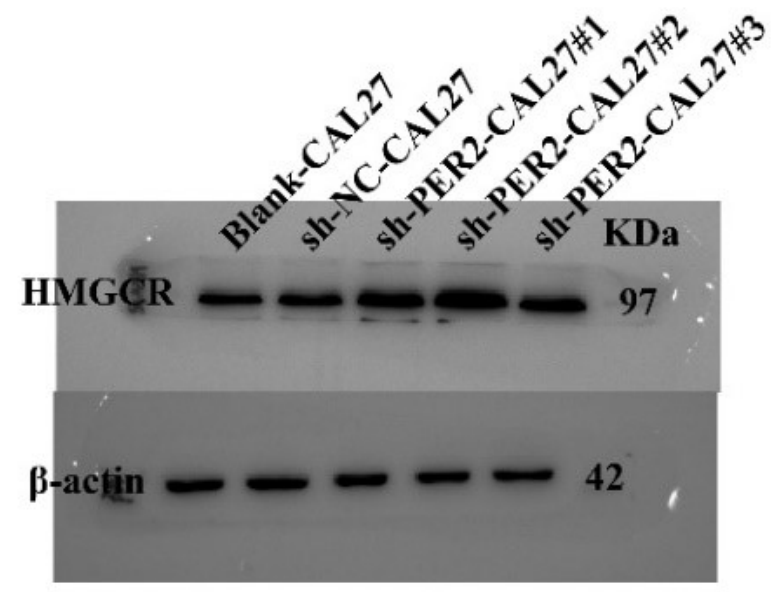

Supplementary Fig.1D

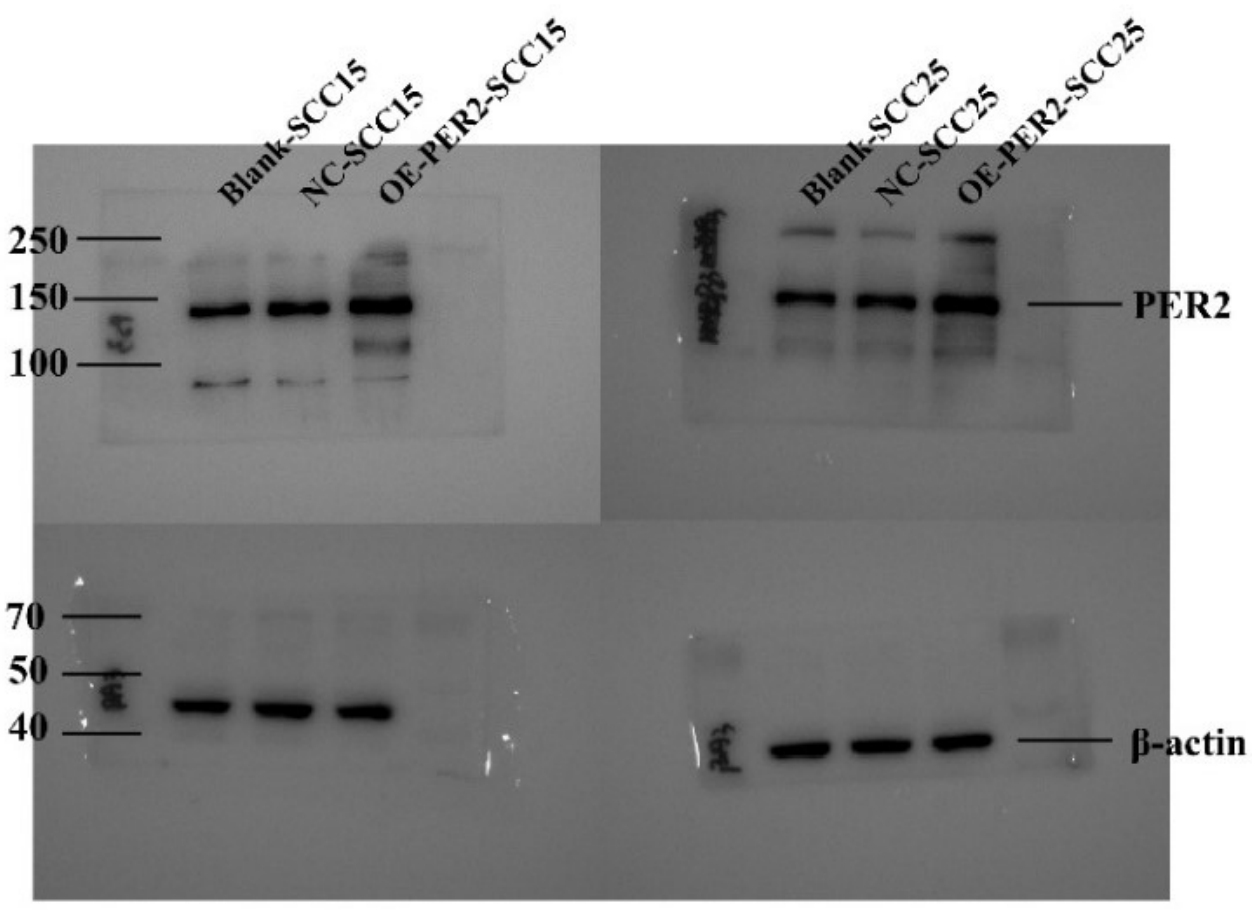

Supplementary Fig.2F

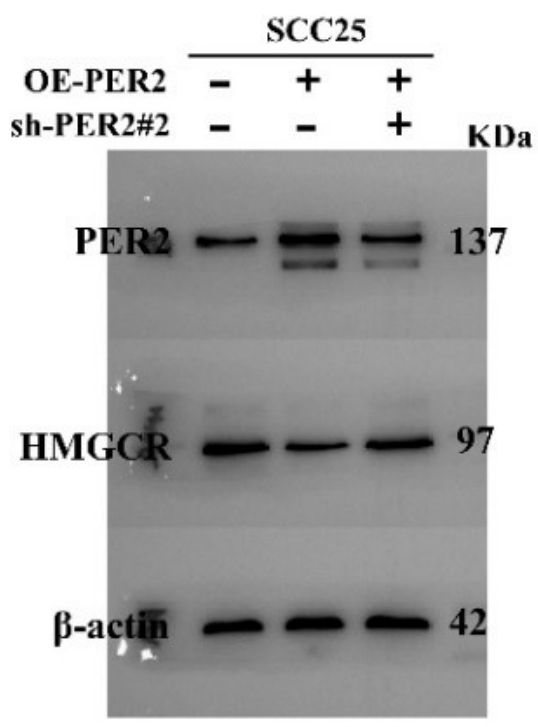

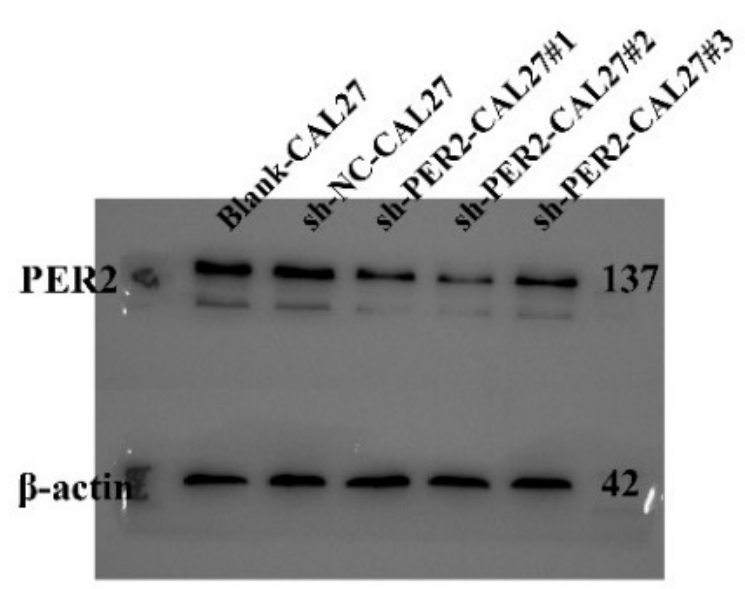

Supplementary Fig.2J

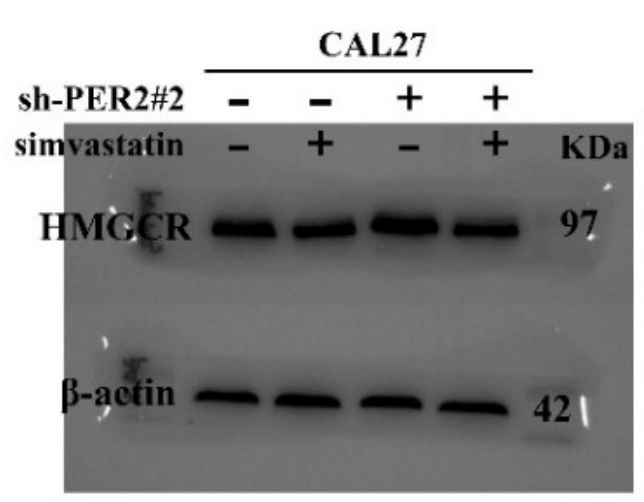

Supplementary Fig.3A

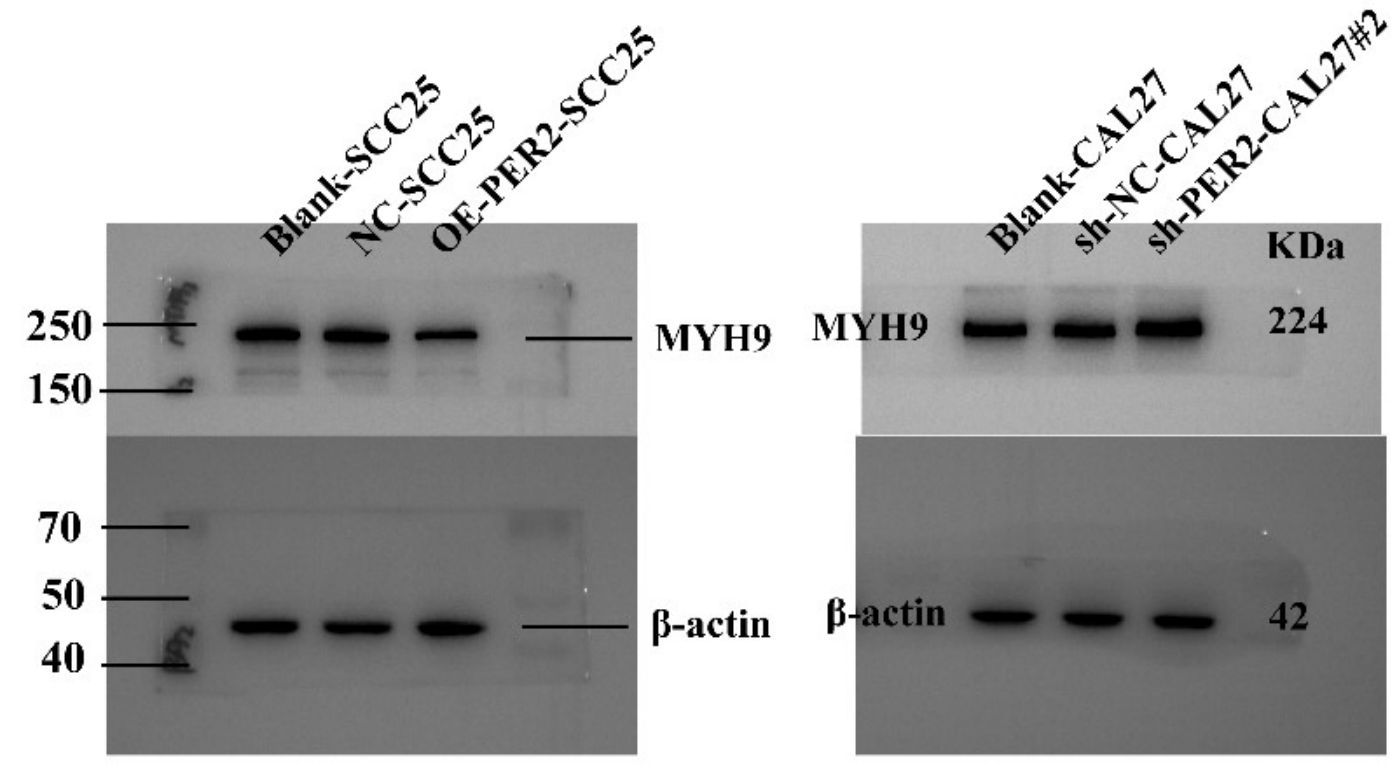

Supplementary Fig.3B

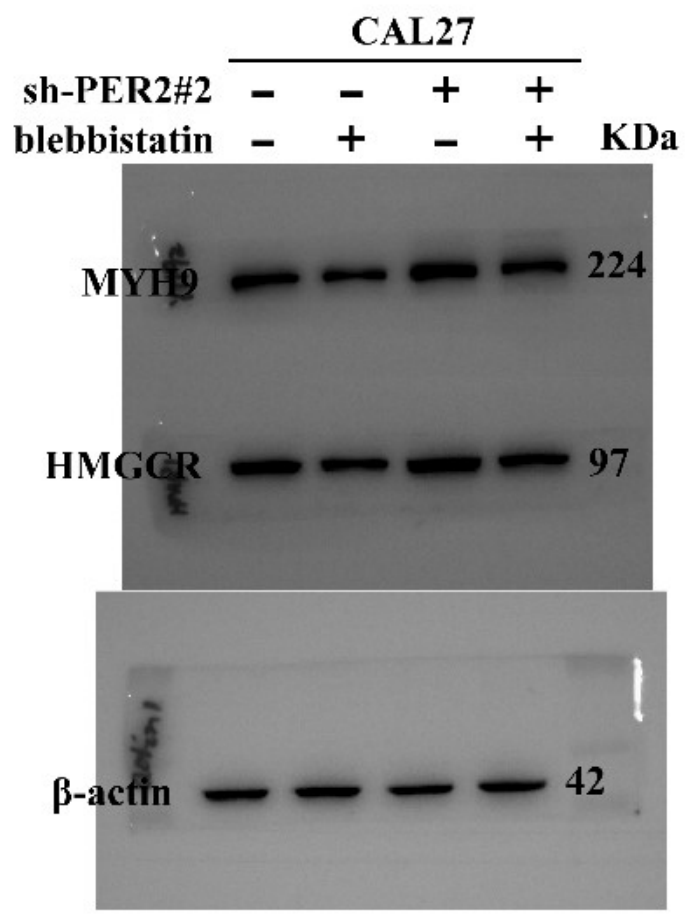

Supplementary Fig.3G

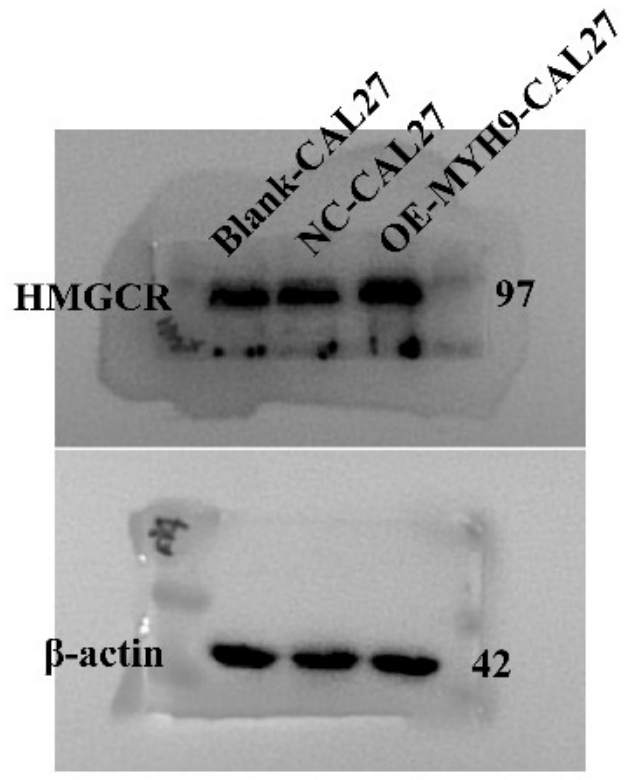

Supplementary Fig.3H

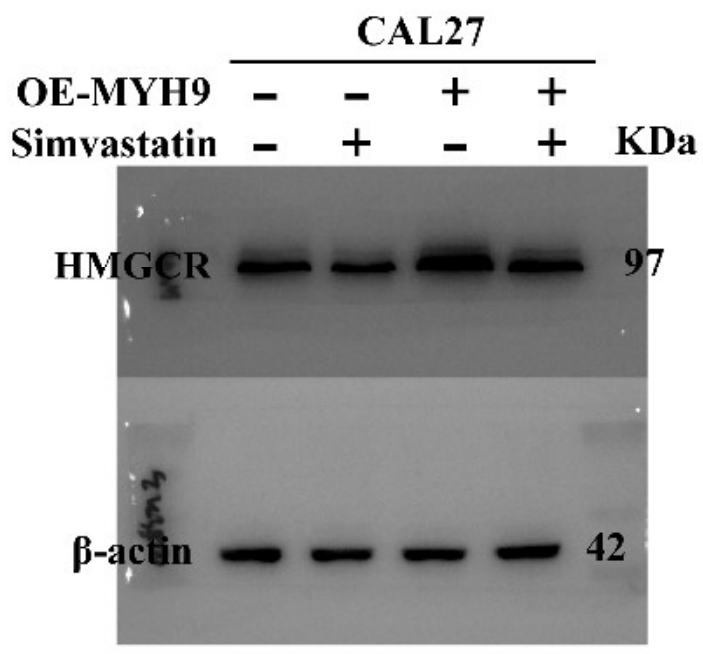

Supplementary Fig.4B

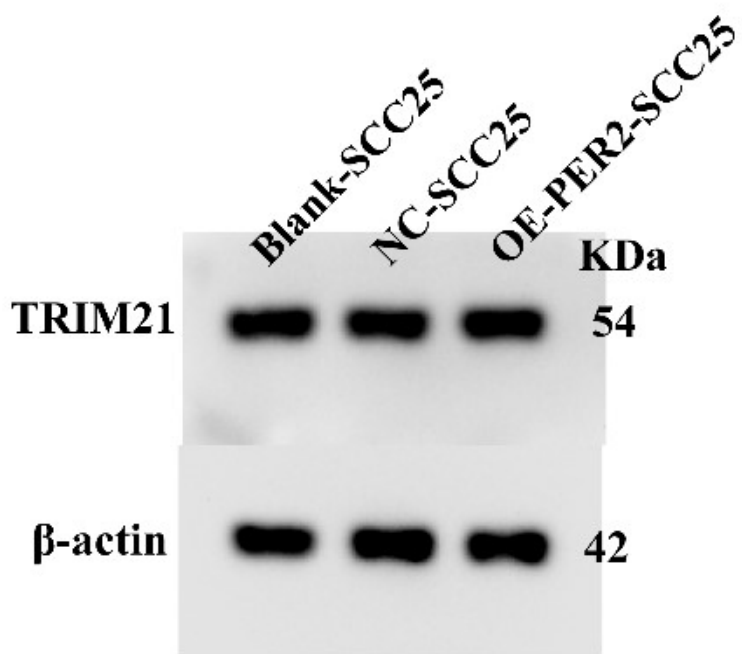

Supplementary Fig.4C

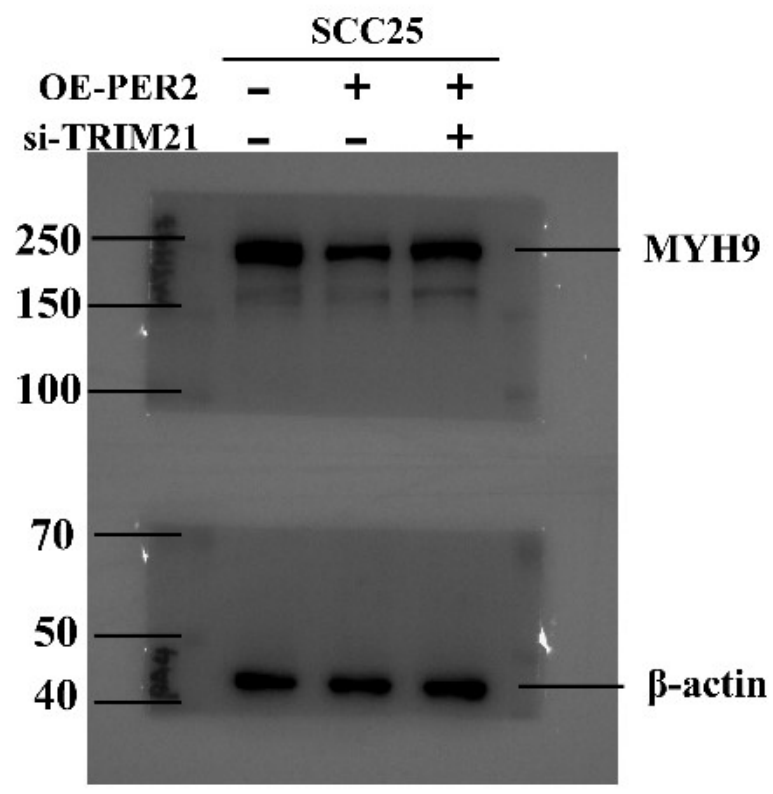

Supplementary Fig.4D

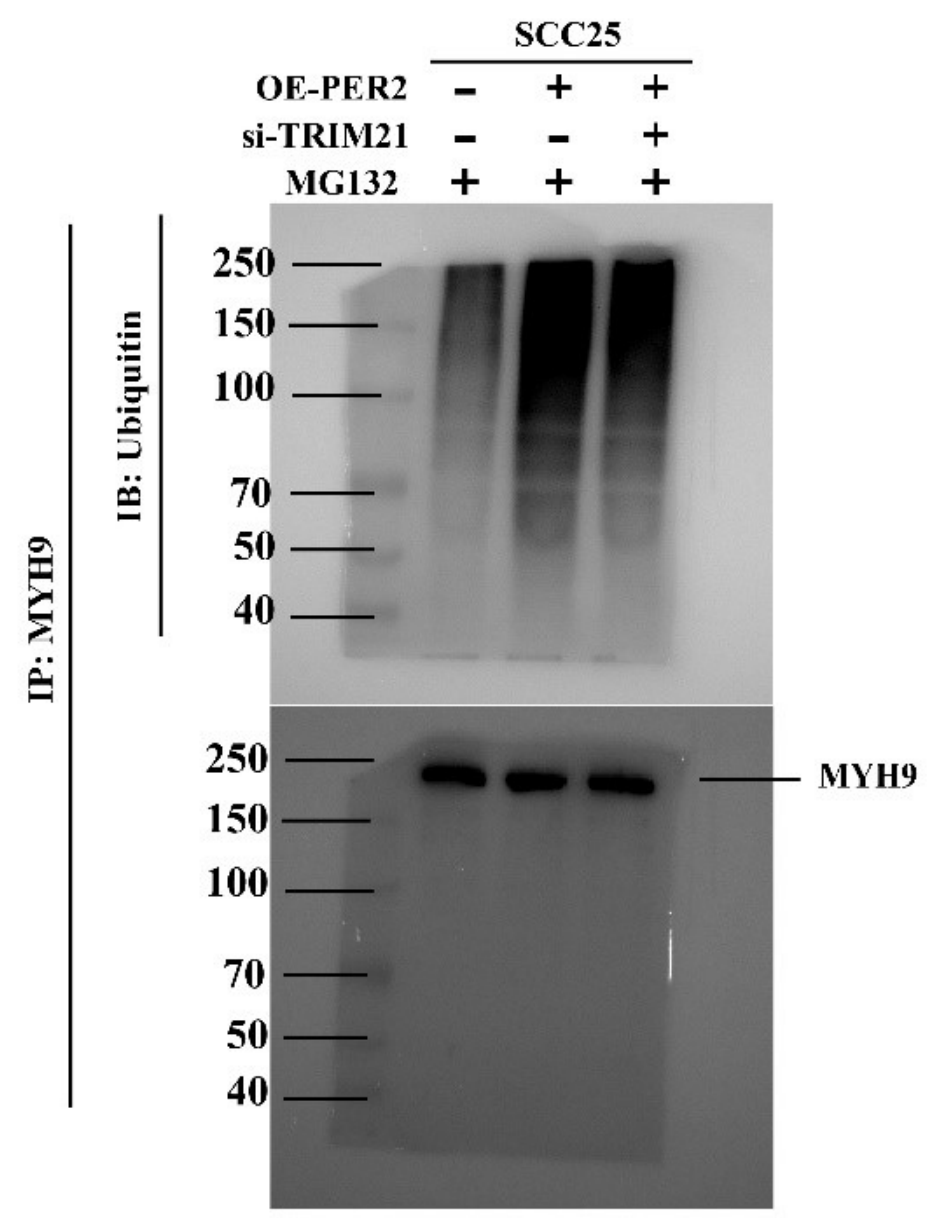

Supplementary Fig.4E

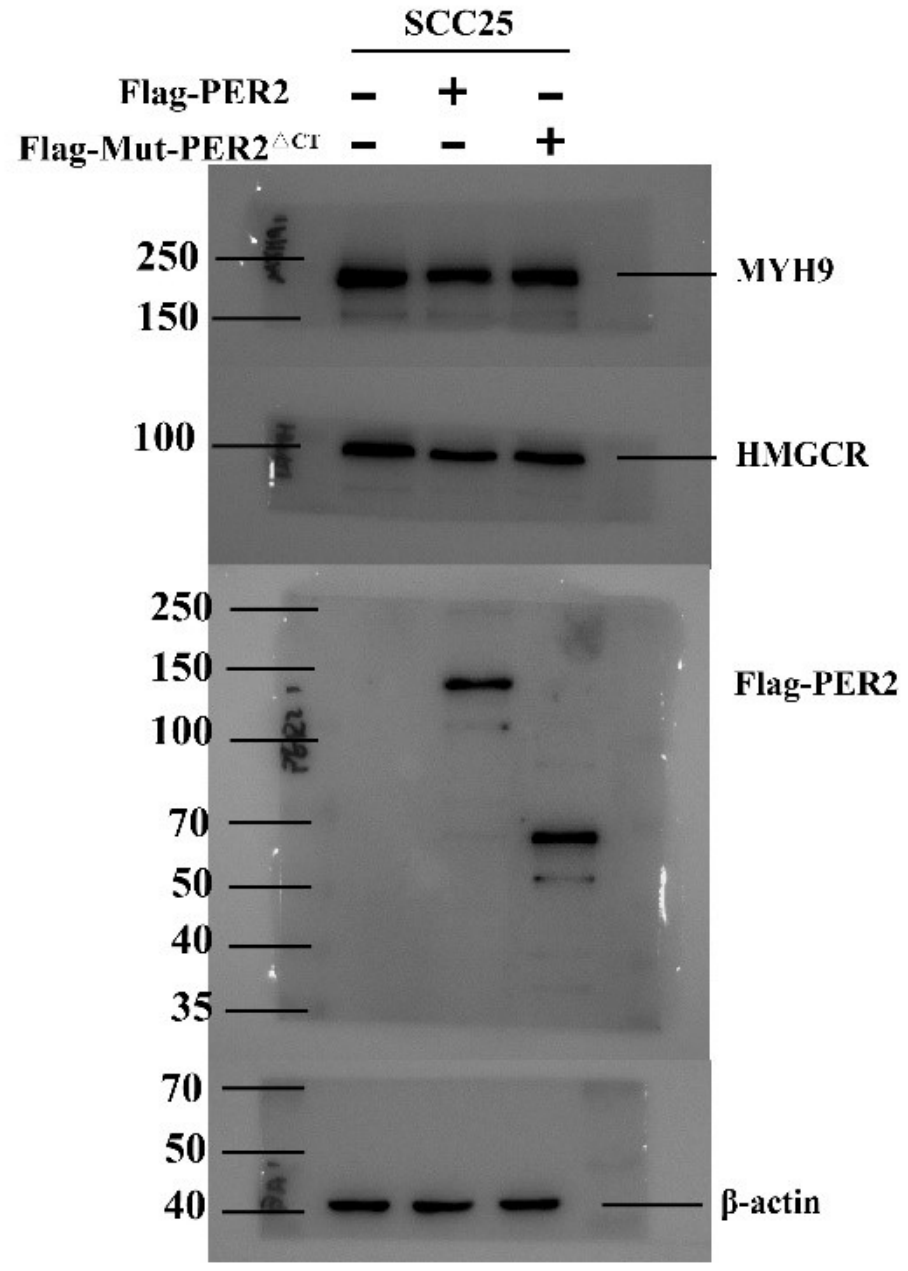

Supplementary Fig.4F

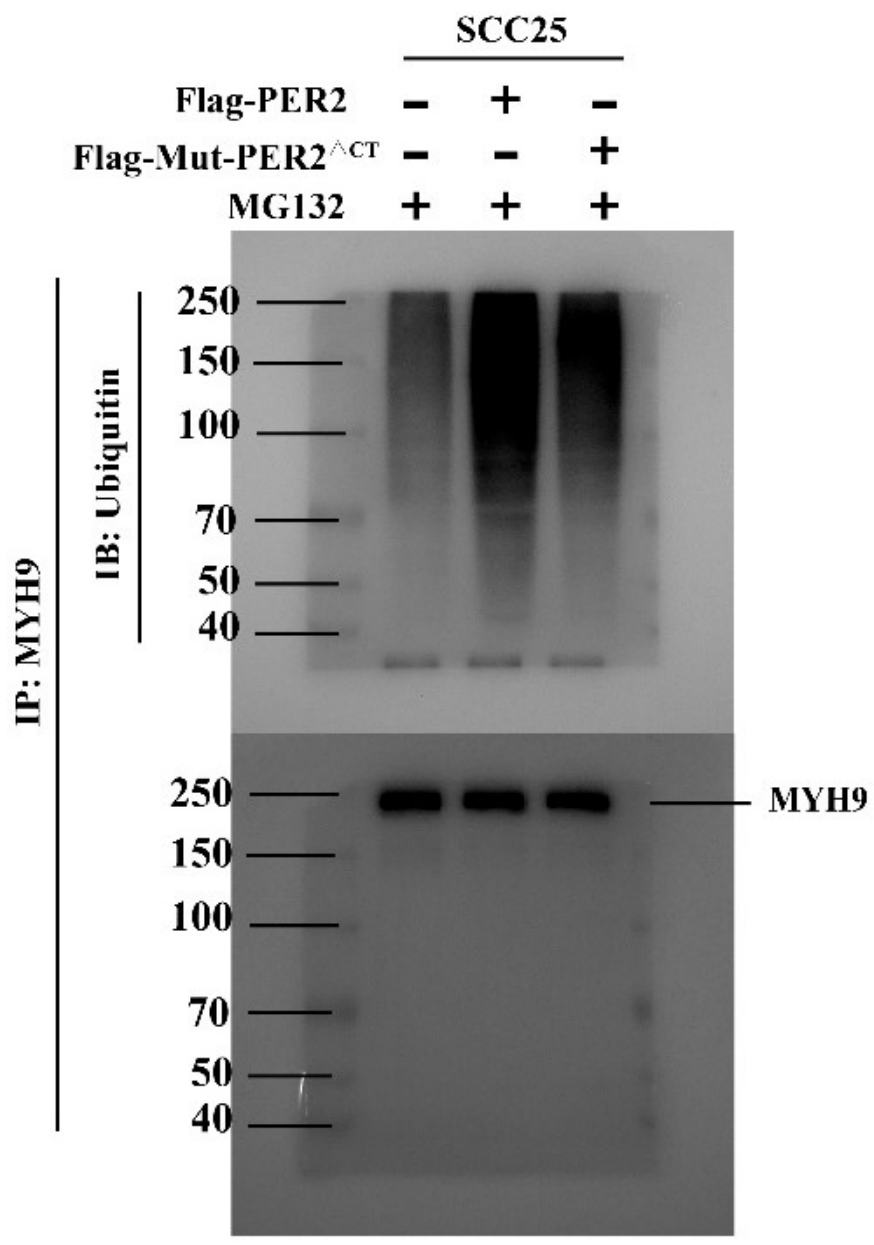

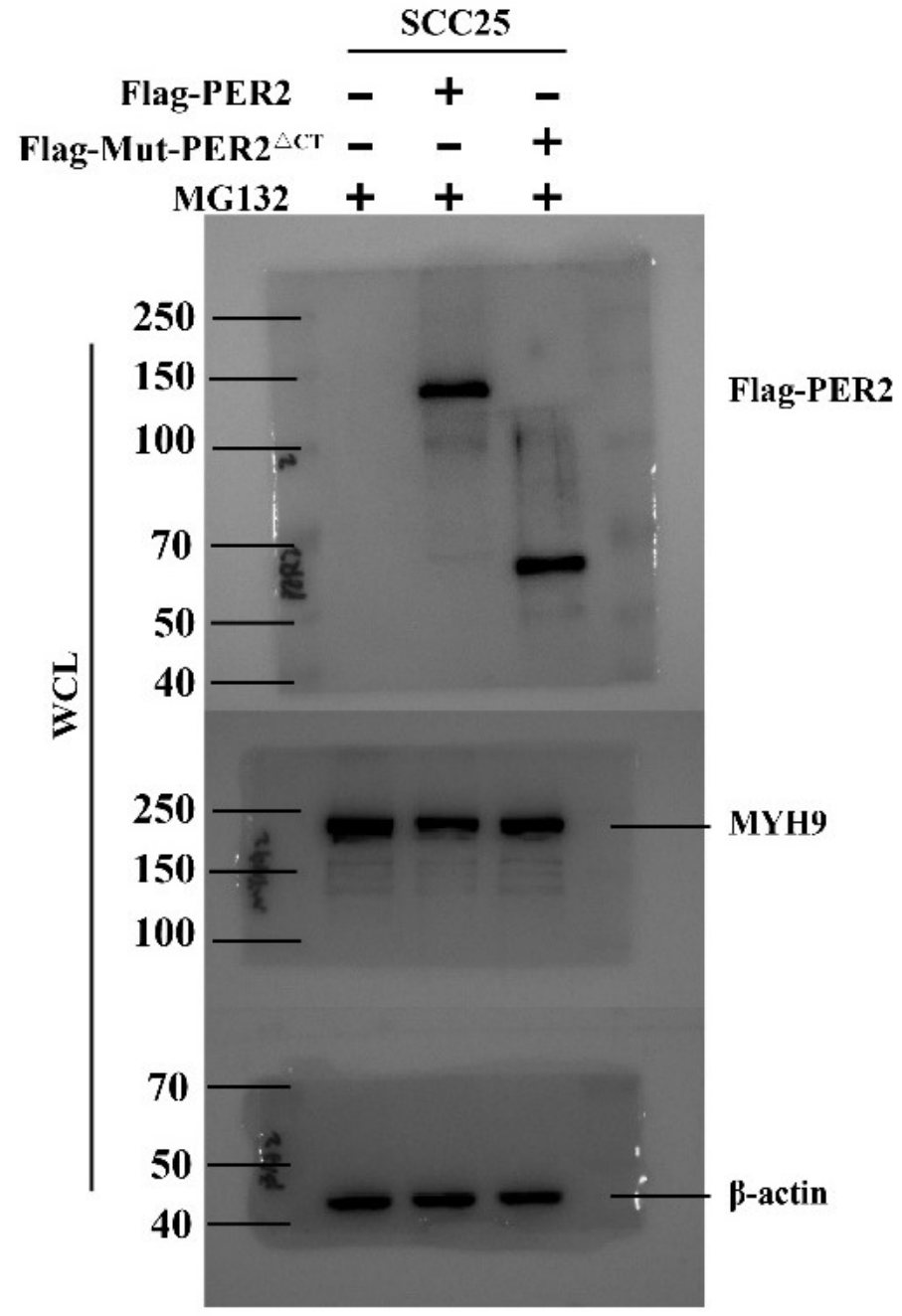

Supplement: Supplementary file 1 [file 41420_2026_3209_MOESM14_ESM.pdf]
