# Supplementary material for: PER2 reprograms intracellular cholesterol synthesis to inhibit oral squamous cell carcinoma and the chronotherapeutic efficacy of simvastatin
Source: Cell Death Discov. 2026 Jun 20;12:351. doi: 10.1038/s41420-026-03209-5 (PMC13529778; doi:10.1038/s41420-026-03209-5)
Supplement: Supplementary file 2 [file 41420_2026_3209_MOESM1_ESM.pdf]

Supplementary Material

Supplementary Figures 1-6 for

PER2 reprograms intracellular cholesterol synthesis to inhibit oral squamous cell carcinoma and the chronotherapeutic efficacy of simvastatin

Shilin Yin[1], Fang Yang[1,2], Zhiwei Zhang[1], Mingyuan Liu[1], Hong Tang[1], Kai Yang[1*]

[1]Department of Oral and Maxillofacial Surgery, The First Affiliated Hospital of Chongqing Medical University, Chongqing 400016, China.

[2] Department of Stomatology, Chongqing General Hospital, Chongqing University, Chongqing 400014, China.

Corresponding author: Kai Yang

Department of Oral and Maxillofacial Surgery, The First Affiliated Hospital of Chongqing Medical University, No.1, Youyi Road, Yuzhong District, Chongqing 400016, China

E-mail: cqfyyk@hotmail.com

Telephone: +86-23-89012569

Fig. S1

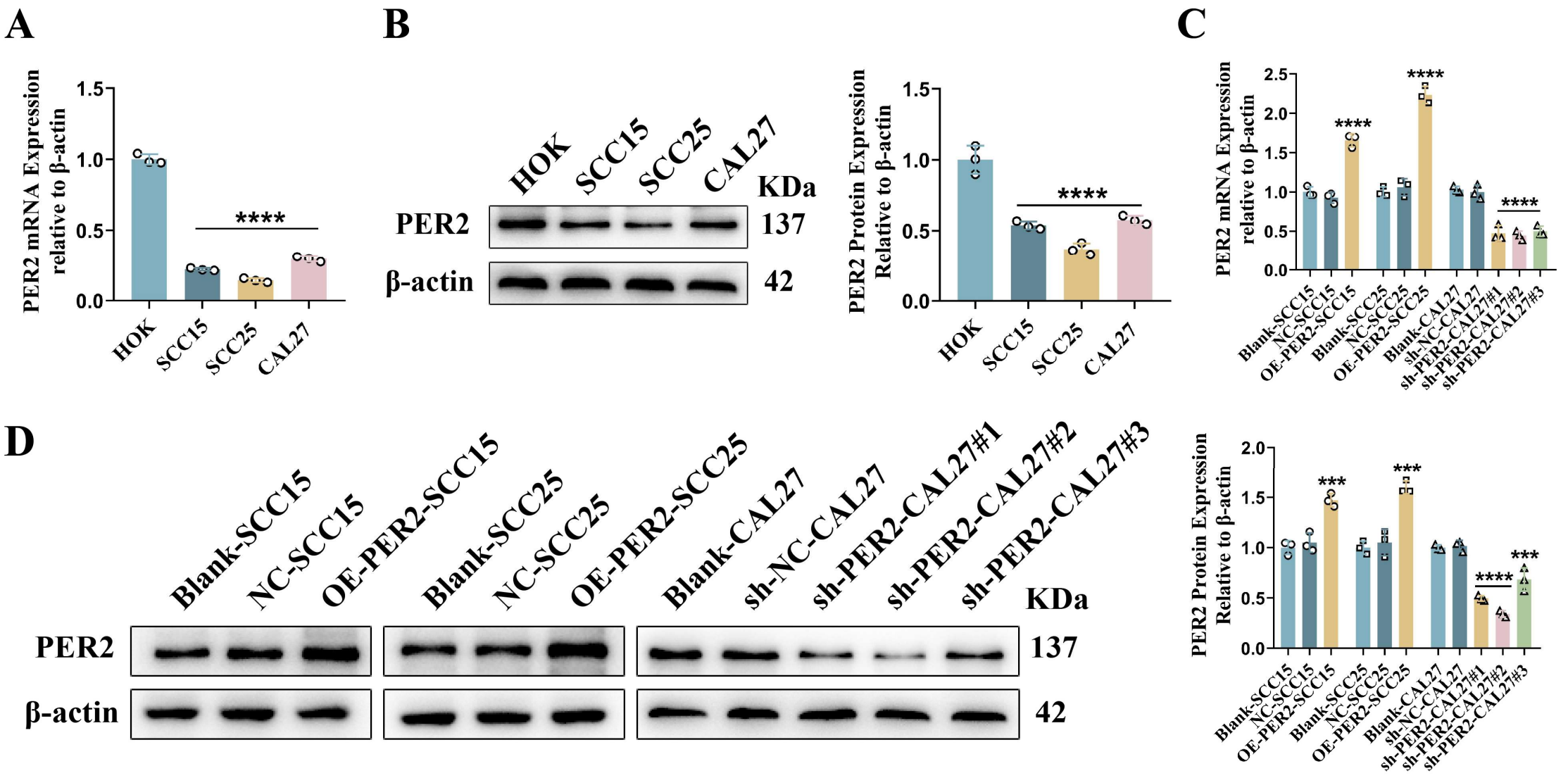

Fig. S1 Establishment of OSCC cell lines with stable PER2 overexpression or silencing. **A.** *PER2* mRNA expression in three OSCC cell lines (SCC15, SCC25, and CAL27) was detected by RT-qPCR**; B.** PER2 protein expression in the three OSCC cell lines was analyzed by Western blotting; **C**. RT-qPCR analysis revealed that compared with control groups, *PER2* mRNA expression levels in OE-PER2-SCC15 and OE-PER2-SCC25 cells were significantly increased to 1.66±0.08-fold and 2.23±0.11-fold, respectively. Among the constructed sh-PER2-CAL27#1, sh-PER2-CAL27#2, and sh-PER2-CAL27#3 cells, *PER2* was successfully silenced, with sh-PER2-CAL27#2 showing the highest silencing efficiency (61.4%±5.66% reduction at mRNA level); **D.** Western blotting analysis demonstrated that compared with control groups, PER2 protein expression in OE-PER2-SCC15 and OE-PER2-SCC25 cells was significantly increased to 1.49±0.04-fold and 1.68±0.09-fold, respectively. Among the sh-PER2-CAL27 cell lines, sh-PER2-CAL27#2 showed the highest silencing efficiency (67.16%±2.9% reduction at protein level). All data represent three independent experiments. Data are presented as means ± SD ($n \geq 3$). **P* < 0.05; ***P* < 0.01; ****P* < 0.001; *****P* < 0.0001.

Fig. S2

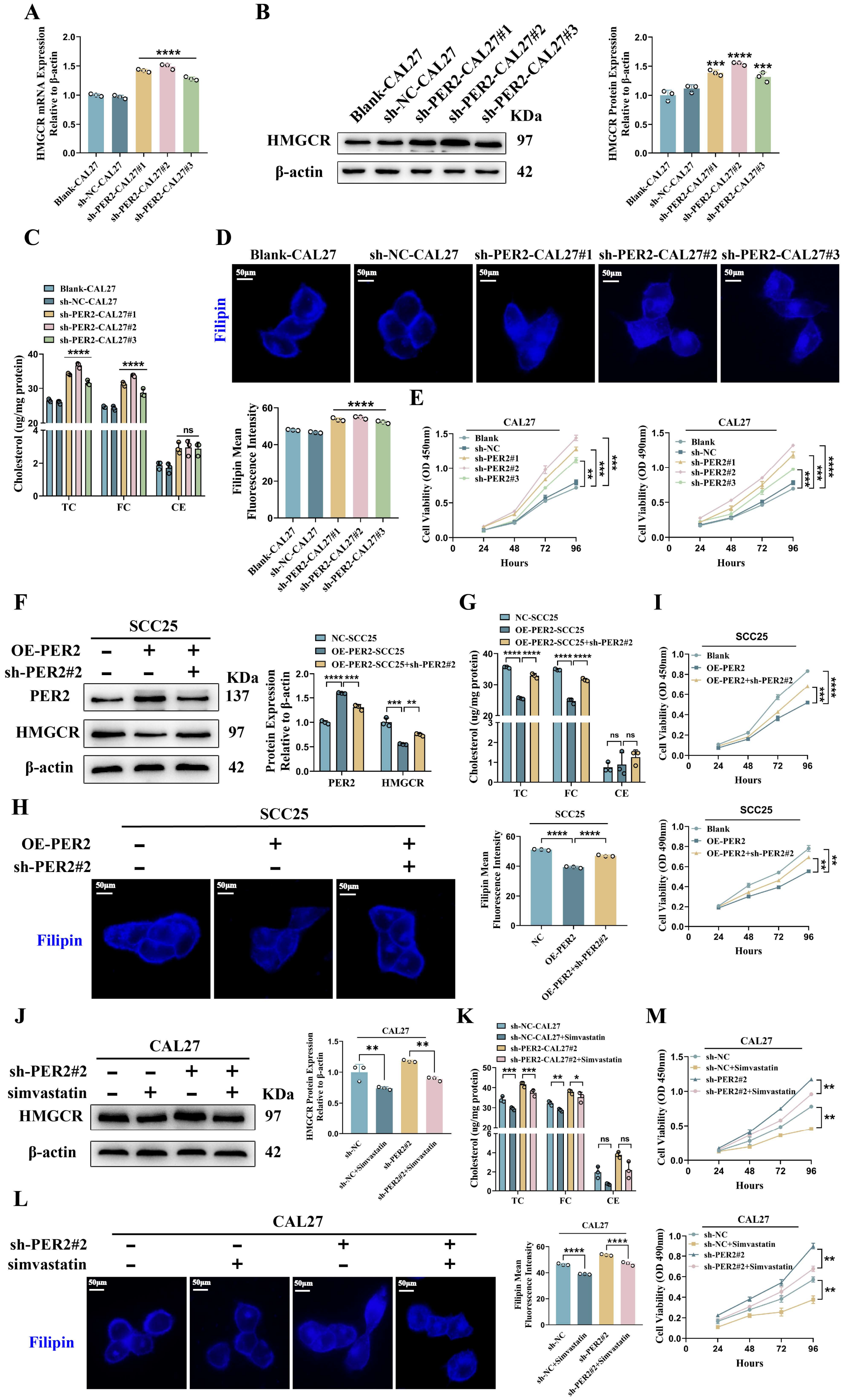

Fig. S2 PER2 suppresses OSCC proliferation by inhibiting HMGCR expression and

reducing intracellular cholesterol synthesis.

A. RT-qPCR analysis revealed significantly increased *HMGCR* mRNA levels in sh-PER2-CAL27#1, sh-PER2-CAL27#2, and sh-PER2-CAL27#3 cells; **B**. Western blotting demonstrated significantly elevated HMGCR protein expression in sh-PER2-CAL27#1, sh-PER2-CAL27#2, and sh-PER2-CAL27#3 cells; **C.** Cholesterol assays showed significantly increased TC and FC levels, but no significant difference in CE in sh-PER2-CAL27#1, sh-PER2-CAL27#2, and sh-PER2-CAL27#3 cells; **D**. Filipin staining confirmed significantly increased FC levels in PER2-silenced OSCC cells; **E.** CCK-8 and MTT assays indicated that PER2 silencing promoted OSCC cell proliferation; **F.** Western blotting results showing that HMGCR expression, which was decreased in OE-PER2-SCC25 cells, was significantly rescued in OE-PER2-SCC25+sh-PER2#2 cells. **G**. Cholesterol measurements revealing that reduced TC and FC levels in OE-PER2-SCC25 cells were significantly rescued in OE-PER2-SCC25+sh-PER2#2 cells, with no significant change in CE. **H**. Filipin staining demonstrating that after silencing of *PER2* in OE-PER2-SCC25 cells, FC levels were significantly rescued. **I.** CCK-8 and MTT assays demonstrating that the decreased proliferation in OE-PER2-SCC25 cells was significantly rescued in OE-PER2-SCC25+shPER2#2 cells. **J.** Western blotting analysis showing that compared to sh-PER2-CAL27#2 cells, HMGCR expression was significantly rescued in sh-PER2-CAL27#2+simvastatin cells. **K.** Cholesterol assays revealing that simvastatin treatment significantly reversed the increases in TC and FC levels in sh-PER2-CAL27#2 cells, whereas CE levels remained unchanged. **L.** Filipin staining confirming that simvastatin treatment significantly decreased the elevated FC levels in sh-PER2-CAL27#2 cells. **M**. CCK-8 and MTT assays indicating that simvastatin treatment significantly attenuated the enhanced proliferation of sh-PER2-CAL27#2 cells. All data represent three independent experiments. Data are presented as means ± SD ($n \geq 3$). *$P < 0.05$; **$P < 0.01$; ***$P < 0.001$; ****$P < 0.0001$. TC, total cholesterol; FC, free cholesterol; CE, cholesteryl esters

Fig. S3

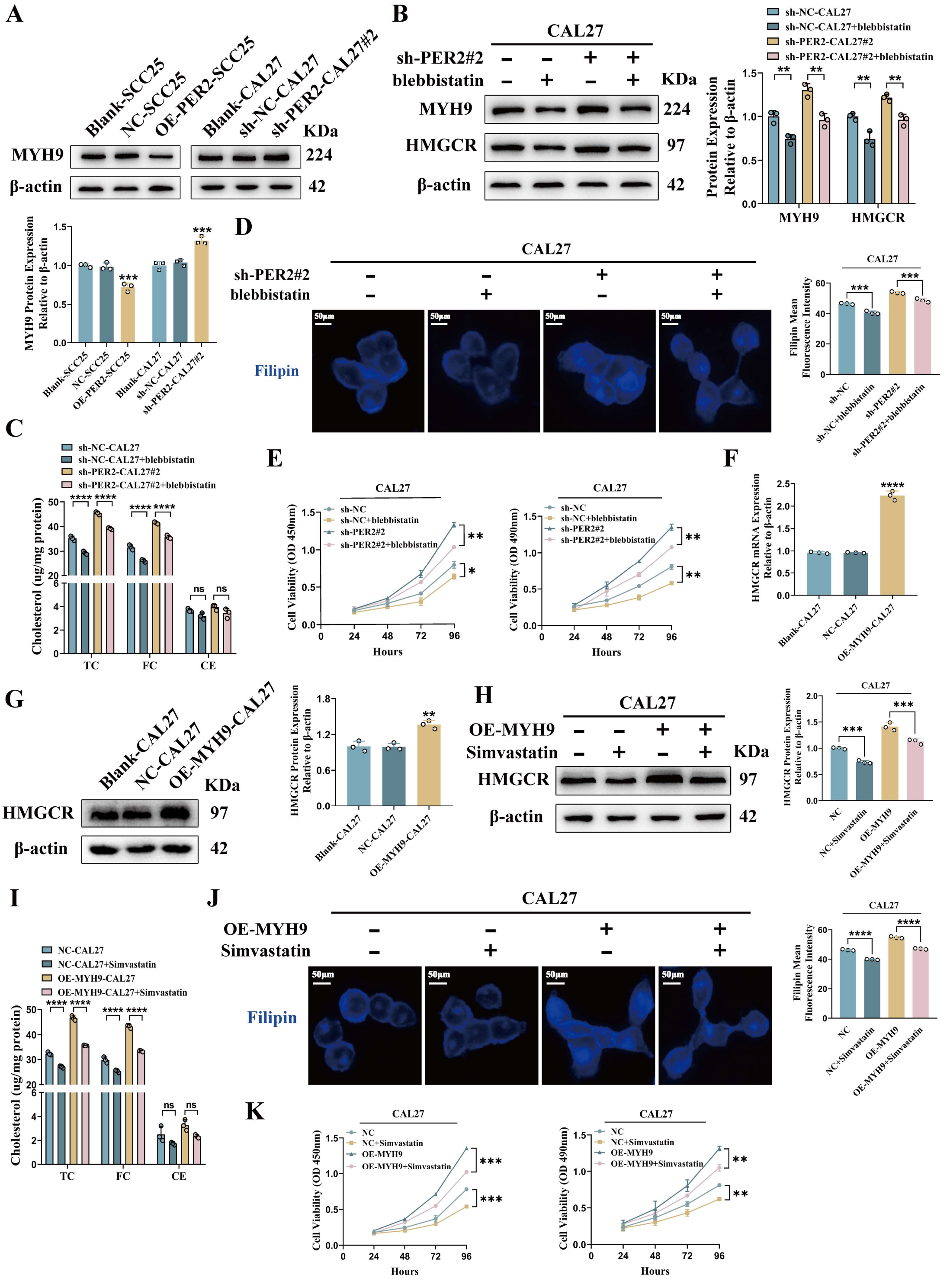

Fig. S3 PER2 suppresses cholesterol synthesis and proliferation in OSCC cells by inhibiting MYH9.

A. Western blotting analysis revealed significantly decreased MYH9 expression in OE-PER2-SCC25 cells and significantly increased MYH9 expression in sh-PER2-CAL27#2 cells; **B.** Western blotting demonstrated that blebbistatin (MYH9 inhibitor)

treatment significantly rescued the elevated HMGCR expression in sh-PER2-CAL27#2 cells; **C.** Cholesterol assays showed that blebbistatin treatment significantly reduced the increased TC and FC levels in sh-PER2-CAL27#2 cells, with no significant change in CE; **D.** Filipin staining confirmed that blebbistatin treatment significantly decreased the elevated FC levels in sh-PER2-CAL27#2 cells; **E.** CCK-8 and MTT assays indicated that blebbistatin treatment significantly suppressed the enhanced proliferation of sh-PER2-CAL27#2 cells; **F.** RT-qPCR analysis showed that HMGCR mRNA expression was significantly increased in OE-MYH9-CAL27 cells; **G**. Western blotting analysis showed that HMGCR protein expression was significantly increased in OE-MYH9-CAL27 cells; **H.** Western blotting analysis demonstrated that simvastatin treatment significantly rescued the elevated HMGCR expression in OE-MYH9-CAL27 cells; **I.** Cholesterol measurement revealed that simvastatin treatment significantly reduced the increased TC and FC levels in OE-MYH9-CAL27 cells, while CE remained unchanged; **J**. Filipin staining demonstrated that simvastatin treatment significantly decreased the elevated FC levels in OE-MYH9-CAL27 cells; **K.** CCK-8 and MTT assays showed that simvastatin treatment significantly inhibited the increased proliferation of OE-MYH9-CAL27 cells. All data represent three independent experiments. Data are presented as means ± SD ($n \geq 3$). *$P < 0.05$; **$P < 0.01$; ***$P < 0.001$; ****$P < 0.0001$.

Fig. S4

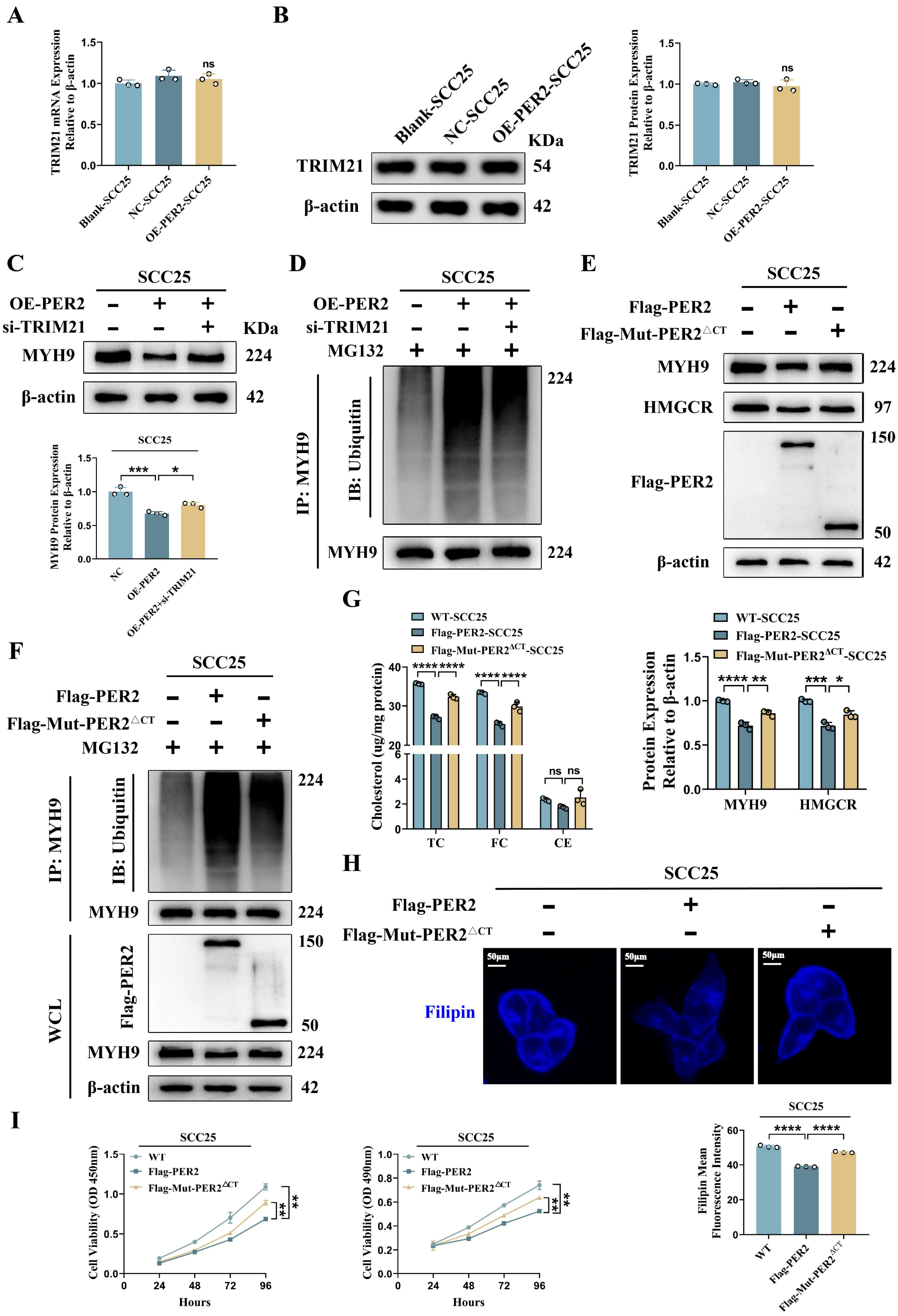

Fig. S4 PER2 forms a PER2–TRIM21–MYH9 complex to regulate cholesterol synthesis and proliferation in OSCC cells.

A. RT-qPCR analysis showed no significant changes in *TRIM21* mRNA expression

levels in OE-PER2-SCC25 cells; **B**. Western blotting detected no significant alterations in TRIM21 protein expression in OE-PER2-SCC25 cells; **C**. Western blotting analysis demonstrated that the decreased MYH9 expression in OE-PER2-SCC25 cells, was significantly rescued in OE-PER2-SCC25+si-TRIM21 cells; **D**. Ubiquitination assays revealed significantly rescued MYH9 ubiquitination levels in OE-PER2-SCC25+si-TRIM21 cells; **E**. Western blotting results indicated that the reduced MYH9 and HMGCR protein expression in Flag-PER2-SCC25 cells was significantly rescued in Flag-Mut-PER2$^{\Delta CT}$-SCC25 cells; **F.** Ubiquitination analysis showed significantly rescued MYH9 ubiquitination levels in Flag-Mut-PER2$^{\Delta CT}$-SCC25 cells; **G.** Cholesterol measurement demonstrated that compared with WT-SCC25 cells, the decreased TC and FC levels in Flag-PER2-SCC25 cells were significantly rescued in Flag-Mut-PER2$^{\Delta CT}$-SCC25 cells; **H**. Filipin staining revealed significantly rescued FC levels in Flag-Mut-PER2$^{\Delta CT}$-SCC25 cells compared to Flag-PER2-SCC25 cells; **I**. CCK-8 and MTT assays showed that the inhibited cell proliferation in Flag-PER2-SCC25 cells was significantly rescued in Flag-Mut-PER2$^{\Delta CT}$-SCC25 cells. All data represent three independent experiments. Data are presented as means ± SD ($n \geq 3$). *$P < 0.05$; **$P < 0.01$; ***$P < 0.001$; ****$P < 0.0001$.

Fig. S5

A

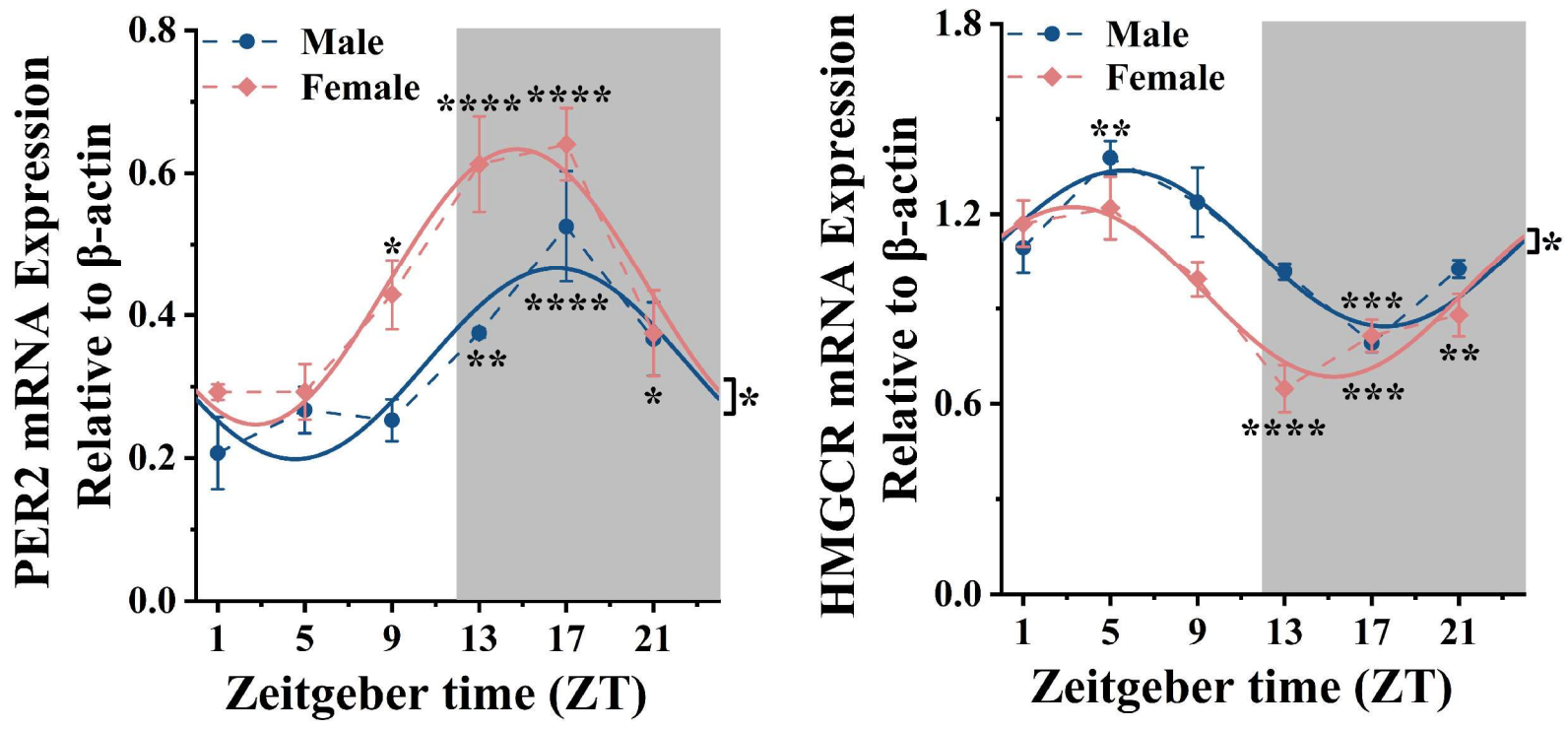

Fig. S5 Circadian rhythm characteristics and sex differences of *PER2* and *HMGCR* mRNA expression in OSCC

A. RT-qPCR analysis of *PER2* and *HMGCR* mRNA expression in OSCC tissues from male and female mice at six different time points over 24 hours, followed by cosine curve fitting and analysis, revealed significant circadian rhythms (one-way ANOVA: $P < 0.05$; cosinor analysis: $P < 0.05$). In OSCC tissues, male mice demonstrated significantly lower mesors of *PER2* mRNA expression but higher mesors of *HMGCR* mRNA expression compared to females, whereas no significant differences were observed in the amplitude of either *PER2* or *HMGCR* mRNA expression. Notably, male mice exhibited delayed acrophases in both *PER2* and *HMGCR* mRNA expression compared to females, with *PER2* delayed by 1.82 h (ZT16.55 vs. ZT14.73) and *HMGCR* delayed by 2.34 h (ZT5.60 vs. ZT3.26). In all figures, solid lines represent cosine-fitted curves while dashed lines connect actual mean values at six different time points within 24 hours. All data represent three independent experiments. Data are presented as means ± SD ($n \geq 3$). *$P < 0.05$; **$P < 0.01$; ***$P < 0.001$; ****$P < 0.0001$.

Fig.S6

A

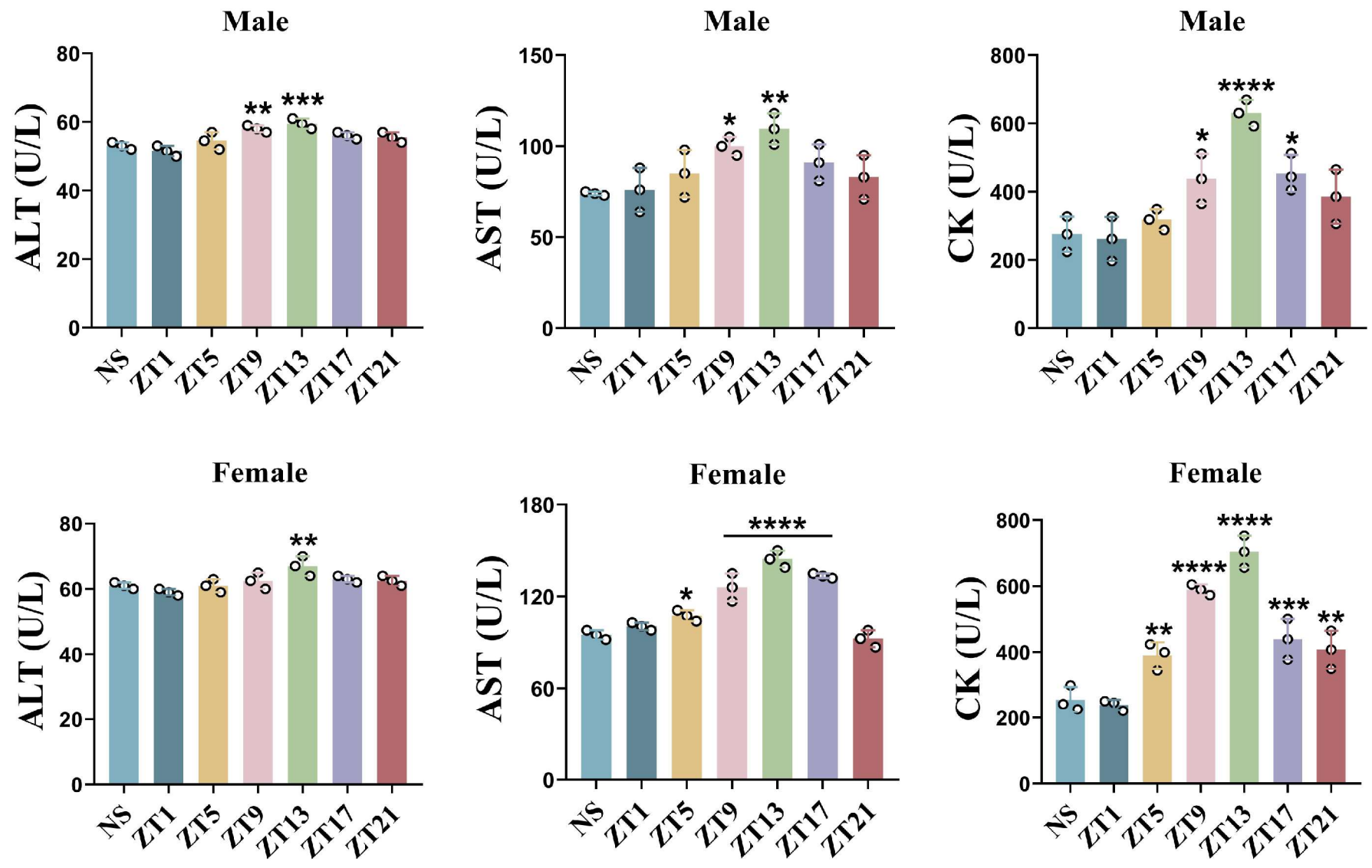

Fig. S6 Time-dependence and sex differences in adverse reactions of simvastatin in the treatment of OSCC.

A. Biochemical assessment of hepatotoxicity (serum ALT and AST levels) and myotoxicity (serum CK levels) in male and female mice at different treatment time points (compared with NS). All data represent three independent experiments. Data are presented as means ± SD (n ≥ 3). *$P < 0.05$; **$P < 0.01$; ***$P < 0.001$; ****$P < 0.0001$. ALT, Alanine Aminotransferase; AST, Aspartate Aminotransferase; CK, Creatine Kinase.
