# Supplementary material for: PER2 reprograms intracellular cholesterol synthesis to inhibit oral squamous cell carcinoma and the chronotherapeutic efficacy of simvastatin
Source: Cell Death Discov. 2026 Jun 20;12:351. doi: 10.1038/s41420-026-03209-5 (PMC13529778; doi:10.1038/s41420-026-03209-5)
Supplement: Supplementary file 3 [file 41420_2026_3209_MOESM11_ESM.docx]

**Supplementary Material**

**Supplementary Tables 10-14 for**

**PER2 reprograms intracellular cholesterol synthesis to inhibit oral squamous cell carcinoma and the chronotherapeutic efficacy of simvastatin**

Shilin Yin^1^, Fang Yang^1,2^, Zhiwei Zhang^1^, Mingyuan Liu^1^, Hong Tang^1^, Kai Yang^1^**^*^**

^1^Department of Oral and Maxillofacial Surgery, The First Affiliated Hospital of Chongqing Medical University, Chongqing 400016, China.

^2^ Department of Stomatology, Chongqing General Hospital, Chongqing University, Chongqing 400014, China.

**Corresponding author:** Kai Yang

Department of Oral and Maxillofacial Surgery, The First Affiliated Hospital of Chongqing Medical University, No.1, Youyi Road, Yuzhong District, Chongqing 400016, China

E-mail: [cqfyyk@hotmail.com](mailto:cqfyyk@hotmail.com)

Telephone: +86-23-89012569

**Table S10.** PER2 regulates the circadian rhythms of HMGCR expression, cholesterol, and cell proliferation in OSCC cells

| Detection | cell | Mesor | Amplitude | Acrophase  (ZT) | Peak value | Trough value | *P*-value |
| --- | --- | --- | --- | --- | --- | --- | --- |
| PER2 | NC-SCC25 | 0.69 | 0.40 | 15.88 | 1.10 | 0.29 | 0.006 |
|  | OE-PER2-SCC25 | **0.91*** | 0.41 | 14.61 | 1.32 | 0.50 | 0.028 |
| HMGCR | NC-SCC25 | 0.76 | 0.28 | 4.06 | 1.04 | 0.48 | 0.009 |
|  | OE-PER2-SCC25 | **0.63*** | 0.41 | **1.58*** | 1.04 | 0.22 | 0.003 |
| TC | NC-SCC25 | 36.24 | 7.96 | 5.45 | 44.20 | 28.29 | 0.005 |
|  | OE-PER2-SCC25 | **30.62**** | 6.58 | **2.60**** | 37.20 | 24.04 | 0.005 |
| FC | NC-SCC25 | 33.82 | 8.76 | 5.63 | 42.58 | 25.06 | 0.003 |
|  | OE-PER2-SCC25 | **28.69**** | 6.27 | **2.65**** | 34.96 | 22.43 | 0.007 |
| Cell Viability | NC-SCC25 | 0.72 | 0.21 | 8.26 | 0.93 | 0.50 | 0.015 |
|  | OE-PER2-SCC25 | **0.58*** | 0.24 | **4.19**** | 0.81 | 0.34 | 0.012 |
| HMGCR | sh-NC-CAL27 | 0.63 | 0.45 | 1.79 | 1.08 | 0.18 | 0.038 |
|  | sh-PER2-CAL27 | **0.91*** | 0.34 | **6.15*** | 1.25 | 0.57 | 0.026 |
|  | sh-PER2-CAL27+SIM | **0.68^#^** | 0.31 | 4.00 | 0.99 | 0.36 | 0.043 |
| TC | sh-NC-CAL27 | 36.62 | 3.18 | 2.21 | 39.80 | 33.45 | 0.023 |
|  | sh-PER2-CAL27 | **43.59***** | **6.98*** | **7.65**** | 50.57 | 36.62 | 0.011 |
|  | sh-PER2-CAL27+SIM | **37.49^##^** | 5.39 | 5.06 | 42.87 | 32.10 | 0.018 |
| FC | sh-NC-CAL27 | 33.94 | 3.61 | 2.46 | 37.55 | 30.32 | 0.024 |
|  | sh-PER2-CAL27 | **40.62***** | **7.04*** | **7.41**** | 47.66 | 33.58 | 0.005 |
|  | sh-PER2-CAL27+SIM | **34.81^###^** | 6.36 | **5.03^#^** | 41.17 | 28.45 | 0.004 |
| Cell Viability | sh-NC-CAL27 | 0.67 | 0.19 | 4.13 | 0.85 | 0.48 | 0.010 |
|  | sh-PER2-CAL27 | **0.80**** | 0.19 | **10.30***** | 1.00 | 0.61 | 0.007 |
|  | sh-PER2-CAL27+SIM | **0.70^#^** | 0.19 | **6.51^#^** | 0.89 | 0.51 | 0.021 |

**Note:** The circadian rhythm is described by the mesor, amplitude and acrophase. *P* < 0.05 indicating a circadian rhythm.

* Indicates a significant difference in mesor, amplitude, or acrophase compared with NC-SCC25 or sh-NC-CAL27; **P* < 0.05; ***P* < 0.01; ****P* < 0.001; *****P* < 0.0001

^#^ Indicates a significant difference in mesor, amplitude, or acrophase compared with sh-PER2-CAL27; ^#^*P* < 0.05; ^##^*P* < 0.01; ^###^*P* < 0.001; ^####^*P* < 0.0001

**Abbreviations:** TC, Total Cholesterol; FC, Free Cholesterol; SIM, Simvastatin.

**Table S11.** Circadian characteristics and sex differences in PER2 and HMGCR expression, intracellular cholesterol and cell proliferation *in vivo*.

| Detection | Sex | Tissue | Mesor | Amplitude | Acrophase  (ZT) | Peak value | Trough value | *P*-value |
| --- | --- | --- | --- | --- | --- | --- | --- | --- |
| PER2 | Male | OSCC | 0.83 | 0.34 | 17.58 | 1.17 | 0.48 | 0.007 |
|  | Female |  | 0.90 | 0.33 | 15.40 | 1.24 | 0.57 | 0.021 |
| HMGCR | Male | OSCC | 1.11 | 0.16 | 6.71 | 1.27 | 0.94 | 0.030 |
|  | Female |  | **0.99*** | 0.18 | 4.01 | 1.17 | 0.80 | 0.042 |
| TC | Male | OSCC | 45.62 | 7.28 | 7.79 | 52.90 | 38.33 | 0.018 |
|  | Female |  | **41.60*** | 4.89 | **4.51*** | 46.49 | 36.71 | 0.019 |
| FC | Male | OSCC | 43.02 | 6.72 | 7.92 | 49.74 | 36.29 | 0.014 |
|  | Female |  | **39.14*** | 4.11 | **4.76*** | 43.25 | 35.03 | 0.016 |
| Ki-67 | Male | OSCC | 50.43 | 17.61 | 8.31 | 68.04 | 32.82 | 0.001 |
|  | Female |  | **43.37*** | 16.84 | **2.60***** | 60.21 | 26.54 | 0.023 |

**Note:** The circadian rhythm is described by the mesor, amplitude and acrophase. *P* < 0.05 indicating a circadian rhythm.

* Indicates a significant difference in mesor, amplitude, or acrophase between males and females; **P* < 0.05; ***P* < 0.01; ****P* < 0.001; *****P* < 0.0001

**Abbreviations:** TC, Total Cholesterol; FC, Free Cholesterol.

**Table S12.** Circadian rhythm characteristics of tumor volume and weight in male and female C57BL/6J mice

| **Detection** | **Sex** | **Mesor** | **Amplitude** | **Acrophase (ZT)** | **Peak value** | **Trough value** | ***P*-value** |
| --- | --- | --- | --- | --- | --- | --- | --- |
| Tumor Volume | Male | 810.88 | 153.26 | 8.49 | 964.14 | 657.62 | 0.015 |
|  | Female | **719.08*** | 113.65 | **2.31**** | 832.73 | 605.43 | 0.011 |
| Tumor Weight | Male | 0.52 | 0.15 | 8.60 | 0.67 | 0.37 | 0.011 |
|  | Female | **0.42*** | 0.12 | **2.90**** | 0.54 | 0.31 | 0.028 |

**Note:** The circadian rhythm is described by the mesor, amplitude and acrophase. *P* < 0.05 indicating a circadian rhythm.

* Indicates a significant difference in mesor, amplitude, or acrophase between males and females; **P* < 0.05; ***P* < 0.01; ****P* < 0.001; *****P* < 0.0001

**Table S13.** Circadian rhythm characteristics and sex differences of *PER2* and *HMGCR* mRNA expression in OSCC

| Detection | Sex | Tissue | Mesor | Amplitude | Acrophase  (ZT) | Peak value | Trough value | *P*-value |
| --- | --- | --- | --- | --- | --- | --- | --- | --- |
| PER2 mRNA | Male | OSCC | 0.33 | 0.13 | 16.55 | 0.47 | 0.20 | 0.038 |
|  | Female |  | **0.44*** | 0.19 | 14.73 | 0.63 | 0.25 | 0.005 |
| HMGCR mRNA | Male | OSCC | 1.09 | 0.25 | 5.60 | 1.34 | 0.84 | 0.014 |
|  | Female |  | **0.95*** | 0.27 | 3.26 | 1.22 | 0.69 | 0.012 |

**Note:** The circadian rhythm is described by the mesor, amplitude and acrophase. *P* < 0.05 indicating a circadian rhythm.

* Indicates a significant difference in mesor, amplitude, or acrophase between males and females; **P* < 0.05; ***P* < 0.01; ****P* < 0.001; *****P* < 0.0001

**Table S14.** Tumor volume, weight, cellular proliferation, and adverse effect indicators in male and female C57BL/6J mice following 28-day grouped simvastatin treatment administered at six different circadian time points across 24 hours

| Detection | Sex | NS | ZT1 | ZT5 | ZT9 | ZT13 | ZT17 | ZT21 |
| --- | --- | --- | --- | --- | --- | --- | --- | --- |
| Tumor Volume | Male | 1058.29±120.97 | 555.99±154.49 | 476.98±121.08 | 633.17±143.28 | 658.05±120.89 | 826.24±78.85 | 736.68±147.00 |
|  | Female | 905.68±118.64 | 592.43±111.70 | 481.35±92.89 | 457.21±98.89 | 613.41±85.03 | 632.00±92.03 | 669.86±85.88 |
| Tumor Weight | Male | 0.81±0.10 | 0.44±0.04 | 0.40±0.07 | 0.52±0.03 | 0.58±0.04 | 0.66±0.02 | 0.63±0.03 |
|  | Female | 0.78±0.06 | 0.50±0.05 | 0.36±0.07 | 0.34±0.03 | 0.52±0.06 | 0.59±0.08 | 0.62±0.05 |
| Ki-67 | Male | 78.40±7.19 | 36.15±6.67 | 31.91±8.96 | 53.49±5.85 | 56.89±6.23 | 65.57±4.83 | 63.53±8.13 |
|  | Female | 68.92±5.83 | 43.99±7.83 | 31.61±3.16 | 30.30±4.56 | 46.03±6.01 | 53.07±4.57 | 54.99±9.95 |
| ALT | Male | 53.00±1.00 | 51.50±1.50 | 54.50±2.50 | 58.00±1.00 | 59.50±1.50 | 56.00±1.00 | 55.50±1.50 |
|  | Female | 61.00±1.00 | 59.00±1.00 | 61.00±2.00 | 62.50±2.50 | 67.00±3.00 | 63.00±1.00 | 62.50±1.50 |
| AST | Male | 74.00±1.00 | 76.00±12.00 | 85.00±13.00 | **100.00±5.00*** | **109.50±8.50*** | 91.00±10.00 | 83.00±12.00 |
|  | Female | 95.00±3.00 | 100.50±2.50 | 107.50±3.50 | **126.00±9.00*** | **144.50±5.50*** | **133.50±1.50*** | 92.50±5.50 |
| CK | Male | 275.50±51.50 | 261.50±64.50 | 318.50±30.50 | 438.00±73.00 | 630.50±38.50 | 453.50±54.20 | 385.50±79.50 |
|  | Female | 255.50±38.56 | 239.33±16.07 | 389.50±40.44 | 589.17±16.27 | **704.50±48.50*** | 439.50±61.50 | 407.50±57.50 |

**Note:** Data are expressed as mean ± SD.

**Normal reference ranges for C57BL/6J mice:** **ALT:** Male: 46-70 U/L | Female: 42-73 U/L; **AST:** Male: 55-91 U/L | Female: 51-122 U/L; **CK:** Male: 209-635 U/L | Female: 105-649 U/L

*Values marked with asterisk indicate levels above the normal reference range.

**Abbreviation:** NS, Normal Saline; ALT, Alanine Aminotransferase; AST, Aspartate Aminotransferase; CK, Creatine Kinase
