# Supplementary material for: PER2 reprograms intracellular cholesterol synthesis to inhibit oral squamous cell carcinoma and the chronotherapeutic efficacy of simvastatin
Source: Cell Death Discov. 2026 Jun 20;12:351. doi: 10.1038/s41420-026-03209-5 (PMC13529778; doi:10.1038/s41420-026-03209-5)
Supplement: Supplementary file 4 [file 41420_2026_3209_MOESM13_ESM.docx]

**Supplementary Material**

**Supplementary Tables 16-21 for**

**PER2 reprograms intracellular cholesterol synthesis to inhibit oral squamous cell carcinoma and the chronotherapeutic efficacy of simvastatin**

Shilin Yin^1^, Fang Yang^1,2^, Zhiwei Zhang^1^, Mingyuan Liu^1^, Hong Tang^1^, Kai Yang^1^**^*^**

^1^Department of Oral and Maxillofacial Surgery, The First Affiliated Hospital of Chongqing Medical University, Chongqing 400016, China.

^2^ Department of Stomatology, Chongqing General Hospital, Chongqing University, Chongqing 400014, China.

**Corresponding author:** Kai Yang

Department of Oral and Maxillofacial Surgery, The First Affiliated Hospital of Chongqing Medical University, No.1, Youyi Road, Yuzhong District, Chongqing 400016, China

E-mail: [cqfyyk@hotmail.com](mailto:cqfyyk@hotmail.com)

Telephone: +86-23-89012569

**Table S16.** Primer sequences for catching PER2 in constructing PER2 overexpression lentivirus.

| **ID** | **Sequence (5’ to 3’)** |
| --- | --- |
| PER2-F | AGGTCGACTCTAGAGGATCCCGCCACCATGAATGGATACGCGGAATTTCCGCCC |
| PER2-R | ACCGTAAGTTATGTGCTAGCTTACGTCTGCTCTTCGATCCTGTGATTC |

**Table S17.** Target sequence for sh-PER2

| **ID** | **Accession** | **Target Seq** |
| --- | --- | --- |
| sh-PER2#1 | NM_022817 | gcCAGAGTCCAGATACCTTTA |
| sh-PER2#2 | NM_022817 | ccACGAGAATGAAATCCGCTA |
| sh-PER2#3 | NM_022817 | ccAAATTGTTTGTTCCAGGAT |

**Table S18.** Primer sequences for deletion mutations in each domain of *PER2*

| **ID** | **Forward sequence (5’to 3’)** | **Reverse sequence (5’ to 3’)** |
| --- | --- | --- |
| Mut-*PER2*^△PAS1^ | GTTAAGCTTGGTACCGAGCTCGGATCCgccaccATGAATGGATACGCG | CGCCACTGTGCTGGATATCTGCAGAATTCCGTCTGCTCTTCGATCCTGT |
| Mut-*PER2*^△PAS2^ | GTTAAGCTTGGTACCGAGCTCGGATCCgccaccATGAATGGATACGCG | CGCCACTGTGCTGGATATCTGCAGAATTCCGTCTGCTCTTCGATCCTGT |
| Mut-*PER2*^△CT^ | GTTAAGCTTGGTACCGAGCTCGGATCCgccaccATGAATGGATACGCG | GCCACTGTGCTGGATATCTGCAGAATTCGGAGGTCTGGCTCATAAGGT |

**Table S19.** Antibody information used in the experiment

| **Antibody** | **Catalog NO.** | **Brand** | **Dilution** | |
| --- | --- | --- | --- | --- |
|  |  |  | **IHC** | **WB** |
| PER2 | Ab206377 | Abcam |  | 1:5000 |
| PER2 | HA721036 | HUABIO | 1:400 |  |
| HMGCR | ET1702-41 | HUABIO | 1:100 | 1:5000 |
| Ki-67 | 28074-1-AP | Proteintech | 1:2000 |  |
| MYH9 | 60233-1-Ig | Proteintech | 1:1000 | 1:10000 |
| TRIM21 | 12108-1-AP | Proteintech |  | 1:10000 |
| Flag | 20543-1-AP | Proteintech |  | 1:50000 |
| Ubiquitin | ET1609-21 | HUABIO |  | 1:5000 |
| β-actin | 66009-1-Ig | Proteintech |  | 1:50000 |

**Table S20.** Primer sequences for RT-qPCR

| **Gene** | **Forward primer sequence (5’to 3’)** | **Reverse primer sequence (5’ to 3’)** |
| --- | --- | --- |
| PER2 | TATGGTGACTTCCCTTTGC | ATGTGATGTGGGCTTGG |
| HMGCR | TGCCGAGCCTAATGAAAG | CGTGGGTCCATCAAAGAG |
| MYH9 | TCAACACCGACCTGAACCT | CAGTGCCCTCCATCTCCT |
| β-actin | CTTCTACAATGAGCTGCGTGTG | AGAGGCGTACAGGGATAGCACAG |

**Table S21.**  Antibodies for immunofluorescence

| **Antibody** | **Catalog NO.** | **Brand** | **Dilution ratio** |
| --- | --- | --- | --- |
| CoraLite® Plus 488-conjugated PER2 | CL488-67513 | Proteintech | 1:300 |
| Anti-Mouse IgG Alexa Fluor 555 | Ab150114 | Abcam | 1:1000 |
| Anti-Rabbit IgG Alexa Fluor 647 | Ab150079 | Abcam | 1:500 |
| TRIM21 | 12108-1-AP | Proteintech | 1:500 |
| MYH9 | 11128-1-AP | Proteintech | 1:300 |
| MYH9 | 60233-1-lg | Proteintech | 1:300 |
